# Uncovering gaps in workforce well-being: a national look at survey practice in Dutch university medical centres – an exploratory quantitative study

**DOI:** 10.1136/bmjopen-2024-094939

**Published:** 2025-07-18

**Authors:** A.C.P. Boskma, M.S. Oerbekke, L. Hooft, A. Franx, W. Schaufeli, M.J. van der Laan

**Affiliations:** 1 Department of Surgery, UMCG, Groningen, The Netherlands; 2 Netherlands Federation of University Medical Centers, Utrecht, The Netherlands; 3 Cochrane Netherlands, Julius Center for Health Sciences and Primary Care, UMC Utrecht, Utrecht, The Netherlands; 4 Department of Obstetrics & Gynaecology, Erasmus UMC, Rotterdam, The Netherlands; 5 KU Leuven, Leuven, Belgium; 6 Utrecht University, Utrecht, The Netherlands

**Keywords:** Health policy, Occupational Health Services, Burnout, Professional, Caregivers, Caregiver Burden

## Abstract

**Abstract:**

**Introduction:**

Maintaining a healthy workforce is crucial for safe, high-quality care. To enhance well-being and engagement in Dutch university medical centres (UMCs), an overview of staff well-being and job perceptions is needed first. Surveys are widely used to improve working conditions, but varying questionnaires hinder a comprehensive view. This study aimed to evaluate the content of employee surveys currently used in UMCs in the Netherlands from a well-being perspective and to analyse the survey results at a national level.

**Methods:**

All seven UMCs were approached to participate in the study and share employee survey data. The primary outcome of interest is work experience; a secondary analysis was conducted. Items were categorised following the Job Demands-Resources model. Descriptive statistics were presented as percentages, means and medians with IQRs.

**Results:**

Two UMCs participated and 31 862 completed surveys were included. Variation in survey items (eg, 15–18 subcategories, 21–33 question items), response options (eg, 1–5, 1–10), frequency (1–3 times per year) and timing were found. Scores on the following outcomes are presented: work overload, coworker support, job control, organisational justice, participation in decision-making, performance feedback, possibilities for learning and development, recognition, task variety, team atmosphere, team effectiveness, trust in leadership, other job resources, connecting/inspiring leadership, self-efficacy, goal-directiveness, boredom, burnout, job satisfaction, work engagement, other employee well-being, commitment organisation/team and work ability. Results should be interpreted with caution, and solely found for hospital A, for certain job control items, median scores of 2 or 3 were observed, whereas the majority of other question items revealed a median score of 4.

**Conclusions:**

There is a significant lack of cohesion across employee surveys. As it stands, employee surveys in Dutch UMCs are not effective tools for monitoring the work experience or well-being of the healthcare workforce. While these surveys may support management decisions, this support is not reflected in interventions related to work and the work environment.

**Strengths and limitations of this study**

- This study assessed the content of employee surveys currently used in university medical centres (UMCs) in the Netherlands from a well-being perspective and explored survey results at the national level.
- Employee survey data from two hospitals were aggregated with appropriate descriptive statistics applied to minimise information loss from categorical Likert scales.
- The use of existing survey data from unvalidated questionnaires limited internal validity, which affected the reliability and interpretation of the results.
- Due to heterogeneity, categories were inductively and qualitatively formulated, leading to the exclusion of certain question items and restricting the study to an observational approach with descriptive analysis.
- The participation of only two out of seven UMCs introduced selection bias and respondent overlap, reducing external validity and limiting the generalisability of findings for decision-making and practice recommendations.

## Introduction

### Pressure on healthcare systems

 Healthcare systems worldwide are under pressure due to an ageing population,^1 2^ rising chronic diseases^1^ and modern lifestyle challenges.^3^ Moreover, social expectations are rising since today’s patients demand participation and co-creation and expect high-quality personalised care, further straining the system.^4^ At the same time, the number of healthcare professionals (HCPs) is declining. This is due to fewer students pursuing the field^5^ and high attrition rates caused by sick leave, turnover and professionals leaving the field.^6 7^ The healthcare sector shows a relatively high rate of sick leave compared with other sectors, 7.8% in 2024.^8^ One in five employees report that their sick leave was wholly or partly work-related, with high work pressure being the most cited cause.^8^ Retention and turnover are complex processes and vary per HCP type.^9^ Generally, there is no single reason for staying or leaving.^9^ Examples of recognised influencing factors include organisational factors (eg, working hours and adequate staffing), work-related factors (eg, responsibility and shared values), relationship factors (eg, team climate and supportiveness) and recognition factors (eg, salary).^9 10^ Considering the challenges surrounding healthcare demand, reduced influx of HCPs and high professional turnover, it is crucial to prioritise HCPs’ well-being and their work environment.^10^

### Well-being at work

The Job Demands-Resources model (JD-R model) offers a dynamic framework that highlights well-being at work processes.^11^ The JD-R model components are ‘job demands’ (eg, stress, workload, conflicts), ‘job resources’ (eg, support, development opportunities, team atmosphere), ‘leadership’ (eg, inspiring, connecting) and ‘personal resources’ (eg, motivation, resilience).^11^ A balance in these components influences well-being, both positively (eg, job satisfaction) and negatively (eg, burnout), and is reflected in outcomes such as commitment, intention to stay and performance.^11^

### The role of interventions

Research and clinical practice often emphasise individually targeted interventions (eg, coaching, education, behavioural change) to improve HCPs’ well-being.^12^ However, organisational change may offer more proactive and preventive solutions.^13^ Systemic changes can address underlying root causes, benefit larger groups, act as a preventive measure and prove more sustainable.^1214^,^16^ For such changes to be effective, they should be accessible, effective, confidential and tailored to the specific needs of HCPs.^17^ Hospitals often use employee well-being surveys to identify and act on such needs. In Europe, organisations are legally obligated to assess and address psychological safety risks under occupational health and safety laws.

### Surveying HCPs

Systematically surveying employees is widespread practice in most medical centres in The Netherlands. In Europe, it is mandatory according to occupational health and safety laws to address psychological safety risks and act on them. Over the last few decades, such surveys have evolved.^18 19^ Initially, these surveys focused on job satisfaction, where emphasis later shifted to benchmarking (comparing organisations performance). However, benchmarking diverts the attention from the organisation’s own context,^18 19^ and simply having satisfied employees is no longer sufficient.^18^ Instead, it is more important to engage employees, acknowledging their contributions to the hospital’s success, that they feel respected and appreciated, and that they have development and career opportunities.^18^ Nowadays, employee surveys are intended to create insight, and these should be used to improve the working environment, well-being and engagement of employees.^18 19^ Although many employee surveys exist,^20^ previous research has primarily focused on their content and implementation.^21^ Contexts and perceptions of HCP changes over time.^22^ Rather than developing new tools, it is advised to analyse and optimise existing ones.^2022^,^24^ Besides, approaches are focused on work environment experiences, and with this, the well-being of HCPs is less centred. The questionnaires are intended as a tool to initiate dialogue with teams and employees about these aspects.^19^ This is particularly relevant in academic medical centres, where professionals face complex tasks due to the complex patient population. Their role in treating the most complex cases and training future HCPs increases the organisational demands and highlights the importance of maintaining a healthy, sustainable workforce. This makes structured evaluation of well-being through employee surveys especially valuable. Though not mandatory, employee surveys are cyclically administered in Dutch hospitals, ranging from once every 2 years to four times per year.

### Evaluating Dutch surveys and analysing results at a national level

To support the sustainable employability of HCPs in Dutch academic hospitals, an overview of their well-being and job perceptions is necessary. Although there are uniform legal obligations, different questionnaires are used at various moments across institutions, limiting straightforward syntheses and obstructing a bigger picture. This study, therefore, focuses on two comprehensive and reasonably comparable hospitals, selecting data from direct care providers (eg, nurses, doctors), supportive roles (eg, assistants, administrative staff) and education/research/management staff. The aim of this study is to evaluate the content of employee surveys currently used in university medical centres (UMCs) in the Netherlands from a well-being perspective and to analyse the survey results at a national level.

## Methods

### Study design

This study is multicentred and used existing longitudinal data from employee surveys. The Strengthening the Reporting of Observational Studies in Epidemiology Statement^25^ was used to facilitate reporting the results. The study consisted of two parts: in the preparation phase 1, the content of employee surveys was assessed, and in the execution phase 2, the survey results were explored of two UMCs. In this study, it was decided to avoid benchmarking, comparisons between hospitals and not to formulate definitive conclusions due to the heterogeneity of the data. Additionally, we wanted to avoid competition and instead emphasise learning from other centres and focusing on elements related to the work context.

### Setting and respondents

All seven UMCs were approached to participate in the study and share employee survey items and employee survey results. The population consisted of all hospital employees up to 65 years of age from all seven Dutch UMCs.

### Data collection

This study aimed to re-use existing data. The advantage of this approach was the ability to evaluate existing (non-validated) surveys without placing an additional burden on employees to obtain a national-level overview. It was assumed in advance that well-being would be assessed, at least in part, in the available surveys. This would allow for a comparable analysis using data that was already collected.

**Figure 1:**
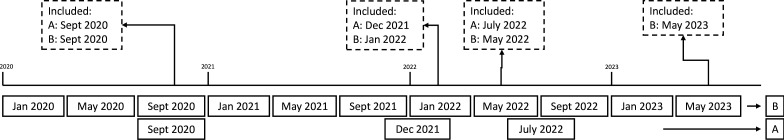
Schematic representation of included measurement moments.

Two UMCs were willing and in the opportunity to participate. Data from 2020 to 2023 were requested through human resource advisors to identify patterns over time and to account for possible fluctuations due to COVID-19 and seasonal variation. Hospital A used three different surveys over the 3 years and hospital B used one survey (online supplemental additional file 1). Hospital A conducted the survey once per year and hospital B three times per year (January, May and September). In total, four different surveys and seven measurement moments were included in the analysis (data during COVID-19 pandemic and data after COVID-19 pandemic). This signifies for hospital A: September 2020, December 2021 and July 2022. For hospital B, the measurement moments best aligning to hospital A were chosen. This signifies: September 2020, January 2022, May 2022 and May 2023. For a schematic representation of included measurement moments, see figure 1. For phase 1, survey items were qualitatively constructed by AB and MO following the JD-R model^11^ to explore if questions were included addressing: job demands (eg, workload, work–life balance), job resources (eg, sustainable employability, recognition/appreciation, job content, development opportunities, psychological safety, support, team), personal resources (eg, resilience, goal directiveness), leadership (eg, autonomy, leadership), employee well-being (eg, job satisfaction) and outcomes (eg, health, engagement, eNPS (employee Net Promoter Score), intention to leave (the profession), patient safety, improvement). Furthermore, basic characteristics are linked to the survey by administrative data. For phase two, the following employee characteristics were included: the specific hospital where employees work, age, gender, years of service and positions/roles.

Anonymised data at the respondent level was collected through the hospitals’ Human Resource advisors of the hospitals. To prevent employees from being identifiable, it was important that age was categorised into ranges of 10 years (15–25, 25–35, 35–45, 45–55 and 55–65), years of service into ranges of 5 years, and positions/roles were identified according to the following classification: (1) resident physicians (residents and specialist trainees); (2) nursing and care (nurses, caregivers ward assistants); (3) medical specialists; (4) clinical (co)treating (laboratory technicians, physiotherapists, dietitians, psychologists); (5) clinical support (surgical assistants, anaesthesia technicians, medical assistants); (6) facility (eg, technology roles, area management staff, logistics staff); (7) staff, administration and secretariat (eg, staff advisors, secretaries, teachers, research and education staff); (8) scientific research and education (professors, researchers, PhD candidates, MD-PhD); (9) analytical (analysts); (10) management (managers, team leaders, head nurses) and (11) trainees/students and (12) others.

Data were managed using Excel and SPSS Statistics V.28 (IBM). Survey questions were initially translated through Google Translate and Deepl.com, and all original questions with their translations were assessed by AB and MO to check whether the translated content sufficiently reflected the Dutch questions and to determine their final English wording.

### Data handling

The first step of structuring the data consisted of determining overlapping themes within the four instruments to gain insight into which categories are appropriate for illustrating the big picture. Two researchers independently categorised survey questions into categories and subcategories reported in the ‘energy compass’ (derived from the JD-R model)^11^ and resolved discrepancies through discussion. Scores of question items were presented per category in a table. Only data from completed surveys at any given measurement moment were included in the analyses. In this study, complete surveys are referred to since individual respondents were probably included multiple times because they completed several surveys at measurement moments. Only subcategories and survey questions measured, at least on two different measurement moments, were included to ensure more robustness and to enable exploration of data over time. With this data from the following categories, were excluded from the analysis: communication, emotional demand, physical demand, mental demand, harassment, sleep problems, use of skills and work–home conflict.

### Data analysis

Within hospitals and across measurement moments, data from the same individuals may be present. Repeated measures were included in the analysis. However, due to the anonymous nature of the data, it was not possible to link individual responses over time. Therefore, the data were treated as repeated cross-sectional observations. Descriptive analyses were used to explore trends and patterns across years. Descriptive statistics were presented as means for continuous variables and medians with IQRs for categorical variables. Additionally, the full distribution of responses per item was provided in percentages to offer insight into response spread and agreement levels.

A stratification by age and positions/roles was conducted for work overload and opportunities for learning and development, as these categories were included by both hospitals in all seven measurement moments. Individual participant characteristics were summarised using descriptive statistics. Areas for improvement are determined relatively and inductively, based on lower median scores and broader variability (IQR) in comparison to other items. No formal hypothesis testing was conducted since such testing was not feasible due to the heterogeneity of the data across hospitals and time points.

## Results

### Participant characteristics

Hospital A contains more than 12 000 employees and 1300 beds. Hospital B contains more than 11 000 employees and 1000 beds. Reasons for the other five UMCs to decline were staff shortage in the Information Technology department, doubts about obtaining proper results (eg, due to heterogeneity in methods, content, time frame and purpose of the surveys) and insufficient trust in the added value of transparency between centres.

A total of 31 862 completed surveys were included, encompassing 11 862 for hospital A and 19 687 for hospital B. This corresponds to 3603–5056 fully completed surveys at each measurement moment. Table 1 illustrates the distribution of complete surveys per hospital per measurement moment. Hospital A has a consistently lower response rate (around 30%–35%) compared with hospital B (around 44%). Descriptive characteristics are shown for gender, age, years of service and positions/roles. In hospital A, most complete surveys are filled in by males, while in hospital B, there is a large amount of missing gender data. Missing values for participant characteristics have not been imputed, due to huge numbers, and these data were not provided by respondents. The data were linked through an HR system and, therefore, is independent of any potential selection bias. Age is similar across both hospitals, with the largest groups being aged 30–59. Most respondents have 0–5 years of service experience, particularly in hospital B. The most common positions/roles are staff, administration, secretarial and nursing and care across both hospitals.

**Table 1:** Basic characteristics of the sample

		Hospital A	Hospital A	Hospital A	Hospital B	Hospital B	Hospital B	Hospital B
		September 2020	December 2021	July 2022	September 2020	January 2022	May 2022	May 2023
	Total	11.757	11.958	11.875	11.316	11.183	11.121	11.059
	Complete cases	4157	4102	3603	5056	4842	4894	4895
	Response percentage	35.36%	34.30%	30.34%	44.68%	43.30%	44.01%	44.26%
Gender								
Male	Complete cases	2993 (72.00%)	3013 (73.45%)	2630 (72.99%)	149 (2.95%)	138 (2.85%)	169 (3.45%)	178 (3.64%)
Female	Complete cases	1164 (28.00%)	1089 (26.55%)	973 (27.01%)	442 (8.74%)	464 (9.58%)	505 (10.32%)	486 (9.93%)
	Missing values for gender	0 (0.00%)	0 (0.00%)	0 (0.00%)	4465 (88.31%)	4240 (87.57%)	4220 (86.23%)	4231 (86.44%)
Age in years								
<30	Complete cases	569 (13.69%)	556 (13.55%)	473 (13.13%)	1039 (20.55%)	928 (19.17%)	907 (18.53%)	919 (18.77%)
30–39	Complete cases	941 (22.64%)	919 (22.40%)	781 (21.68%)	1157 (22.88%)	1116 (23.05%)	1146 (23.42%)	1166 (23.82%)
40–49	Complete cases	970 (23.33%)	1067 (26.01%)	932 (25.87%)	985 (19.48%)	976 (20.16%)	1030 (21.05%)	1023 (20.90%)
50–59	Complete cases	1173 (28.22%)	1100 (26.82%)	1004 (27.87%)	1107 (21.89%)	1074 (22.18%)	1066 (21.78%)	1085 (22.17%)
60+	Complete cases	504 (12.12%)	460 (11.21%)	413 (11.46%)	481 (9.51%)	446 (9.21%)	435 (8.89%)	412 (8.42%)
	Missing values for age	0 (0.00%)	0 (0.00%)	0 (0.00%)	287 (5.68%)	302 (6.24%)	310 (6.33%)	290 (5.92%)
Years of service								
0–5	Complete cases	1369 (32.93%)	1406 (34.28%)	1211 (33.61%)	2305 (45.59%)	2200 (45.44%)	2275 (46.49%)	2241 (45.78%)
6–10	Complete cases	599 (14.07%)	564 (13.75%)	521 (14.46%)	712 (14.08%)	682 (14.09%)	654 (13.36%)	693 (14.16%)
11–15	Complete cases	616 (14.47%)	644 (15.70%)	539 (14.96%)	664 (13.13%)	595 (12.29%)	648 (13.24%)	605 (12.36%)
16–20	Complete cases	593 (13.93%)	537 (13.09%)	467 (12.96%)	465 (9.20%)	477 (9.85%)	484 (9.89%)	493 (10.07%)
21–25	Complete cases	311 (7.31%)	342 (8.34%)	355 (9.85%)	455 (9.00%)	486 (10.04%)	436 (8.91%)	459 (9.38%)
26–30	Complete cases	244 (5.73%)	197 (4.80%)	155 (4.30%)	189 (3.74%)	161 (3.33%)	164 (3.35%)	166 (3.39%)
31–35	Complete cases	256 (6.01%)	245 (5.97%)	196 (5.44%)	130 (2.57%)	128 (2.64%)	111 (2.27%)	117 (2.39%)
36–40	Complete cases	100 (2.35%)	100 (2.44%)	98 (2.72%)	60 (1.19%)	62 (1.28%)	65 (1.33%)	62 (1.27%)
41–45	Complete cases	64 (1.50%)	62 (1.51%)	55 (1.53%)	25 (0.49%)	21 (0.43%)	24 (0.49%)	20 (0.41%)
46>	Complete cases	5 (0.12%)	5 (0.12%)	6 (0.17%)	1 (0.02%)	0 (0.00%)	3 (0.06%)	3 (0.06%)
	Missing values for years of service	0 (0.00%)	0 (0.00%)	0 (0.00%)	50 (0.99%)	30 (0.62%)	30 (0.61%)	36 (0.74%)
Function								
Nursing and care	Complete cases	892 (20.95%)	871 (21.23%)	764 (21.20%)	821 (16.24%)	726 (14.99%)	803 (16.41%)	804 (16.42%)
Clinical support	Complete cases	365 (8.57%)	336 (8.19%)	307 (8.52%)	418 (8.27%)	385 (7.95%)	416 (8.50%)	412 (8.42%)
Clinical (co) treating	Complete cases	278 (6.53%)	250 (6.09%)	215 (5.97%)	309 (6.11%)	331 (6.84%)	335 (6.85%)	348 (7.11%)
Analytics	Complete cases	223 (5.24%)	277 (6.75%)	229 (6.36%)	424 (8.39%)	375 (7.74%)	393 (8.03%)	418 (8.54%)
Scientific research and education	Complete cases	289 (6.79%)	307 (7.48%)	243 (6.74%)	373 (7.38%)	387 (7.99%)	358 (7.32%)	402 (8.21%)
Management	Complete cases	225 (5.29%)	218 (5.31%)	219 (6.08%)	205 (4.05%)	197 (4.07%)	199 (4.07%)	173 (9.66%)
Staff, administration, secretariat	Complete cases	1130 (26.54%)	1104 (26.91%)	991 (27.50%)	1111 (21.97%)	1116 (23.05%)	1126 (23.01%)	1136 (23.21%)
Facility	Complete cases	376 (8.83%)	370 (9.02%)	300 (8.33%)	656 (12.97%)	598 (12.35%)	595 (12.16%)	570 (11.64%)
Resident physicians	Complete cases	73 (1.71%)	75 (1.83%)	62 (1.72%)	180 (3.56%)	156 (3.22%)	133 (2.72%)	149 (3.04%)
Medical specialists	Complete cases	261 (6.13%)	254 (6.19%)	242 (6.72%)	323 (6.39%)	340 (7.02%)	318 (6.50%)	266 (5.43%)
Students and others	Complete cases	45 (1.06%)	40 (9.98%)	31 (0.86%)	236 (4.67%)	231 (4.77%)	218 (4.45%)	217 (4.43%)
	Missing values for function	0 (0.00%)	0 (0.00%)	0 (0.00%)	0 (0.00%)	0 (0.00%)	0 (0.00%)	0 (0.00%)

### Phase 1: content and constructs of employee surveys

Variation in survey items, response options, frequency and timing were found. Following the JD-R categories, in total we included 18 subcategories for hospital A and 15 for hospital B (see table 2). The constructs most frequently addressed in employee surveys predominantly pertain to the subcategory of job resources. Some themes are assessed in both hospitals, which may indicate a key set of shared priorities or standards across the two hospitals. Categories such as personal resources are under-represented, which may suggest a potential blind spot in capturing broader aspects of employee well-being. Categories were often explored through multiple questions and analysed by means of statistical methods, despite the fact that answer options were ordinally distributed within categories. Hospital A used multiple questions in one survey for the following subcategories: coworker support (four items), job control (five items), recognition (three items), team atmosphere (two items) and team effectiveness (two items). Hospital B used multiple questions for subcategories: performance feedback (two items), possibilities for learning and development (three items), team effectiveness (five items), trust in leadership (two items), other job resources (three items) and commitment organisation (two items). For an overview of overlapping and differences in subcategories between hospitals, see table 2, but this did not mean that within each subcategory the questions agreed between hospitals.

**Table 2:** Overview of overlapping and differences in subcategories between hospitals

	Hospital A	Hospital B
Job demands		
Workload	X	X
Job resources		
Coworker support	X	
Job control	X	
Organisational justice		X
Participation in decision-making		X
Performance feedback	X	X
Possibilities for learning and development	X	X
Recognition	X	
Task variety	X	
Team atmosphere	X	
Team effectiveness	X	X
Trust in leadership		X
Other job resources	X	X
Engaged leadership		
Connecting	X	
Inspiring	X	X
Personal resources		
Self-efficacy		X
Goal directedness		X
Employee well-being		
Boredom	X	
Burnout	X	
Job satisfaction	X	X
Work engagement	X	
Other employee well-being		X
Outcomes		
Commitment organisation	X	X
Commitment team		X
Work ability	X	

### Phase 2: survey results at a national level

Overall, the surveys use 5-point Likert-type scales, where response options typically range from (1) totally disagree to (5) totally agree. A sixth option (6) is included and varies by item as either no opinion or not applicable. A median score of 4 indicates agreement or a positive experience. A median of 3 reflects neutral or mixed perceptions. A median of 2 indicates disagreement or a negative experience. In online supplemental additional file 2, an overview of survey results is presented per survey item and measurement. A more comprehensive overview of results is addressed in online supplemental additional file 3, including a distribution for all answer options per surveyed question. The insights are concisely described in the results for clarity. A complete description of results per JD-R category is reported in online supplemental additional file 4.

#### Work environment

Across both hospitals, workload was generally perceived as acceptable to appropriate. However, in hospital B, responses were more varied (median 5, IQR 2–5), suggesting a broader range of experiences with workload appropriateness. In hospital A, coworker support was consistently rated positively, particularly within teams (median 4, IQR 4–5). In contrast, support across the care chain (median 4, IQR 3–4) and during difficult situations (median 4, IQR 3–5) was perceived less uniformly, pointing to more mixed experiences in these areas. Job control showed the most variability in perceptions, especially in hospital A. Respondents reported limited autonomy over work timing (median 2, IQR 2–4), tempo (median 3, IQR 2–4) and breaks (median 3, IQR 2–4), reflecting low to neutral agreement and suggesting that this is an area requiring attention. The inconsistency was also evident from discrepancies between the median and most frequently selected answers. Perceptions of organisational justice (median 4, IQR 4–4) and participation in decision-making (median 4, IQR 3–4) in hospital B were stable and indicated general agreement. Similarly, both hospitals showed consistent access to performance feedback (median 4, IQR 3–4) and learning and development opportunities (median 4, IQR 3–4). A notable exception was found in hospital A, where the item ‘chance to learn and develop my knowledge and skills’ received more varied responses (median 3, IQR 2–4), although the most common answer remained positive (4, or ‘agree’, in 31.89% of cases). Recognition (median 4, IQRs 3–4 and 4–5), task variety (median 4, IQR 4–5) and team atmosphere (median 4, IQRs 4–4 and 4–5) were rated positively and consistently over time in hospital A, showing strong agreement. However, slightly greater variation in ratings for relationships across the care chain compared with within teams suggests differences in perceived collaboration. Team effectiveness received overall positive ratings in both hospitals (median 4, IQRs 3–4 and 4–4), though in hospital A, perceived effectiveness across the care chain was lower (median 3, IQR 3–4), indicating weaker collaboration in that area. Trust in leadership was generally present in hospital B (median 4, IQR 3–4), though perceptions of individual managers varied more widely (median 4, IQR 3–6), possibly influenced by frequent selection of ‘no opinion’. Finally, both hospitals consistently reported a positive work environment and effective collaboration across departments (median 4, IQR 3–4), underscoring a generally favourable organisational climate.

#### Individual HCP

Connecting leadership in hospital A was consistently perceived as present over time, reflected in stable agreement levels (median 4, IQR 4–5). Inspiring leadership showed more neutral perceptions in both hospitals (median 4, IQR 3–4), with some variation in motivation by supervisors; the item ‘my supervisor motivates me’ received slightly lower ratings (median 3, IQR 3–4), indicating moderate to neutral agreement. Self-efficacy and goal-directedness in hospital B were found to be generally present, with a median score of 4 (IQR 3–4). Perceptions of boredom in hospital A fluctuated across years. Ratings were more favourable in 2020 and 2022 (median 4, IQR 4–4), while a dip was observed in 2021 (median 3, IQR 3–4), indicating a temporary increase in experienced boredom. Nevertheless, the most frequently chosen response remained ‘often’ (score 4), selected by 39.54% of respondents, pointing to a generally recurring experience of boredom. Burnout levels in hospital A remained stable over time, with nearly identical mean scores (6.5 in 2021; 6.4 in 2022), suggesting no notable change in burnout symptoms. Job satisfaction was consistently rated positively across both hospitals (median 4, IQR 4–5), indicating general agreement with satisfaction statements. Notably, answer option 6 (‘no opinion or not applicable’) was rarely selected, further supporting the strength of the agreement. Work engagement in hospital A showed a slight downward trend over time, with mean scores decreasing from 7.14 in 2021 to 6.94 in 2022, suggesting a minor decline in energy and involvement. Perceived safety at work in hospital B was found to be generally affirmed, with a median score of 4 (IQR 4–5). Organisational commitment was rated with a median score of 4 (IQR 3–4), with stable mean values in hospital B (range 7.29–7.31), reflecting general agreement regarding organisational commitment. Commitment to the team in hospital B was also found to be generally present, with a median score of 4 (IQR 3–4), showing stable agreement across timepoints. Finally, workability in hospital A was found to be consistently moderate to positive throughout the measurement period, with a median score of 4 (IQR 3–4), indicating a generally stable perceived capacity to perform job tasks.

### Stratification by age and positions/roles

Detailed stratification by age and positions/roles is illustrated in online supplemental additional file 5.

#### Work overload

In hospital A, multiple groups showed lower ratings for workload, with a median score of 3 (neutral, IQR 2–4), indicating greater variability within these groups and that a substantial number of employees rated the workload as less than acceptable. This was observed among employees aged 60+, as well as those working in nursing and care, clinical support, scientific research and education, and medical specialists. In addition, for the second item about workload, nearly every group indicated there is too much work.

In hospital B, lower agreement with appropriate workload was seen among employees aged 40–49, and those in clinical support, clinical (co)treating roles, scientific research and education and medical specialists.

#### Possibilities for learning and development

In hospital A, most groups reported agreement regarding learning and development opportunities (median 4, IQR 3–4). Deviations from this pattern were found in the analytics group (median 3, IQR 3–4), indicating that at least half of this group expressed lower levels of agreement. Groups such as employees under 30, those in management roles, and resident physicians showed a more uniform pattern (median 4, IQR 4–4), suggesting general agreement with limited variability. Higher levels of agreement were observed in groups involved in scientific research and education, management and among medical specialists (median 4, IQR 4–5). The groups most frequently expressing disagreement with the statement (>3%) included those aged 50–59, and those working in clinical support, facilities, clinical (co)treating roles and analytics.

A similar pattern was found in hospital B. Most groups reported agreement (median 4, IQR 3–4), although in January 2022, employees under 30 showed a lower rating (median 3). Groups such as those aged <30, 30–39, 40–49 and 60+, and those in clinical (co)treating roles, scientific research and education, management, staff/administration/secretariat, resident physicians, and medical specialists showed limited variability (median 4, IQR 4–4). A higher level of agreement was again observed among those in scientific research and education and resident physicians (median 4, IQR 4–5). The groups most frequently disagreeing with the statement (>3%) included those aged 40–49 and 60+, as well as those working in clinical support, analytics, management and facilities.

## Discussion

This study aimed to assess the content and methodological consistency of employee well-being surveys currently used in Dutch UMCs, and to explore national-level survey results from two participating hospitals as a secondary step. Phase 1 focused on evaluating the design, scope and theoretical alignment of surveys from a well-being-at work perspective. Phase 2 involved a descriptive analysis of aggregated survey results, offering initial insights into reported employee experiences.

### Survey design and methodological variation

Substantial variation was found across UMCs in terms of survey content, item phrasing, response options, timing and frequency. While surveys covered a wide array of topics, the focus of the questions often reflected strategic priorities of hospital boards rather than the focus of implemented interventions. These findings align with existing literature describing the trade-offs between comprehensive validated instruments and shorter, pragmatic tools that are easier to administer but less robust psychometrically.^24 26^ The diversity of survey designs may be influenced by local leadership styles, cultural norms and shifting organisational priorities. While this flexibility can help capture emerging topics, such as inclusivity and psychological safety,^22^ it limits comparability across organisations and over time, undermining the potential for data-informed decision-making.

### Challenges in interpreting results

The exploratory analysis of survey results from hospital A highlighted several domains (particularly job control, learning and development, team effectiveness and inspiring leadership) as potential areas for improvement. Items in these domains scored a median of 2 or 3, compared with 4 on most other topics. While this provides a starting point for discussion, the interpretation must be cautious due to limitations in survey design, measurement reliability and external validity. Moreover, these data only represent a subset of UMC employees and likely suffer from response bias and overlap in respondents. Nevertheless, despite the limitations in interpreting the current study’s results, previous research has consistently emphasised the importance of, for example, job control and autonomy, which supports the relevance and credibility of the observed patterns.^27^,^29^

Notably, we found minimal differences in outcomes over time, including during and after the COVID-19 period. This is inconsistent with literature showing significant declines in well-being among HCPs during the pandemic.^27 30 31^ Several explanations are possible: survey items may lack sensitivity to change,^24^ the surveys may not capture key stressors, or a relatively short timeframe and socially desirable responding may have masked real variation. These issues point to the need for clinimetric research into the validity, reliability and responsiveness of existing tools.^32^

### Misalignment between measurement and action

A key finding is the disconnect between what is measured and what is targeted by interventions. Most survey items focus on job resources, such as supervisory support and team climate, while organisational interventions tend to target personal resources like resilience or stress management. This misalignment may partly explain why organisations struggle to measure intervention impact or drive meaningful change through survey data. One possible explanation is that survey design is often human resources or board-driven, while interventions are mostly shaped by project leaders, consultants or by HCPs themselves, resulting in a disconnect between assessment and response.

### Strengths and limitations

This study offers early insights into both the content of well-being surveys and the patterns they reveal. A strength of our approach is the descriptive use of distributions and medians instead of means, which are often inappropriately applied to ordinal data. However, significant limitations remain. First, internal validity decreased since data was used from unvalidated heterogeneous surveys. This influences the reliability of the results and requires caution with interpreting the results.^32^ Second, the inductive categorisation process, although conducted independently by the authors, introduces the risk of inconsistency and subjectivity. Furthermore, only two of the seven Dutch UMCs participated, greatly reducing generalisability. Overlapping respondents and selection bias may have further skewed results.^32^ Combined, these limitations constrain our ability to draw robust conclusions and diminish the utility of findings for policy or practice.

### Practical implications

Several practical implications emerge:

- Survey frequency should be re-evaluated, as repeated measurements showed little variation. Oversurveying may burden staff without producing actionable insights.^18^
- Job control emerged as a key area for improvement. However, it is important to ensure that this and other areas identified through surveys are within employees’ sphere of influence and reflect their lived experiences.
- Current surveys are broad and suited for organisational reflection, but they may miss critical indicators like performance, turnover intention and resource adequacy. A more targeted, theory-driven approach is needed for intervention planning.
- Using validated instruments that can detect changes over time and adopting a shared conceptual framework (eg, the JD-R model) can support more meaningful benchmarking and evidence-informed strategies.

Collaborative efforts between hospitals could support the development of a core outcome set, enabling collective learning and longitudinal monitoring. Without such alignment, surveys may continue to serve primarily symbolic or communicative functions rather than actionable tools for change.^26^

## Conclusions

Currently, employee well-being surveys in Dutch UMCs show significant methodological variation, limiting their usefulness for monitoring work experience and informing policy. While they may support internal reflection, their capacity to guide targeted intervention is undermined by weak measurement validity, lack of alignment with organisational actions and limited comparability across centres.

Job control appears to be an area warranting further attention, though interpretation is constrained by methodological issues. The lack of meaningful variation in survey outcomes over time raises important questions about the appropriateness of current instruments and the assumptions guiding their use.

Future research should focus on developing a consensus-based core outcome set (eg, via a Delphi study), and explore why validated tools are underutilised and collaboration is minimal. Rather than developing new instruments, efforts should centre on improving use and integration of existing tools. To support evidence-based people management, the rigour of patient care research must be mirrored in the way we evaluate and support the healthcare workforce.

## Supplementary material

10.1136/bmjopen-2024-094939 online supplemental file 1

10.1136/bmjopen-2024-094939 online supplemental file 2

10.1136/bmjopen-2024-094939 online supplemental file 3

10.1136/bmjopen-2024-094939 online supplemental file 4

10.1136/bmjopen-2024-094939 online supplemental file 5

## Data Availability

All data relevant to the study are included in the article or uploaded as supplementary information.
