# Supplementary material for: Uncovering gaps in workforce well-being: a national look at survey practice in Dutch university medical centres – an exploratory quantitative study
Source: BMJ Open. 2025 Jul 18;15(7):e094939. doi: 10.1136/bmjopen-2024-094939 (PMC12273149; doi:10.1136/bmjopen-2024-094939)
Supplement: online supplemental file 1 [file bmjopen-15-7-s001.docx]

**Additional file 1**

*Table A Surveys question items hospital A 2020*

| Sub-category | Question item | Answer options |
| --- | --- | --- |
| Work ability | How likely are you to recommend the hospital as an employer to friends, acquaintances, or business contacts? | (0) unlikely; (10) likely |
| Task variety | My work is sufficiently varied | (1) totally disagree; (2) disagree; (3) neutral; (4) agree; (5) totally agree; (6) not applicable |
| Job control | I can decide how and when to do my work within reasonable limits |  |
| Boredom | My work is challenging in a good way |  |
| Job satisfaction | I enjoy my work |  |
| Recognition | With my work I am of added value |  |
| Work ability | I can do my job without negative effects on my health |  |
| Co-worker support | As colleagues we help each other (team) |  |
| Team atmosphere | The relationship with my colleagues is good (team) |  |
| Team effectiveness | As colleagues we work together in a smart way (team) |  |
| Co-worker support | As colleagues we help each other (chain) |  |
| Team atmosphere | The relationship with my colleagues is good (chain) |  |
| Team effectiveness | As colleagues we work together in a smart way (chain) |  |
| Connecting | The relationship with my direct supervisor is good |  |
| Performance feedback | I receive sufficient feedback on how i do my work |  |
| Recognition | I receive sufficient appreciation for my work |  |
| Inspiring | My direct supervisor knows how to motivate me |  |
| Possibilities for learning & development | I am given the opportunity to develop |  |
| Other | My work environment is pleasant |  |
| Work overload | There is an acceptable workload |  |
| Organization | Working for this hospital makes me proud |  |

*Table B Surveys question items hospital A 2021*

| Sub-category | Question item | Answer options |
| --- | --- | --- |
| Recognition | I am appreciated at work | (1) totally disagree; (2) disagree; (3) neutral; (4) agree; (5) totally agree; (6) no opinion |
| Co-worker support | I receive help with my work when needed |  |
| Communication | I receive the information I need at work. |  |
| Co-worker support | If I am having a hard time at work I can turn to someone |  |
| Job control | I can decide how i do my work |  |
| Job control | I can decide when i do my work |  |
| Job control | I can set my own work pace |  |
| Participation in decision making | I have influence over decisions in my work. |  |
| Emotional demands | My work is emotionally demanding |  |
| Mental demands | I struggle with my work because it is complicated |  |
| Physical demands | My work is physically demanding |  |
| Work overload | I have too much work |  |
| Job demand | The time pressure in my work is high. |  |
| Work-home conflict | My work has no negative effect on my private life. |  |
| Job control | I can take breaks whenever I need it |  |
| Sleep problems | I wake up feeling refreshed |  |
| Use of skills | I have the opportunity to use my knowledge and skills. |  |
| Possibilities for learning & development | I get opportunity to learn and develop knowledge and skills |  |
| Boredom | My work is challenging in a good way |  |
| Burnout | Indicate where you are on the range of fatigue to vitality | 1-10 ((1) fatigue; (10) vital) |
| Work engagement | Indicate where you are on the balance of disengaged to engaged | 1-10 ((1) disengagement; (10) engagement) |
| Harassment | In your interactions with colleagues or supervisors over the past year, have you personally experienced: Threats or intimidation? | (1) never; (2) sometimes; (3) regularly; (4) often; (5) always; (6) I don’t know |
| Harassment | In your interactions with colleagues or supervisors over the past year, have you personally experienced: Discrimination? |  |
| Harassment | In your interactions with colleagues or supervisors over the past year, have you personally experienced: Bullying? |  |
| Harassment | In your interactions with colleagues or supervisors over the past year, have you personally experienced: Sexual harassment? |  |
| Harassment | In your interactions with colleagues or supervisors over the past year, have you personally experienced: Physical aggression? |  |
| Harassment | In your interactions with colleagues or supervisors over the past year, have you personally experienced: Verbal aggression? |  |
| Harassment | In your interactions with patients, their families, or visitors over the past year, have you personally experienced: Threats or intimidation? |  |
| Harassment | In your interactions with patients, their families, or visitors over the past year, have you personally experienced: Discrimination? |  |
| Harassment | In your interactions with patients, their families, or visitors over the past year, have you personally experienced: Bullying? |  |
| Harassment | In your interactions with patients, their families, or visitors over the past year, have you personally experienced: Sexual harassment? |  |
| Harassment | In your interactions with patients, their families, or visitors over the past year, have you personally experienced: Physical aggression? |  |
| Harassment | In your interactions with patients, their families, or visitors over the past year, have you personally experienced: Verbal aggression? |  |

*Table C Surveys question items hospital A 2022*

| Sub-category | Question item | Answer options |
| --- | --- | --- |
| Burnout | Indicate where you are on the range of fatigue to vitality | 1-10 ((1) fatigue; (10) vital) |
| Work engagement | Indicate where you are on the balance of disengaged to engaged | 1-10 ((1) disengagement; (10) engagement) |
| Task variety | My work is sufficiently varied | (1) totally disagree; (2) disagree; (3) neutral; (4) agree; (5) totally agree; (6) not applicable |
| Job control | I can decide how and when to do my work within reasonable limits |  |
| Boredom | My work is challenging in a good way |  |
| Job satisfaction | I enjoy my work |  |
| Recognition | With my work I am of added value |  |
| Work ability | I can do my job without negative effects on my health |  |
| Co-worker support | As colleagues we help each other (team) |  |
| Team atmosphere | The relationship with my colleagues is good (team) |  |
| Team effectiveness | As colleagues we work together in a smart way (team) |  |
| Co-worker support | As colleagues we help each other (chain) |  |
| Team atmosphere | The relationship with my colleagues is good (chain) |  |
| Team effectiveness | As colleagues we work together in a smart way (chain) |  |
| Connecting | The relationship with my direct supervisor is good |  |
| Performance feedback | I receive sufficient feedback on how i do my work |  |
| Recognition | I receive sufficient appreciation for my work |  |
| Inspiring | My direct supervisor knows how to motivate me |  |
| Possibilities for learning & development | I am given the opportunity to develop |  |
| Other | My work environment is pleasant |  |
| Work overload | There is an acceptable workload |  |
| Organization | Working for this hospital makes me proud |  |

*Table D Surveys question items hospital B*

| Sub-category | Question item | Answer options |
| --- | --- | --- |
| Job satisfaction | I enjoy my work | (1) totally disagree; (2) disagree; (3) neutral; (4) agree; (5) totally agree; (6) no opinion |
| Other | I feel safe at work |  |
| Organizational justice | I can address mistakes and unsafe situations without fear of negative consequences |  |
| Participation in decision making | I feel free to question decisions or actions of persons with greater authority |  |
| Work overload | I think my workload is on an average base.. | (1) way too much, (2) too much, (3) too less, (4) way too less, (5) appropriate; (6) no opinion |
| Possibilities for learning & development | I can develop in my work | (1) totally disagree; (2) disagree; (3) neutral; (4) agree; (5) totally agree; (6) no opinion |
| Possibilities for learning & development | I can continuously improve in my work |  |
| Self-efficacy | I can effectively solve problems in my work |  |
| Possibilities for learning & development | Within our team we learn from mistakes |  |
| Team effectiviness | Within our team we adhere to the agreements we make with each other |  |
| Team effectiviness | Within our team we openly share knowledge and information |  |
| Performance feedback | Within our team it is common to give feedback |  |
| Performance feedback | The feedback of my direct supervisor helps me to improve my work |  |
| Trust in leadership | My direct supervisor provides good leadership |  |
| Inspiring | My direct supervisor shows exemplary behavior |  |
| Trust in leadership | My manager provides good leadership |  |
| Team effectiviness | I know the goals of our team |  |
| Goal directedness | I know what I need to do to achieve our team's goals |  |
| Team effectiviness | Within our team we use the results of the employee survey to make improvements |  |
| Organization | I like to do something extra for my work |  |
| Team | I put my team's results above my personal ambitions |  |
| Organization | The success of my hospital means much to me | 1-10 ((1) bad; (2) good) |
