# Supplementary material for: Uncovering gaps in workforce well-being: a national look at survey practice in Dutch university medical centres – an exploratory quantitative study
Source: BMJ Open. 2025 Jul 18;15(7):e094939. doi: 10.1136/bmjopen-2024-094939 (PMC12273149; doi:10.1136/bmjopen-2024-094939)
Supplement: online supplemental file 2 [file bmjopen-15-7-s002.docx]

**Additional file 2**

*Table E: median and means per question item for diverse measurement moments within hospital A and B, categorized following the JD-R model*

| **Sub-category** | **Question-item** | **Scale** | **Hospital A** | **Hospital A** | **Hospital A** | **Hospital B** | **Hospital B** | **Hospital B** | **Hospital B** |
| --- | --- | --- | --- | --- | --- | --- | --- | --- | --- |
|  |  |  | *Sept 2020 (4157)* | *Dec 2021 (4102)* | *July 2022 (3603)* | *Sept 2020 (5056)* | *Jan 2022 (4842)* | *May 2022 (4894)* | *May 2023 (4895)* |
| Job demands | | | | | | | | | |
| Work overload | There is an acceptable workload | (1) totally disagree; (2) disagree; (3) neutral; (4) agree; (5) totally agree; (6) not applicable | 4 (IQR: 3-4) |  | 4 (IQR: 3-4) |  |  |  |  |
|  | I have too much work | (1) never; (2) sometimes; (3) regularly; (4) often; (5) always; (6) I don’t know |  | 4 (IQR: 3-4) |  |  |  |  |  |
|  | I think my workload is on an average base.. | (1) way too much, (2) too much, (3) too less, (4) way too less, (5) appropriate; (6) no opinion |  |  |  | 5 (IQR: 2-5) | 5 (IQR: 2-5) | 5 (IQR: 2-5) | 5 (IQR: 2-5) |
| Job resources | | | | | | | | | |
| Co-worker support | As colleagues we help each other (team) | (1) totally disagree; (2) disagree; (3) neutral; (4) agree; (5) totally agree; (6) not applicable | 4 (IQR: 4-5) |  | 4 (IQR: 4-5) |  |  |  |  |
|  | As colleagues we help each other (care chain) | (1) totally disagree; (2) disagree; (3) neutral; (4) agree; (5) totally agree; (6) not applicable | 4 (IQR: 3-4) |  | 4 (IQR: 3-4) |  |  |  |  |
|  | I receive help with my work when needed | (1) never; (2) sometimes; (3) regularly; (4) often; (5) always; (6) I don’t know |  | 4 (IQR: 3-4) |  |  |  |  |  |
|  | If I am having a hard time at work I can turn to someone | (1) never; (2) sometimes; (3) regularly; (4) often; (5) always; (6) I don’t know |  | 4 (IQR: 3-5) |  |  |  |  |  |
| Job control | I can decide how and when to do my work within reasonable limits | (1) totally disagree; (2) disagree; (3) neutral; (4) agree; (5) totally agree; (6) not applicable | 4 (IQR: 3-4) |  | 4 (IQR: 3-4) |  |  |  |  |
|  | I can decide how i do my work | (1) never; (2) sometimes; (3) regularly; (4) often; (5) always; (6) I don’t know |  | 4 (IQR: 3-4) |  |  |  |  |  |
|  | I can decide when i do my work | (1) never; (2) sometimes; (3) regularly; (4) often; (5) always; (6) I don’t know |  | 2 (IQR: 2-4) |  |  |  |  |  |
|  | I can set my own work pace | (1) never; (2) sometimes; (3) regularly; (4) often; (5) always; (6) I don’t know |  | 3 (IQR: 2-4) |  |  |  |  |  |
|  | I can take breaks whenever I need it | (1) never; (2) sometimes; (3) regularly; (4) often; (5) always; (6) I don’t know |  | 3 (IQR: 2-4) |  |  |  |  |  |
| Organizational justice | I can address mistakes and unsafe situations without fear of negative consequences | (1) totally disagree; (2) disagree; (3) neutral; (4) agree; (5) totally agree; (6) no opinion |  |  |  | 4 (IQR: 4-4) | 4 (IQR: 4-4) | 4 (IQR: 4-4) | 4 (IQR: 4-4) |
| Participation in decision making | I feel free to question decisions or actions of persons with greater authority | (1) totally disagree; (2) disagree; (3) neutral; (4) agree; (5) totally agree; (6) no opinion |  |  |  | 4 (IQR: 3-4) | 4 (IQR: 3-4) | 4 (IQR: 3-4) | 4 (IQR: 3-4) |
| Performance feedback | I receive sufficient feedback on how I do my work | (1) totally disagree; (2) disagree; (3) neutral; (4) agree; (5) totally agree; (6) not applicable | 4 (IQR: 3-4) |  | 4 (IQR: 3-4) |  |  |  |  |
|  | Within our team it is common to give feedback | (1) totally disagree; (2) disagree; (3) neutral; (4) agree; (5) totally agree; (6) no opinion |  |  |  | 4 (IQR: 3-4) | 4 (IQR: 3-4) | 4 (IQR: 3-4) | 4 (IQR: 3-4) |
|  | The feedback of my direct supervisor helps me to improve my work | (1) totally disagree; (2) disagree; (3) neutral; (4) agree; (5) totally agree; (6) no opinion |  |  |  | 4 (IQR: 3-4) | 4 (IQR: 3-4) | 4 (IQR: 3-4) | 4 (IQR: 3-4) |
| Possibilities for learning and development | I am given the opportunity to develop | (1) totally disagree; (2) disagree; (3) neutral; (4) agree; (5) totally agree; (6) not applicable | 4 (IQR: 3-4) |  | 4 (IQR: 3-4) |  |  |  |  |
|  | I get opportunity to learn and develop knowledge and skills | (1) totally disagree; (2) disagree; (3) neutral; (4) agree; (5) totally agree; (6) I don’t know |  | 3 (IQR: 2-4) |  |  |  |  |  |
|  | I can develop in my work | (1) totally disagree; (2) disagree; (3) neutral; (4) agree; (5) totally agree; (6) no opinion |  |  |  | 4 (IQR: 3-4) | 4 (IQR: 3-4) | 4 (IQR: 3-4) | 4 (IQR: 3-4) |
|  | I can continuously improve in my work | (1) totally disagree; (2) disagree; (3) neutral; (4) agree; (5) totally agree; (6) no opinion |  |  |  | 4 (IQR: 3-4) | 4 (IQR: 3-4) | 4 (IQR: 3-4) | 4 (IQR: 3-4) |
|  | Within our team we learn from mistakes | (1) totally disagree; (2) disagree; (3) neutral; (4) agree; (5) totally agree; (6) no opinion |  |  |  | 4 (IQR: 3-4) | 4 (IQR: 3-4) | 4 (IQR: 3-4) | 4 (IQR: 3-4) |
| Recognition | With my work I am of added value | (1) totally disagree; (2) disagree; (3) neutral; (4) agree; (5) totally agree; (6) not applicable | 4 (IQR: 4-5) |  | 4 (IQR: 4-5) |  |  |  |  |
|  | I receive sufficient appreciation for my work | (1) totally disagree; (2) disagree; (3) neutral; (4) agree; (5) totally agree; (6) not applicable | 4 (IQR: 3-4) |  | 4 (IQR: 3-4) |  |  |  |  |
|  | I am appreciated at work | (1) never; (2) sometimes; (3) regularly; (4) often; (5) always; (6) I don’t know |  | 4 (IQR: 3-4) |  |  |  |  |  |
| Task variety | My work is sufficiently varied | (1) totally disagree; (2) disagree; (3) neutral; (4) agree; (5) totally agree; (6) not applicable | 4 (IQR: 4-5) |  | 4 (IQR: 4-5) |  |  |  |  |
| Team atmosphere | The relationship with my colleagues is good (team) | (1) totally disagree; (2) disagree; (3) neutral; (4) agree; (5) totally agree; (6) not applicable | 4 (IQR: 4-5) |  | 4 (IQR: 4-5) |  |  |  |  |
|  | The relationship with my colleagues is good (care chain) | (1) totally disagree; (2) disagree; (3) neutral; (4) agree; (5) totally agree; (6) not applicable | 4 (IQR: 4-4) |  | 4 (IQR: 4-4) |  |  |  |  |
| Team effectiveness | As colleagues we work together in a smart way (team) | (1) totally disagree; (2) disagree; (3) neutral; (4) agree; (5) totally agree; (6) not applicable | 4 (IQR: 3-4) |  | 4 (IQR: 3-4) |  |  |  |  |
|  | As colleagues we work together in a smart way (care chain) | (1) totally disagree; (2) disagree; (3) neutral; (4) agree; (5) totally agree; (6) not applicable | 3 (IQR: 3-4) |  | 3 (IQR: 3-4) |  |  |  |  |
|  | Within our team we adhere to the agreements we make with each other | (1) totally disagree; (2) disagree; (3) neutral; (4) agree; (5) totally agree; (6) no opinion |  |  |  | 4 (IQR: 3-4) | 4 (IQR: 3-4) | 4 (IQR: 3-4) | 4 (IQR: 3-4) |
|  | Within our team we openly share knowledge and information | (1) totally disagree; (2) disagree; (3) neutral; (4) agree; (5) totally agree; (6) no opinion |  |  |  | 4 (IQR: 4-4) | 4 (IQR: 4-4) | 4 (IQR: 4-4) | 4 (IQR: 4-4) |
|  | I know the goals of our team | ((1) totally disagree; (2) disagree; (3) neutral; (4) agree; (5) totally agree; (6) no opinion |  |  |  | 4 (IQR: 4-4) | 4 (IQR: 3-4) | 4 (IQR: 3-4) | 4 (IQR: 3-4) |
|  | Within our team we use the results of the employee survey to make improvements | (1) totally disagree; (2) disagree; (3) neutral; (4) agree; (5) totally agree; (6) no opinion |  |  |  | 4 (IQR: 4-4) | 4 (IQR: 4-4) | 4 (IQR: 4-4) | 4 (IQR: 4-4) |
|  | We cooperate well in our team | (1) totally disagree; (2) disagree; (3) neutral; (4) agree; (5) totally agree; (6) no opinion |  |  |  | 4 (IQR: 4-4) | 4 (IQR: 4-4) | 4 (IQR: 4-4) | 4 (IQR: 4-4) |
| Trust in leadership | My direct supervisor provides good leadership | (1) totally disagree; (2) disagree; (3) neutral; (4) agree; (5) totally agree; (6) no opinion |  |  |  | 4 (IQR: 3-4) | 4 (IQR: 3-4) | 4 (IQR: 3-4) | 4 (IQR: 3-4) |
|  | My manager provides good leadership | (1) totally disagree; (2) disagree; (3) neutral; (4) agree; (5) totally agree; (6) no opinion |  |  |  | 4 (IQR: 3-6) | 4 (IQR: 3-6) | 4 (IQR: 3-6) | 4 (IQR: 3-6) |
| Other | My work environment is pleasant | (1) totally disagree; (2) disagree; (3) neutral; (4) agree; (5) totally agree; (6) not applicable | 4 (IQR: 3-4) |  | 4 (IQR: 3-4) |  |  |  |  |
|  | We cooperate well in our division | (1) totally disagree; (2) disagree; (3) neutral; (4) agree; (5) totally agree; (6) no opinion |  |  |  | 4 (IQR: 3-4) | 4 (IQR: 3-4) | 4 (IQR: 3-4) | 4 (IQR: 3-4) |
|  | We cooperate well in our hospital | (1) totally disagree; (2) disagree; (3) neutral; (4) agree; (5) totally agree; (6) no opinion |  |  |  | 3 (IQR: 3-4) | 3 (IQR: 3-4) | 3 (IQR: 3-4) | 3 (IQR: 3-4) |
|  | We cooperate well with organizations outside of our hospital | (1) totally disagree; (2) disagree; (3) neutral; (4) agree; (5) totally agree; (6) no opinion |  |  |  | 4 (IQR: 3-4) | 4 (IQR: 3-4) | 4 (IQR: 3-4) | 4 (IQR: 3-4) |
| Engaged leadership | | | | | | | | | |
| Connecting | The relationship with my direct supervisor is good | (1) totally disagree; (2) disagree; (3) neutral; (4) agree; (5) totally agree; (6) not applicable | 4 (IQR: 4-5) |  | 4 (IQR: 4-5) |  |  |  |  |
| Inspiring | My direct supervisor know how to motivate me | (1) totally disagree; (2) disagree; (3) neutral; (4) agree; (5) totally agree; (6) not applicable | 4 (IQR: 3-4) |  | 3 (IQR: 3-4) |  |  |  |  |
|  | My direct supervisor shows exemplary behavior | (1) totally disagree; (2) disagree; (3) neutral; (4) agree; (5) totally agree; (6) no opinion |  |  |  | 4 (IQR: 3-4) | 4 (IQR: 3-4) | 4 (IQR: 3-4) | 4 (IQR: 3-4) |
| Personal resources | | | | | | | | | |
| Self-efficacy | I can effectively solve problems in my work | (1) totally disagree; (2) disagree; (3) neutral; (4) agree; (5) totally agree; (6) no opinion |  |  |  | 4 (IQR: 3-4) | 4 (IQR: 3-4) | 4 (IQR: 3-4) | 4 (IQR: 3-4) |
| Goal directedness | I know what I need to do to achieve our team's goals | (1) totally disagree; (2) disagree; (3) neutral; (4) agree; (5) totally agree; (6) no opinion |  |  |  | 4 (IQR: 3-4) | 4 (IQR: 3-4) | 4 (IQR: 3-4) | 4 (IQR: 3-4) |
| Employee well-being | | | | | | | | | |
| Boredom | My work is challenging in a good way | 2020+2022: totally disagree; (2) disagree; (3) neutral; (4) agree; (5) totally agree; (6) not applicable  2021: (1) never; (2) sometimes; (3) regularly; (4) often; (5) always; (6) I don’t know | 4 (IQR: 4-4) | 3 (IQR: 3-4) | 4 (IQR: 4-4) |  |  |  |  |
| Burnout | Indicate where you are on the range of fatigue to vitality | 1-10 ((1) fatigue; (10) vital) |  | MEAN 6,50 | MEAN 6.4 |  |  |  |  |
| Job satisfaction | I enjoy my work | Hospital A 2020+2022: totally disagree; (2) disagree; (3) neutral; (4) agree; (5) totally agree; (6) not applicable  Hospital B: (1) totally disagree; (2) disagree; (3) neutral; (4) agree; (5) totally agree; (6) not applicable; (6) no opinion | 4 (IQR: 4-5) |  | 4 (IQR: 4-4) | 4 (IQR: 4-5) | 4 (IQR: 4-5) | 4 (IQR: 4-5) | 4 (IQR: 4-5) |
| Work engagement | Indicate where you are on the balance of disengaged to engaged | 1-10 ((1) disengagement; (10) engagement) |  | MEAN 7.14 | MEAN 6.94 |  |  |  |  |
| Other | I feel safe at work | (1) totally disagree; (2) disagree; (3) neutral; (4) agree; (5) totally agree; (6) no opinion |  |  |  | 4 (IQR: 4-5) | 4 (IQR: 4-5) | 4 (IQR: 4-5) | 4 (IQR: 4-5) |
| Outcomes | | | | | | | | | |
| Commitment organization | Working for this hospital makes me proud | (1) totally disagree; (2) disagree; (3) neutral; (4) agree; (5) totally agree; (6) not applicable | 4 (IQR: 3-4) |  | 4 (IQR: 3-4) |  |  |  |  |
|  | I like to do something extra for my work | ((1) totally disagree; (2) disagree; (3) neutral; (4) agree; (5) totally agree; (6) no opinion |  |  |  | 4 (IQR: 3-4) | 4 (IQR: 3-4) | 4 (IQR: 3-4) | 4 (IQR: 3-4) |
|  | The success of my hospital means much to me | (1) totally disagree; (2) disagree; (3) neutral; (4) agree; (5) totally agree; (6) no opinion |  |  |  | 4 (IQR: 3-4) | 4 (IQR: 3-4) | 4 (IQR: 3-4) | 4 (IQR: 3-4) |
|  | I rate working in this hospital as.. | 1-10 ((1) bad; (2) good) |  |  |  | MEAN 7.31 | MEAN 7.29 | MEAN 7.32 | MEAN 7.31 |
| Commitment team | I put my team's results above my personal ambitions | (1) totally disagree; (2) disagree; (3) neutral; (4) agree; (5) totally agree; (6) no opinion |  |  |  | 4 (IQR: 3-4) | 4 (IQR: 3-4) | 4 (IQR: 3-4) | 4 (IQR: 3-4) |
| Work ability | I can do my job without negative effects on my health | (1) totally disagree; (2) disagree; (3) neutral; (4) agree; (5) totally agree; (6) not applicable | 4 (IQR: 3-4) |  | 4 (IQR: 3-4) |  |  |  |  |
