# Supplementary material for: Uncovering gaps in workforce well-being: a national look at survey practice in Dutch university medical centres – an exploratory quantitative study
Source: BMJ Open. 2025 Jul 18;15(7):e094939. doi: 10.1136/bmjopen-2024-094939 (PMC12273149; doi:10.1136/bmjopen-2024-094939)
Supplement: online supplemental file 3 [file bmjopen-15-7-s003.docx]

**Additional file 3**

*Table F Comprehensive overview of results, including a distribution for all answer options per surveyed question.*

| **Sub-category** | **Question-item** | **Scale** | **Hospital A** | **Hospital A** | **Hospital A** | **Hospital B** | **Hospital B** | **Hospital B** | **Hospital B** |
| --- | --- | --- | --- | --- | --- | --- | --- | --- | --- |
|  |  |  | *Sept 2020 (4157)* | *Dec 2021 (4102)* | *July 2022 (3603)* | *Sept 2020 (5056)* | *Jan 2022 (4842)* | *May 2022 (4894)* | *May 2023 (4895)* |
| Job demands | | | | | | | | | |
| Work overload | There is an acceptable workload | (1) totally disagree; (2) disagree; (3) neutral; (4) agree; (5) totally agree; (6) not applicable | 4 (IQR: 3-4)  1: 2,36  2: 15,25  3: 22,40  **4: 52,51**  5: 7,43  6: 0,05 |  | 4 (IQR: 3-4)  1: 4,44  2: 19,15  3: 23,70  **4: 45,38**  5: 7,27  6: 0,06 |  |  |  |  |
|  | I have too much work | (1) never; (2) sometimes; (3) regularly; (4) often; (5) always; (6) I don’t know |  | 4 (IQR: 3-4)  1: 7,17  2: 15,19  3: 23,28  **4: 45,83**  5: 8,09  6: 0,44 |  |  |  |  |  |
|  | I think my workload is on an average base.. | (1) way too much, (2) too much, (3) too less, (4) way too less, (5) appropriate; (6) no opinion |  |  |  | 5 (IQR: 2-5)  1: 5,91  2: 29,98  3: 3,38  4: 0,44  **5: 58,15**  6: 2,14 | 5 (IQR: 2-5)  1: 5,68  2: 29,74  3: 3,10  4: 0,29  **5: 58,72**  6: 2,48 | 5 (IQR: 2-5)  1: 5,44  2: 30,49  3: 2,62  4: 0,39  **5: 59,11**  6: 1,96 | 5 (IQR: 2-5)  1: 5,54  2: 29,52  3: 2,80  4: 0,37  **5: 59,57**  6: 2,21 |
| Job resources | | | | | | | | | |
| Co-worker support | As colleagues we help each other (team) | (1) totally disagree; (2) disagree; (3) neutral; (4) agree; (5) totally agree; (6) not applicable | 4 (IQR: 4-5)  1: 0,22  2: 1,97  3: 9,65  **4: 54,44**  5: 33,41  6: 0,31 |  | 4 (IQR: 4-5)  1: 0,33  2: 3,08  3: 9,24  **4: 53,96**  5: 33,08  6: 0,31 |  |  |  |  |
|  | As colleagues we help each other (care chain) | (1) totally disagree; (2) disagree; (3) neutral; (4) agree; (5) totally agree; (6) not applicable | 4 (IQR: 3-4)  1: 0,55  2: 5,22  3: 23,91  **4: 56,03**  5: 10,39  6: 3,90 |  | 4 (IQR: 3-4)  1: 0,78  2: 8,16  3: 27,42  **4: 53,87**  5: 8,16  6: 1,61 |  |  |  |  |
|  | I receive help with my work when needed | (1) never; (2) sometimes; (3) regularly; (4) often; (5) always; (6) I don’t know |  | 4 (IQR: 3-4)  1: 1,27  2: 19,82  3: 26,04  **4: 36,13**  5: 16,38  6: 0,37 |  |  |  |  |  |
|  | If I am having a hard time at work I can turn to someone | (1) never; (2) sometimes; (3) regularly; (4) often; (5) always; (6) I don’t know |  | 4 (IQR: 3-5)  1: 2,02  2: 18,38  3: 18,31  **4: 29,96**  5: 27,47  6: 3,85 (3944) |  |  |  |  |  |
| Job control | I can decide how and when to do my work within reasonable limits | (1) totally disagree; (2) disagree; (3) neutral; (4) agree; (5) totally agree; (6) not applicable | 4 (IQR: 3-4)  1: 3,08  2: 13,69  3: 16,45  **4: 46,48**  5: 19,70  6: 0,60 |  | 4 (IQR: 3-4)  1: 3,72  2: 13,91  3: 16,24  **4: 45,74**  5: 19,87  6: 0,53 |  |  |  |  |
|  | I can decide how i do my work | (1) never; (2) sometimes; (3) regularly; (4) often; (5) always; (6) I don’t know |  | 4 (IQR: 3-4)  1: 4,10  2: 17,21  3: 22,60  **4: 44,61**  5: 11,14  6: 0,34 |  |  |  |  |  |
|  | I can decide when i do my work | (1) never; (2) sometimes; (3) regularly; (4) often; (5) always; (6) I don’t know |  | 2 (IQR: 2-4)  1: 18,70  2: 24,79  3: 19,97  **4: 29,62**  5: 6,46  6: 0,46 |  |  |  |  |  |
|  | I can set my own work pace | (1) never; (2) sometimes; (3) regularly; (4) often; (5) always; (6) I don’t know |  | 3 (IQR: 2-4)  1: 10,56  2: 29,64  3: 21,60  **4: 28,64**  5: 9,17  6: 0,39 |  |  |  |  |  |
|  | I can take breaks whenever I need it | (1) never; (2) sometimes; (3) regularly; (4) often; (5) always; (6) I don’t know |  | 3 (IQR: 2-4)  1: 10,53  **2: 32,91**  3: 24,01  4: 23,26  5: 9,04  6: 0,24 |  |  |  |  |  |
| Organizational justice | I can address mistakes and unsafe situations without fear of negative consequences | (1) totally disagree; (2) disagree; (3) neutral; (4) agree; (5) totally agree; (6) no opinion |  |  |  | 4 (IQR: 4-4)  1: 1,44  2: 5,66  3: 11,35  **4: 58,86**  5: 21,91  6: 0,77 | 4 (IQR: 4-4)  1: 1,38  2: 6,61  3: 12,06  **4: 57,58**  5: 21,66  6: 0,70 | 4 (IQR: 4-4)  1: 1,29  2: 5,72  3: 12,44  **4: 57,81**  5: 22,03  6: 0,72 | 4 (IQR: 4-4)  1: 1,47  2: 6,13  3: 11,44  **4: 58,16**  5: 22,33  6: 0,47 |
| Participation in decision making | I feel free to question decisions or actions of persons with greater authority | (1) totally disagree; (2) disagree; (3) neutral; (4) agree; (5) totally agree; (6) no opinion |  |  |  | 4 (IQR: 3-4)  1: 2,27  2: 8,72  3: 18,47  **4: 54,29**  5: 15,35  6: 0,89 | 4 (IQR: 3-4)  1: 1,92  2: 9,31  3: 18,81  **4: 54,42**  5: 14,64  6: 0,89 | 4 (IQR: 3-4)  1: 2,13  2: 8,91  3: 18,66  **4: 54,56**  5: 14,92  6: 0,84 | 4 (IQR: 3-4)  1: 2,10  2: 9,05  3: 17,53  **4: 54,95**  5: 15,53  6: 0,84 |
| Performance feedback | I receive sufficient feedback on how I do my work | (1) totally disagree; (2) disagree; (3) neutral; (4) agree; (5) totally agree; (6) not applicable | 4 (IQR: 3-4)  1: 2,74  2: 14,82  3: 28,48  **4: 41,78**  5: 11,28  6: 0,89 |  | 4 (IQR: 3-4)  1: 2,58  2: 16,49  3: 27,92  **4: 41,16**  5: 10,88  6: 0,97 |  |  |  |  |
|  | Within our team it is common to give feedback | (1) totally disagree; (2) disagree; (3) neutral; (4) agree; (5) totally agree; (6) no opinion |  |  |  | 4 (IQR: 3-4)  1: 2,06  2: 13,15  3: 23,95  **4: 49,47**  5: 10,01  6: 1,36 | 4 (IQR: 3-4)  1: 2,21  2: 12,43  3: 24,04  **4: 49,44**  5: 10,64  6: 1,24 | 4 (IQR: 3-4)  1: 2,02  2:12,77  3: 25,26  **4: 49,47**  5: 9,58  6: 0,90 | 4 (IQR: 3-4)  1: 1,82  2: 12,50  3: 24,37  **4: 50,93**  5: 9,66  6: 0,72 |
|  | The feedback of my direct supervisor helps me to improve my work | (1) totally disagree; (2) disagree; (3) neutral; (4) agree; (5) totally agree; (6) no opinion |  |  |  | 4 (IQR: 3-4)  1: 2,00  2: 6,65  3: 20,47  **4: 51,05**  5: 14,62  6: 5,22 | 4 (IQR: 3-4)  1: 2,52  2: 6,36  3: 18,88  **4: 51,80**  5: 14,95  6: 5,49 | 4 (IQR: 3-4)  1: 1,84  2: 5,76  3: 21,03  **4: 51,66**  5: 14,67  6: 5,05 | 4 (IQR: 3-4)  1: 1,96  2: 5,76  3: 18,86  **4: 51,77**  5: 16,14  6: 5,52 |
| Possibilities for learning and development | I am given the opportunity to develop | (1) totally disagree; (2) disagree; (3) neutral; (4) agree; (5) totally agree; (6) not applicable | 4 (IQR: 3-4)  1: 1,61  2: 8,18  3: 21,43  **4: 49,60**  5: 18,98  6: 0,19 |  | 4 (IQR: 3-4)  1: 2,28  2: 10,46  3: 22,04  **4: 47,68**  5: 17,24  6: 0,31 |  |  |  |  |
|  | I get opportunity to learn and develop knowledge and skills | (1) totally disagree; (2) disagree; (3) neutral; (4) agree; (5) totally agree; (6) I don’t know |  | 3 (IQR: 2-4)  1: 3,90  2: 24,26  3: 29,55  **4: 31,89**  5: 9,53  6: 0,88 |  |  |  |  |  |
|  | I can develop in my work | (1) totally disagree; (2) disagree; (3) neutral; (4) agree; (5) totally agree; (6) no opinion |  |  |  | 4 (IQR: 3-4)  1: 2,22  2: 9,30  3: 17,84  **4: 53,22**  5: 16,53  6: 0,89 | 4 (IQR: 3-4)  1: 1,90  2: 10,12  3: 18,11  **4: 52,38**  5: 16,67  6: 0,83 | 4 (IQR: 3-4)  1: 1,59  2: 9,62  3: 17,90  **4: 53,72**  5: 16,51  6: 0,65 | 4 (IQR: 3-4)  1: 2,35  2: 8,72  3: 17,92  **4: 54,03**  5: 16,04  6: 0,94 |
|  | I can continuously improve in my work | (1) totally disagree; (2) disagree; (3) neutral; (4) agree; (5) totally agree; (6) no opinion |  |  |  | 4 (IQR: 3-4)  1: 1,15  2: 6,49  3: 20,53  **4: 56,65**  5: 14,38  6: 0,81 | 4 (IQR: 3-4)  1: 0,85  2: 6,98  3: 20,40  **4: 56,63**  5: 14,11  6: 1,03 | 4 (IQR: 3-4)  1: 0,76  2: 6,50  3: 19,76  **4: 58,36**  5: 14,00  6: 0,63 | 4 (IQR: 3-4)  1: 1,21  2: 5,97  3: 20,98  **4: 57,14**  5: 13,97  6: 0,74 |
|  | Within our team we learn from mistakes | (1) totally disagree; (2) disagree; (3) neutral; (4) agree; (5) totally agree; (6) no opinion |  |  |  | 4 (IQR: 3-4)  1: 1,31  2: 7,38  3: 17,84  **4: 62,36**  5: 9,75  6: 1,36 | 4 (IQR: 3-4)  1: 1,22  2: 7,04  3: 18,01  **4: 61,61**  5: 10,70  6: 1,43 | 4 (IQR: 3-4)  1: 0,92  2: 7,81  3: 17,12  **4: 62,34**  5: 10,63  6: 1,19 | 4 (IQR: 3-4)  1: 1,39  2: 7,09  3: 17,73  **4: 63,49**  5: 9,38  6: 0,92 |
| Recognition | With my work I am of added value | (1) totally disagree; (2) disagree; (3) neutral; (4) agree; (5) totally agree; (6) not applicable | 4 (IQR: 4-5)  1: 0,12  2: 1,37  3: 9,26  **4: 59,97**  5: 29,20  6: 0,07 |  | 4 (IQR: 4-5)  1: 0,31  2: 1,55  3: 11,43  **4: 60,78**  5: 25,92  6: 0,00 |  |  |  |  |
|  | I receive sufficient appreciation for my work | (1) totally disagree; (2) disagree; (3) neutral; (4) agree; (5) totally agree; (6) not applicable | 4 (IQR: 3-4)  1: 3,01  2: 12,00  3: 27,93  **4: 42,80**  5: 13,69  6: 0,58 |  | 4 (IQR: 3-4)  1: 4,30  2: 14,68  3: 27,03  **4: 40,36**  5: 13,18  6: 0,44 |  |  |  |  |
|  | I am appreciated at work | (1) never; (2) sometimes; (3) regularly; (4) often; (5) always; (6) I don’t know |  | 4 (IQR: 3-4)  1: 0,93  2: 18,82  3: 29,47  **4: 37,06**  5: 11,70  6: 2,02 |  |  |  |  |  |
| Task variety | My work is sufficiently varied | (1) totally disagree; (2) disagree; (3) neutral; (4) agree; (5) totally agree; (6) not applicable | 4 (IQR: 4-5)  1: 0,34  2: 3,51  3: 10,03  **4: 50,28**  5: 35,75  6: 0,10 |  | 4 (IQR: 4-5)  1: 0,67  2: 3,05  3: 10,10  **4: 54,82**  5: 31,28  6: 0,08 |  |  |  |  |
| Team atmosphere | The relation with my colleagues is good (team) | (1) totally disagree; (2) disagree; (3) neutral; (4) agree; (5) totally agree; (6) not applicable | 4 (IQR: 4-5)  1: 0,07  2: 0,91  3: 7,67  **4: 57,73**  5: 33,41  6: 0,19 |  | 4 (IQR: 4-5)  1: 0,08  2: 1,61  3: 8,66  **4: 57,09**  5: 32,33  6: 0,22 |  |  |  |  |
|  | The relation with my colleagues is good (care chain) | (1) totally disagree; (2) disagree; (3) neutral; (4) agree; (5) totally agree; (6) not applicable | 4 (IQR: 4-4)  1: 0,17  2: 1,61  3: 19,34  **4: 62,69**  5: 12,44  6: 3,75 |  | 4 (IQR: 4-4)  1: 0,25  2: 1,94  3: 21,18  **4: 64,61**  5: 10,46  6: 1,55 |  |  |  |  |
| Team effectiveness | As colleagues we work together in a smart way (team) | (1) totally disagree; (2) disagree; (3) neutral; (4) agree; (5) totally agree; (6) not applicable | 4 (IQR: 3-4)  1: 0,63  2: 10,44  3: 28,27  **4: 47,58**  5: 12,51  6: 0,58 |  | 4 (IQR: 3-4)  1: 1,14  2: 10,91  3: 28,86  **4: 47,54**  5: 11,05  6: 0,50 |  |  |  |  |
|  | As colleagues we work together in a smart way (care chain) | (1) totally disagree; (2) disagree; (3) neutral; (4) agree; (5) totally agree; (6) not applicable | 3 (IQR: 3-4)  1: 1,61  2: 14,31  **3: 39,93**  4: 35,05  5: 5,03  6: 4,07 |  | 3 (IQR: 3-4)  1: 2,78  2: 17,85  **3: 43,05**  4: 30,92  5: 3,61  6: 1,80 (3538) |  |  |  |  |
|  | Within our team we adhere to the agreements we make with each other | (1) totally disagree; (2) disagree; (3) neutral; (4) agree; (5) totally agree; (6) no opinion |  |  |  | 4 (IQR: 3-4)  1: 1,36  2: 10,19  3: 22,41  **4: 57,16**  5: 7,83  6: 1,05 | 4 (IQR: 3-4)  1: 1,49  2: 9,00  3: 21,87  **4: 57,83**  5: 8,49  6: 1,32 | 4 (IQR: 3-4)  1: 1,25  2: 9,91  3: 22,91  **4: 57,05**  5: 7,85  6: 1,04 | 4 (IQR: 3-4)  1: 1,55  2: 9,48  3: 22,90  **4: 57,77**  5: 7,44  6: 0,86 |
|  | Within our team we openly share knowledge and information | (1) totally disagree; (2) disagree; (3) neutral; (4) agree; (5) totally agree; (6) no opinion |  |  |  | 4 (IQR: 4-4)  1: 1,19  2: 5,18  3: 13,67  **4: 60,36**  5: 18,79  6: 0,81 | 4 (IQR: 4-4)  1: 0,95  2: 5,43  3: 13,18  **4: 59,67**  5: 19,97  6: 0,81 | 4 (IQR: 4-4)  1: 0,86  2: 5,66  3: 13,79  **4: 60,05**  5: 19,17  6: 0,47 | 4 (IQR: 4-4)  1: 1,04  2: 4,96  3: 13,22  **4: 61,29**  5: 18,96  6: 0,53 |
|  | I know the goals of our team | ((1) totally disagree; (2) disagree; (3) neutral; (4) agree; (5) totally agree; (6) no opinion |  |  |  | 4 (IQR: 4-4)  1: 1,27  2: 6,96  3: 16,36  **4: 62,32**  5: 11,43  6: 1,66 | 4 (IQR: 3-4)  1 : 1,12  2: 7,02  3: 17,02  **4: 62,06**  5: 10,90  6: 1,88 | 4 (IQR: 3-4)  1: 1,10  2: 7,52  3: 17,14  **4: 62,06**  5: 10,58  6: 1,59 | 4 (IQR: 3-4)  1: 1,27  2: 6,97  3: 16,81  **4: 62,80**  5: 10,38  6: 1,78 |
|  | Within our team we use the results of the employee survey to make improvements | (1) totally disagree; (2) disagree; (3) neutral; (4) agree; (5) totally agree; (6) no opinion |  |  |  | 4 (IQR: 4-4)  1: 0,59  2: 3,20  3: 14,79  **4: 59,24**  5: 21,12  6: 1,05 | 4 (IQR: 4-4)  1: 0,95  2: 2,91  3: 15,99  **4: 57,79**  5: 21,50  6: 0,87 | 4 (IQR: 4-4)  1: 0,57  2: 3,04  3: 15,04  **4: 58,52**  5: 21,84  6: 0,98 | 4 (IQR: 4-4)  1: 1,00  2: 2,74  3: 16,10  **4: 57,81**  5: 21,49  6: 0,86 |
|  | We cooperate well in our team | (1) totally disagree; (2) disagree; (3) neutral; (4) agree; (5) totally agree; (6) no opinion |  |  |  | 4 (IQR: 4-4)  1: 1,01  2: 5,60  3: 13,41  **4: 61,79**  5: 17,42  6: 0,77 | 4 (IQR: 4-4)  1: 1,01  2: 5,93  3: 13,32  **4: 61,30**  5: 17,49  6: 0,95 | 4 (IQR: 4-4)  1: 1,00  2: 5,58  3: 13,22  **4: 62,38**  5: 17,02  6: 0,80 | 4 (IQR: 4-4)  1: 0,88  2: 5,37  3: 13,63  **4: 61,96**  5: 17,43  6: 0,74 |
| Trust in leadership | My direct supervisor provides good leadership | (1) totally disagree; (2) disagree; (3) neutral; (4) agree; (5) totally agree; (6) no opinion |  |  |  | 4 (IQR: 3-4)  1: 2,00  2: 7,71  3: 19,84  **4: 47,73**  5: 18,37  6: 4,35 | 4 (IQR: 3-4)  1: 2,60  2: 6,59  3: 19,62  **4: 49,17**  5: 18,48  6: 3,53 | 4 (IQR: 3-4)  1: 2,15  2: 6,74  3: 19,49  **4: 48,98**  5: 18,76  6: 3,88 | 4 (IQR: 3-4)  1: 2,29  2: 6,29  3: 17,63  **4: 50,07**  5: 20,06  6: 3,66 |
|  | My manager provides good leadership | (1) totally disagree; (2) disagree; (3) neutral; (4) agree; (5) totally agree; (6) no opinion |  |  |  | 4 (IQR: 3-6)  1: 2,35  2: 5,70  3: 24,35  **4: 30,58**  5: 7,30  6: 29,73 (3553) | 4 (IQR: 3-6)  1: 2,75  2: 5,80  3: 23,11  **4: 31,06**  5: 7,70  6: 29,57 | 4 (IQR: 3-6)  1: 2,31  2: 6,01  3: 24,42  **4: 29,79**  5: 7,27  6: 30,20 | 4 (IQR: 3-6)  1: 2,53  2: 5,64  3: 24,27  **4: 30,17**  5: 7,74  6: 29,64 |
| Other | My work environment is pleasant | (1) totally disagree; (2) disagree; (3) neutral; (4) agree; (5) totally agree; (6) not applicable | 4 (IQR: 3-4)  1: 1,03  2: 6,47  3: 19,36  **4: 57,18**  5: 15,83  6: 0,12 |  | 4 (IQR: 3-4)  1: 1,64  2: 8,66  3: 19,65  **4: 55,68**  5: 14,35  6: 0,03 |  |  |  |  |
|  | We cooperate well in our division | (1) totally disagree; (2) disagree; (3) neutral; (4) agree; (5) totally agree; (6) no opinion |  |  |  | 4 (IQR: 3-4)  1: 2,35  2: 12,08  3: 31,61  **4: 42,92**  5: 4,19  6: 6,84 | 4 (IQR: 3-4)  1: 2,40  2: 12,80  3: 31,52  **4: 41,14**  5: 4,75  6: 7,39 | 4 (IQR: 3-4)  1: 2,10  2: 12,83  3: 32,35  **4: 42,50**  5: 3,60  6: 6,62 | 4 (IQR: 3-4)  1: 2,23  2: 12,54  3: 32,52  **4: 42,25**  5: 3,74  6: 6,72 |
|  | We cooperate well in our hospital | (1) totally disagree; (2) disagree; (3) neutral; (4) agree; (5) totally agree; (6) no opinion |  |  |  | 3 (IQR: 3-4)  1: 2,79  2: 12,62  **3: 37,01**  4: 34,77  5: 3,03  6: 9,79 | 3 (IQR: 3-4)  1: 2,75  2: 12,99  **3: 37,86**  4: 33,89  5: 2,89  6: 9,62 | 3 (IQR: 3-4)  1: 2,27  2: 13,02  **3: 39,01**  4: 34,43  5: 2,31  6: 8,97 | 3 (IQR: 3-4)  1: 2,49  2: 13,14  **3: 38,57**  4: 34,18  5: 2,70  6: 8,93 |
|  | We cooperate well with organizations outside of our hospital | (1) totally disagree; (2) disagree; (3) neutral; (4) agree; (5) totally agree; (6) no opinion |  |  |  | 4 (IQR: 3-4)  1: 1,21  2: 6,45  3: 30,95  **4: 39,32**  5: 4,15  6: 17,92 | 4 (IQR: 3-4)  1: 1,32  2: 5,10  3: 30,42  **4: 39,20**  5: 4,58  6: 19,37 | 4 (IQR: 3-4)  1: 1,02  2: 5,93  3: 32,49  **4: 37,45**  5: 3,78  6: 19,33 | 4 (IQR: 3-4)  1: 1,37  2: 5,92  3: 31,64  **4: 38,63**  5: 4,49  6: 17,94 |
| Engaged leadership | | | | | | | | | |
| Connecting | The relationship with my direct supervisor is good | (1) totally disagree; (2) disagree; (3) neutral; (4) agree; (5) totally agree; (6) not applicable | 4 (IQR: 4-5)  1: 0,82  2: 3,44  3: 14,10  **4: 52,92**  5: 27,93  6: 0,79 |  | 4 (IQR: 4-5)  1: 1,36  2: 3,47  3: 15,29  **4: 51,18**  5: 27,89  6: 0,80 |  |  |  |  |
| Inspiring | My direct supervisor know how to motivate me | (1) totally disagree; (2) disagree; (3) neutral; (4) agree; (5) totally agree; (6) not applicable | 4 (IQR: 3-4)  1: 2,74  2: 12,29  3: 33,97  **4: 38,37**  5: 11,21  6: 1,42 |  | 3 (IQR: 3-4)  1: 3,91  2: 13,18  3: 33,08  **4: 36,77**  5: 11,63  6: 1,42 |  |  |  |  |
|  | My direct supervisor shows exemplary behavior | (1) totally disagree; (2) disagree; (3) neutral; (4) agree; (5) totally agree; (6) no opinion |  |  |  | 4 (IQR: 3-4)  1: 2,41  2: 7,69  3: 22,27  **4: 46,52**  5: 16,06  6: 5,04 | 4 (IQR: 3-4)  1: 2,54  2: 7,46  3: 21,19  **4: 47,56**  5: 16,40  6: 4,85 | 4 (IQR: 3-4)  1: 2,25  2: 7,27  3: 21,76  **4: 47,45**  5: 16,88  6: 4,39 | 4 (IQR: 3-4)  1: 2,45  2: 6,84  3: 20,47  **4: 48,40**  5: 17,71  6: 4,13 |
| Personal resources | | | | | | | | | |
| Self-efficacy | I can effectively solve problems in my work | (1) totally disagree; (2) disagree; (3) neutral; (4) agree; (5) totally agree; (6) no opinion |  |  |  | 4 (IQR: 3-4)  1: 1,44  2: 8,60  3: 22,69  **4: 56,53**  5: 9,69  6: 1,05 | 4 (IQR: 3-4)  1: 1,63  2: 8,80  3: 23,07  **4: 56,69**  5: 8,96  6: 0,85 | 4 (IQR: 3-4)  1: 1,45  2: 8,62  3: 23,15  **4: 57,15**  5: 8,87  6: 0,76 | 4 (IQR: 3-4)  1: 1,43  2: 8,27  3: 23,31  **4: 57,88**  5: 8,38  6: 0,74 |
| Goal directedness | I know what to do to achieve the aims within our team | (1) totally disagree; (2) disagree; (3) neutral; (4) agree; (5) totally agree; (6) no opinion |  |  |  | 4 (IQR: 3-4)  1: 1,42  2: 6,98  3: 19,13  **4: 60,15**  5: 9,91  6: 2,41 | 4 (IQR: 3-4)  1: 1,01  2 :7,19  3: 19,08  **4: 60,43**  5: 9,54  6: 2,75 | 4 (IQR: 3-4)  1: 1,00  2: 7,32  3: 19,51  **4: 59,93**  5: 9,91  6: 2,33 | 4 (IQR: 3-4)  1: 1,27  2: 6,72  3: 19,51  **4: 60,67**  5: 9,44  6: 2,39 |
| Employee well-being | | | | | | | | | |
| Boredom | My work is challenging in a good way | 2021+2023: totally disagree; (2) disagree; (3) neutral; (4) agree; (5) totally agree; (6) not applicable  2021: (1) never; (2) sometimes; (3) regularly; (4) often; (5) always; (6) I don’t know | 4 (IQR: 4-4)  1: 0,41  2: 4,86  3: 15,28  **4: 58,50**  5: 20,95  6: 0,00 | 3 (IQR: 3-4)  1: 1,71  2: 18,26  3: 31,23  **4: 39,54**  5: 9,07  6: 0,20 | 4 (IQR: 4-4)  1: 0,86  2: 5,91  3: 17,21  **4: 57,48**  5: 18,54  6: 0,00 |  |  |  |  |
| Burnout | Rate yourself on a scale of exhausted and vital | 1-10 ((1) exhausted; (2) vital) |  | MEAN 6,50 | MEAN 6.4 |  |  |  |  |
| Job satisfaction | I experience joy I my work | Hospital A 2020+2022: totally disagree; (2) disagree; (3) neutral; (4) agree; (5) totally agree; (6) not applicable  Hospital B: (1) totally disagree; (2) disagree; (3) neutral; (4) agree; (5) totally agree; (6) not applicable; (6) no opinion | 4 (IQR: 4-5)  1: 0,22  2: 2,77  3: 14,07  **4: 56,75**  5: 26,20  6: 0,00 |  | 4 (IQR: 4-4)  1: 0,56  2: 4,02  3: 16,99  **4: 57,59**  5: 20,84  6: 0,00 | 4 (IQR: 4-5)  1: 0,69  2: 4,67  3: 11,10  **4: 55,52**  5: 27,79  6: 0,24 | 4 (IQR: 4-5)  1: 0,95  2: 4,73  3: 10,80  **4: 55,76**  5: 27,49  6: 0,27 | 4 (IQR: 4-5)  1: 0,55  2: 3,66  3: 11,69  **4: 55,88**  5: 28,03  6: 0,18 | 4 (IQR: 4-5)  1: 0,78  2: 4,31  3: 11,15  **4: 57,24**  5: 26,35  6: 0,16 |
| Work engagement | Indicate where you are on the range of fatigue to vitality | 1-10 ((1) disengagement; (2) engagement) |  | MEAN 7.14 | MEAN 6.94 |  |  |  |  |
| Other | I feel safe at work | (1) totally disagree; (2) disagree; (3) neutral; (4) agree; (5) totally agree; (6) no opinion |  |  |  | 4 (IQR: 4-5)  1: 1,50  2: 5,70  3: 10,48  **4: 51,17**  5: 30,83  6: 0,32 | 4 (IQR: 4-5)  1: 1,88  2: 5,60  3: 11,09  **4: 50,23**  5: 30,94  6: 0,27 | 4 (IQR: 4-5)  1: 1,12  2: 5,62  3: 11,14  **4: 51,76**  5: 30,14  6: 0,22 | 4 (IQR: 4-5)  1: 1,59  2: 5,62  3: 10,09  **4: 50,87**  5: 31,54  6: 0,29 |
| Outcomes | | | | | | | | | |
| Commitment - organization | Working for this hospital makes me proud | (1) totally disagree; (2) disagree; (3) neutral; (4) agree; (5) totally agree; (6) not applicable | 4 (IQR: 3-4)  1: 0,55  2: 3,90  3: 26,99  **4: 52,61**  5: 15,73  6: 0,22 |  | 4 (IQR: 3-4)  1: 1,11  2: 7,02  3: 33,28  **4: 47,07**  5: 11,07  6: 0,44 |  |  |  |  |
|  | I like to do something extra for my work | ((1) totally disagree; (2) disagree; (3) neutral; (4) agree; (5) totally agree; (6) no opinion |  |  |  | 4 (IQR: 3-4)  1: 0,91  2: 7,63  3: 27,83  **4: 50,63**  5: 9,38  6: 3,62 | 4 (IQR: 3-4)  1: 1,05  2: 7,70  3: 28,73  **4: 49,40**  5: 9,71  6: 3,41 | 4 (IQR: 3-4)  1: 0,57  2: 7,72  3: 29,44  **4: 49,57**  5: 9,42  6: 3,27 | 4 (IQR: 3-4)  1: 1,25  2: 6,93  3: 29,42  **4: 50,13**  5: 9,15  6: 3,13 |
|  | The success of my hospital means much to me | (1) totally disagree; (2) disagree; (3) neutral; (4) agree; (5) totally agree; (6) no opinion |  |  |  | 4 (IQR: 3-4)  1: 1,62  2: 6,86  3: 30,48  **4: 48,44**  5: 9,34  6: 3,26 | 4 (IQR: 3-4)  1: 1,59  2: 6,92  3: 30,63  **4: 48,04**  5: 10,06  6: 2,77 | 4 (IQR: 3-4)  1: 1,55  2: 6,97  3: 30,85  **4: 48,16**  5: 9,30  6: 3,17 | 4 (IQR: 3-4)  1: 1,86  2: 6,62  3: 31,09  **4: 48,80**  5: 8,93  6: 2,70 |
|  | I rate working in this hospital as.. | 1-10 ((1) bad; (2) good) |  |  |  | MEAN 7.31 | MEAN 7.29 | MEAN 7.32 | MEAN 7.31 |
| Commitment - team | I put my team's results above my personal ambitions | (1) totally disagree; (2) disagree; (3) neutral; (4) agree; (5) totally agree; (6) no opinion |  |  |  | 4 (IQR: 3-4)  1: 7,38  2: 14,10  3: 23,81  **4: 36,97**  5: 6,37  6: 11,37 | 4 (IQR: 3-4)  1: 7,25  2: 14,25  3: 23,67  **4: 36,16**  5: 7,31  6: 11,36 | 4 (IQR: 3-4)  1: 6,64  2: 14,04  3: 24,91  **4: 36,78**  5: 6,74  6: 10,89 | 4 (IQR: 3-4)  1: 7,40  2: 15,55  3: 22,59  **4: 36,85**  5: 7,05  6: 10,56 |
| Work ability | I can do my job without negative effects on my health | (1) totally disagree; (2) disagree; (3) neutral; (4) agree; (5) totally agree; (6) not applicable | 4 (IQR: 3-4)  1: 1,59  2: 16,29  3: 24,06  **4: 42,19**  5: 15,85  6: 0,02 |  | 4 (IQR: 3-4)  1: 3,08  2: 19,40  3: 25,40  **4: 39,99**  5: 12,10  6: 0,03 |  |  |  |  |
