# Supplementary material for: Uncovering gaps in workforce well-being: a national look at survey practice in Dutch university medical centres – an exploratory quantitative study
Source: BMJ Open. 2025 Jul 18;15(7):e094939. doi: 10.1136/bmjopen-2024-094939 (PMC12273149; doi:10.1136/bmjopen-2024-094939)
Supplement: online supplemental file 4 [file bmjopen-15-7-s004.docx]

**Additional file 4**

**Job demands**Work overload
Workload was measured by both hospitals with three different questions. Within the hospitals, workload barely differed over time. Hospital A shows a median of 4 (IQR: 3-4), indicating on one hand an acceptable workload and on the other hand often too much work. Hospital B shows a median of 5 (IQR: 2-5), indicating an appropriate workload with a range of 3. However, answer options were as follows: (1) way too much, (2) too much, (3) too less, (4) way too less, (5) appropriate; (6) no opinion.

**Job resources**Co-worker support
Co-worker support was measured by Hospital A with four questions and experiences barely seem to differ over time. Hospital A shows a median of 4 for all questions, indicating agreement with the questions about helping each other as colleagues. The item about helping each other within teams shows an IQR of 4-5 and helping each other within the care chain shows an IQR of 3-4, indicating better co-worker support within teams compared to co-worker support within the care chain. The fourth question about going to someone when its hard shows an IQR of 3-5, indicating a more spread experience about this item. Additionally, in 2021, respondents from hospital A most frequently answered this question with 'I don't know’.

Job-control
Aspects of job control were measured by Hospital A using five questions. Experiences barely differed over time. Hospital A shows variation in median and IQR. Questions about determining own timing of work, work tempo and taking breaks show the greatest variation in responses with a range of 2, indicating that opinions and experiences on this topic differ. One item, "I can determine myself when I do my work," measured in December 2021, had a median score of 2, suggesting room for improvement. The IQR was 2-4, indicating varying responses. Interestingly, the most frequently selected answer was ‘often’, chosen by 29.62% of respondents. Two related items, "I can set my own work pace" and "I can take breaks whenever I need it," both had a median score of 3 (IQR: 2-4). For the work tempo item, 28.64% chose 4 (often), while for the breaks item, 32.91% chose 2 (sometimes), indicating more variability and potential for improvement.

Organizational justice
Organizational justice was measures by hospital B with one question and experiences barely differed over time. Hospital B shows a median of 4 (IQR: 4-4) at all measurement moments, indicating small ranges and agreement about this item.

Participation in decision making
Participation in decision making was measured by hospital B with one question and experiences barely differed over time. Hospital B shows a median of 4 (IQR: 3-4) at all measurement moments.

Performance feedback
Performance feedback was measured by both hospitals with three different questions and experiences barely differed over time. At all measurements moments of both hospitals a median of 4 (IQR: 3-4) was discovered.

Possibilities for learning and development
Possibilities for learning and development was measured by both hospitals with five different questions and experiences barely differed over time. Except for one question, at all measurements moments of both hospitals a median of 4 (IQR: 3-4) was discovered. The question ‘I get the chance to learn and develop my knowledge and skills’ of Hospital A in 2021 shows a median of 3 (IQR: 2-4), indicating greater variation in response and thus in opinions and experiences with a range of 2. The most common response was 4 (agree), chosen by 31.89% of respondents, suggesting a relatively high frequency of agreement but also indicating variability and room for growth.

Recognition
Recognition was measured by hospital A with three questions and experiences barely differed over time. Medians for all questions and measurement moments was 4. The IQR varied from 3-4 to 4-5, indicating more agreement about adding value through one's own perspectives compared to receiving recognition and feeling recognized by others.

Task variety
Task variety was measured by hospital A with one question and experiences barely differed over time. Both measurement moments show a median of 4 (IQR: 4-5), indicating mainly agreement with a sufficient task variety.

Team atmosphere
Team atmosphere was measured by hospital A with two question and experiences barely differed over time. Both measurement moments and questions show a median of 4, but vary in IQR from 4-4 to 4-5, indicating more unanimity regarding the agreement on having a good relationship with colleagues within the chain. There is greater variability concerning the relationship within the team, with more respondents choosing "totally agree" compared to the question about the relationship within the chain.

Team effectiveness
Team effectiveness was measured by both hospitals with seven questions and experiences barely differed over time. Except for one question measured by hospital A, for all questions and measurement moments a median of 4 discovered. That question ‘as colleagues we work together in a smart way (care chain)’ shows a median of 3, indicating more experienced work effectiveness within own teams than within the care chain. Moreover, respondents from hospital A in 2020 and 2022 most frequently answered this question with 'not applicable’. Furthermore, two questions measured by hospital B about ‘we use the results of the employee survey’ and ‘we work effectively together’ show IQRs of 4-4, indicating less variability and more unanimity regarding agreement about this statements.

Trust in leadership
Trust in leadership was measured by hospital B with two questions and experiences barely differed over time. The majority of respondents experienced good leadership of the supervisor and the manager with a median of 4. However, spreading regarding leadership of the manager was larger (IQR 3-6 compared to an IQR of 3-4 for supervisors), indicating more variability in answers. This can be explained by respondents from hospital B most frequently answered this question with ‘no opinion’ for all measurement moments.

Other job resources
Other job resources was measured by both hospitals with four questions and experiences barely differed over time. Medians of all questions and measurement moments was 4 (IQR: 3-4), indicating more than half of the respondents are neutral towards or agreed with the statements about a nice work environment and effectively collaboration with division/hospital/external parties.

**Engaged leadership**

Connecting
Experiencing connecting leaders was measured by hospital A with one question and barely differed over time, indicating (totally) agreement towards a good relationship with the supervisor with a median of 4 (IQR: 4-5).

Inspiring
Experiences of inspiring leaders were measured by both hospitals with two questions, and these experiences barely differed over time, indicating a neutral opinion/agreement towards statements about a motivating supervisor and role model behaviors, with a median of 4 (IQR: 3-4). The item ‘my supervisor motivates me’ demonstrates a change in median over time (3 IQR: 3-4), indicating some fluctuations in the perceived leadership.

**Personal resources**

Self-efficacy
Self-efficacy was measured by hospital B with one question and barely differed over time, indicating a neutral opinion/agreement towards the statement ‘I can effectively solve problems in my work’, with a median of 4 (IQR: 3-4).

Goal-directiveness
Goal-directiveness was measured by hospital B with one question and barely differed over time, indicating a neutral opinion/agreement towards the statement ‘I know what to do to achieve the aims within our teamwork’, with a median of 4 (IQR: 3-4).

**Employee well-being**

Boredom
Boredom was measured by hospital A with one question and differ slightly over time. However, this can be explained by various answer options used. Measurements in 2020 and 2022 shows a median of 4 (IQR: 4-4), revealing unanimity and thus overall agreement that work is challenging in a good way. However, in 2021, the median dropped to 3 (IQR: 3-4), indicating more variability and suggesting that work was only regularly/often challenging. Still the most frequently given answer was 4, in 39.54% of the cases ‘often’ was answered.

Burnout
Burnout was measured by hospital A with one question on a 10-point rating scale (1: exhausted – 10: vital). Experiences barely differed over time from a mean of 6.5 in 2021 to a mean of 6.4 in 2022.

Job satisfaction
Job satisfaction was measured by hospital A and B with the same question. Experiences barely differed over time with a median of 4 (IQR: 4-5). Strikingly, answer 6 (no opinion or not applicable) was given least often for this question.

Work engagement
Work engagement was measured by hospital A with one question on a 10-point rating scale (1: disengaged – 10: engaged). Experiences slightly decreased over time from a mean of 7.14 in 2021 to a mean of 6.94 in 2022.

Other employee well-being
Hospital B measured one other question on employee well-being: ‘I feel safe at work’. A median of 4 (IQR: 4-5) discovered for all questions and measurement moments, indicating (totally) agreement with this statement.

**Outcomes**

Commitment-organization
Commitment to the organization was measured by both hospitals with in total four questions, experiences barely differed over time. For all categorical questions a median of 4 (IQR: 3-4) revealed, indicating a neutral opinion/agreement towards the questions about ‘working for this hospital makes me proud’, ‘like to do something extra for work’ and ‘the success of my hospital is important to me’.
The fourth item was a 10-point rating scale (1: bad – 10: good), asking to rate overall experience for working at hospital B. Means over time vary from 7.29-7.31.

Commitment-teams
Commitment to the team was measured by hospital B with one question and experienced barely differ over time. A median of 4 (IQR: 3-4) discovered, indicating a neutral opinion/agreement towards the statement: ‘I prioritize the team results above my own ambitions’.

Workability
Work ability was measured by hospital A with one question and experiences barely differed over time. A median of 4 (IQR: 3-4) discovered, indicating a neutral opinion/agreement towards the statement: ‘I can do my work without negative consequences’.
