# Supplementary material for: Uncovering gaps in workforce well-being: a national look at survey practice in Dutch university medical centres – an exploratory quantitative study
Source: BMJ Open. 2025 Jul 18;15(7):e094939. doi: 10.1136/bmjopen-2024-094939 (PMC12273149; doi:10.1136/bmjopen-2024-094939)
Supplement: online supplemental file 5 [file bmjopen-15-7-s005.docx]

**Additional File 5**

*Table G Stratification for age and function on possibilities for workload items*

| **Question-item** | **Scale** | **Stratification level** | **Hospital A** | **Hospital A** | **Hospital A** | **Hospital B** | **Hospital B** | **Hospital B** | **Hospital B** |
| --- | --- | --- | --- | --- | --- | --- | --- | --- | --- |
|  |  |  | *Sept 2020 (4157)* | *Dec 2021 (4102)* | *July 2022 (3603)* | *Sept 2020 5056)* | *Jan 2022 (4842)* | *May 2022 (4894)* | *May 2023 (4895)* |
| There is an acceptable workload | (1) totally disagree; (2) disagree; (3) neutral; (4) agree; (5) totally agree; (6) not applicable | Age <30 | 4 (IQR 3-4) 1: 10 (1.76%) 2: 72 (12.65%) 3: 137 (24.08%) **4: 313 (55.01%)** 5: 36 (6.33%) 6: 1 (0.18%)  Total 569 |  | 4 (IQR 3-4)  1: 21 (4.44%)  2: 87 (18.39%)  3: 121 (25.58%)  **4: 208 (43.97%)**  5: 36 (7.61%)  6: 0 (0%)  Total: 473 |  |  |  |  |
|  |  | Age 30-39 | 4 (IQR 3-4) 1: 25 (2.66%) 2: 144 (15.30%) 3: 190 (20.19%) **4: 510 (54.20%)** 5: 72 (7.65%) 6: 0 (0%)  Total: 941 |  | 4 (IQR 3-4)  1: 32 (4.10%)  2: 157 (20.10%)  3: 173 (22.15%)  **4: 364 (46.61%)**  5: 55 (7.04%)  6: 0 (0%)  Total 781 |  |  |  |  |
|  |  | Age 40-49 | 4 (IQR 3-4) 1: 24 (2.47%)  2: 152 (15.67%)  3: 220 (22.68%)  **4: 504 (51.96%)**  5: 70 (7.22%)  6: 0 (0%)  Total: 970 |  | 4 (IQR 3-4)  1: 40 (4.29%)  2: 190 (20.39%)  3: 217 (23.29%)  **4: 428 (45.92%)**  5: 56 (6.01%)  6: 1 (0.11%)  Total 932 |  |  |  |  |
|  |  | Age 50-59 | 4 (IQR 3-4) 1: 28 (2.39%)  2: 194 (16.54%)  3: 260 (22.17%)  **4: 613 (52.26%)**  5: 77 (6.56%)  6: 1 (0.09%)  Total: 1173 |  | 4 (IQR 3-4)  1: 46 (4.58%)  2: 170 (16.93%)  3: 243 (24.20%)  **4: 465 (46.31%)**  5: 80 (7.97%)  6: 0 (0%)  Total: 1004 |  |  |  |  |
|  |  | Age 60+ | 4 (IQR 3-4)  1: 11 (2.18%)  2: 72 (14.29%)  3: 124 (24.60%)  **4: 243 (48.21%)**  5: 54 (10.70%)  6: 0 (0%)  Total: 504 |  | 3 (IQR 2-4)  1: 21 (5.08%)  2: 86 (20.82%)  3: 100 (24.21%)  **4: 170 (41.16%)**  5: 35 (8.47%)  6: 1 0.24%  Total: 413 |  |  |  |  |
|  |  | Nursing & Care | 4 (IQR 3-4)  1: 28 (3.14%)  2: 139 (15.58%)  3: 219 (24.55%)  **4: 456 (51.12%)**  5: 50 (5.60%)  6: 0 (0%)  Total: 892 |  | 3 (IQR 2-4)  1: 46 (6.02%)  2: 170 (22.25%)  3: 188 (24.61%)  **4: 315 (41.23%)**  5: 44 (5.76%)  6: 1 (0.13%)  Total: 764 |  |  |  |  |
|  |  | Clinical support | 4 (IQR 3-4) 1: 6 (1.64%)  2: 41 (11.23%)  3: 82 (22.47%)  **4: 208 (56.99%)**  5: 28 (7.67%))  6: 0 (0%)  Total: 365 |  | 3 (IQR 2-4)  1: 16 (5,21%)  2: 91 (29,64%)  3: 70 (22,80%)  **4: 117 (38,11%)**  5: 12 (3,91%)  6: 1 (0,32%)  Total: 307 |  |  |  |  |
|  |  | Clinical (co) treating | 4 (IQR 3-4) 1: 2 (0.72%)  2: 29 (10.43%)  3: 60 (21.58%)  **4: 171 (61.51%)**  5: 16 (5.76%)  6: 0 (0%)  Total: 278 |  | 4 (IQR 2-4)  1: 7 (3,26%)  2: 48 (22,33%)  3: 50 (23,26%)  **4: 106 (49,30%)**  5: 4 (1,86%)  6: 0 (0%)  Total: 215 |  |  |  |  |
|  |  | Analytics | 4 (IQR 3-4)  1: 3 (1.34%)  2: 25 (11.21%)  3: 28 (12.56%)  **4: 155 (69.51%)**  5: 10 (4.48%)  6: 0 (0%)  Total: 223 |  | 4 (IQR 3-4)  1: 12 (5,24%)  2: 27 (11,79%)  3: 48 (20,96%)  **4: 124 (54,15%)**  5: 18 (7,86%)  6: 0 (0%)  Total: 229 |  |  |  |  |
|  |  | Scientific research & education | 4 (IQR 2-4)  1: 13 (4.50%)  2: 62 (21.45%)  3: 62 (21.45%)  **4: 120 (41.52%)**  5: 31 (10.72%)  6: 0 (0%)  Total: 289 |  | 3 (IQR 2-4)  1: 14 (5,76%)  2: 51 (20,99%)  3: 65 (26,75%)  **4: 85 (34,98%)**  5: 28 (11,52%)  6: 0 (0%)  Total: 243 |  |  |  |  |
|  |  | Management | 4 (IQR 3-4)  1: 6 (2.67%)  2: 40 (17,78%)  3: 58 (25.78%)  **4: 92 (40.89%)**  5: 27(12%)  6: 0 (0%)  Total: 225 |  | 4 (IQR 3-4)  1: 7 (3,20%)  2: 37 (16,89%)  3: 48 (21,92%)  **4: 104 47,49%)**  5: 23 (10,50%)  6: 0 (0%)  Total: 219 |  |  |  |  |
|  |  | Staff, administration, secretariat | 4 (IQR 3-4)  1: 27 (2.39%)  2: 174 (15.40%)  3: 258 (22.83%)  **4: 586 (51.86%)**  5: 85 (7.52%)  6: 0 (0%)  Total: 1130 |  | 4 (IQR 3-4)  1: 27 (2,72%)  2: 138 (13,93%)  3: 228 (23,01%)  **4: 517 (52,17%)**  5: 81 (8,17%)  6: 0 (0%)  Total: 991 |  |  |  |  |
|  |  | Facility | 4 (IQR 3-4)  1: 3 (0.80%)  2: 44 (11.70%)  3: 75 (19.95%)  **4: 215 (57.18%)**  5: 39 (10.37%)  6: 0 (0%)  Total: 376 |  | 4 (IQR 3-4)  1: 11 (3,67%)  2: 37 (12,33%)  3: 64 (21,33%)  **4: 163 (54,33%)**  5: 25 (8,33%)  6: 0 (0%)  Total: 300 |  |  |  |  |
|  |  | Resident physicians | 4 (IQR 3-4)  1: 0 (0%)  2: 8 (10.96%)  3: 19 (26.03%)  **4: 43 (58.90%)**  5: 3 (4.11%)  6: 0 (0%)  Total: 73 |  | 3 (IQR 2-4)  1: 4 (6,45%)  2: 16 (25,81%)  3: 14 (22,58%)  **4: 26 (41,94%)**  5: 2 (3,23%)  6: 0 (0%)  Total: 62 |  |  |  |  |
|  |  | Medical specialists | 3 (IQR 2-4)  1: 9 (3.45%)  2: 68 (26.05%  3: 63 (24.14%)  **4: 106 (40.61%)**  5: 15 (5.75%)  6: 0 (0%)  Total: 261 |  | 3 (IQR 2-4)  1: 16 (6,61%)  2: 66 (27,27%)  **3: 73 (30,17%)**  4: 68 (28,10%)  5: 19 (7,85%)  6: 0 (0%)  Total: 242 |  |  |  |  |
| I have too much work | (1) never; (2) sometimes; (3) regularly; (4) often; (5) always; (6) I don’t know | Age <30 |  | 4 (IQR 3-4)  1: 25 (4,50%)  2: 68 (12,23%)  3: 119 (21,40%)  **4: 265 (47,66%)**  5: 77 (13,85%)  6: 2 (0,36%)  Total: 556 |  |  |  |  |  |
|  |  | Age 30-39 |  | 4 (IQR 3-4)  1: 50 (5,44%)  2: 149 (16,21%)  3: 215 (23,39%)  **4: 425 (46,25%)**  5: 74 (8,05%)  6: 6 (0,65%)  Total: 919 |  |  |  |  |  |
|  |  | Age 40-49 |  | 4 (IQR 2-4)  1: 95 (8,90%)  2: 180 (16,87%)  3: 241 (22,59%)  **4: 471 (44,14%)**  5: 78 (7,31%)  6: 2 (0,19%)  Total: 1067 |  |  |  |  |  |
|  |  | Age 50-59 |  | 4 (IQR 3-4)  1: 96 (8,73%)  2: 160 (14,55%)  3: 272 (24,73%)  **4: 501 (45,55%)**  5: 67 (6,09%)  6: 4 (0,36%)  Total: 1100 |  |  |  |  |  |
|  |  | Age 60+ |  | 4 (IQR 3-4)  1: 28 (6,09%)  2: 66 (14,35%)  3: 108 (23,48%)  **4: 218 (47,39%)**  5: 36 (7,83%)  6: 4 (0,87%)  Total: 460 |  |  |  |  |  |
|  |  | Nursing & Care |  | 4 (IQR 3-4)  1: 26 (2,99%)  2: 146 (16,76%)  3: 249 (28,59%)  **4: 402 (46,15%)**  5: 45 (5,17%)  6: 3 (0,34%)  Total: 871 |  |  |  |  |  |
|  |  | Clinical support |  | 4 (IQR 3-4)  1: 9 (2,68%)  2: 26 (7,74%)  3: 70 (20,83%)  **4: 185 (55,06%)**  5: 44 (13,10%)  6: 2 (0,60%)  Total: 336 |  |  |  |  |  |
|  |  | Clinical (co) treating |  | 4 (IQR 3-4)  1: 18 (7,20%)  2: 33 (13,20%)  3: 63 (25,20%)  **4: 115 (46,00%)**  5: 21 (8,40%)  6: 0 (0%)  Total: 250 |  |  |  |  |  |
|  |  | Analytics |  | 4 (IQR 3-4)  1: 11 (3,97%)  2: 23 (8,30%)  3: 37 (13,36%)  **4: 173 (62,45%)**  5: 29 (10,47%)  6: 4 (1,44%)  Total: 277 |  |  |  |  |  |
|  |  | Scientific research & education |  | 3 (IQR 2-4)  1: 61 (19,87%)  2: 60 (19,54%)  3: 63 (20,52%)  **4: 101 (32,90%)**  5: 20 (6,51%)  6: 2 (0,65%)  Total: 307 |  |  |  |  |  |
|  |  | Management |  | 4 (IQR 3-4)  1: 24 (0,11%)  2: 42 (19,27%)  3: 54 (24,77%)  **4: 84 (38,53%)**  5: 13 (5,96%)  6: 1 (0,46%)  Total: 218 |  |  |  |  |  |
|  |  | Staff, administration, secretariat |  | 4 (IQR 3-4)  1: 74 (6,70%)  2: 165 (14,95%)  3: 253 (22,92%)  **4: 508 (46,01%)**  5: 99 (8,97%)  6: 5 (0,45%)  Total: 1104 |  |  |  |  |  |
|  |  | Facility |  | 4(IQR 3-4)  1: 22 (5,95%)  2: 41 (11,08%)  3: 80 (21,62%)  **4: 187 (50,54%)**  5: 39 (10,54%)  6: 1 (0,27%)  Total: 370 |  |  |  |  |  |
|  |  | Resident physicians |  | 3 (IQR 2-4)  1: 5 (6,67%)  2: 17 (22,67%)  3: 17 (22,67%)  **4: 32 (42,67%)**  5: 4 (5,33%)  6: 0 (0%)  Total: 75 |  |  |  |  |  |
|  |  | Medical specialists |  | 3 (IQR 2-4)  1: 41 (16,14%)  2: 63 (24,80%)  3: 66 (25,98%)  **4: 79 (31,10%)**  5: 5 (1,97%)  6: 0 (0%)  Total: 254 |  |  |  |  |  |
| I think my workload is on an average base.. | (1) way too much, (2) too much, (3) too less, (4) way too less, (5) appropriate; (6) no opinion | Age <30 |  |  |  | x (IQR x-x)  1: 41 (3,95%)  2: 257 (24,74%)  3: 61 (5,87%)  4: 5 (0,48%)  **5: 656 (63,14%)**  6: 19 (1,83%)  Total: 1039 | x (IQR x-x)  1: 36 (3.88%)  2: 223 (24,03%)  3: 41 (4,42%)  4: 4 (0,43%)  **5: 604 (65,09%)**  6: 20 (2,16%)  Total: 928 | x (IQR x-x)  1: 39 (4,30%)  2: 235 (25,91%)  3: 45 (4,96%)  4: 4 (0,44%)  **5: 572 (63,07%)**  6: 12 (1,32%)  Total: 907 | x (IQR x-x)  1: 27 (2,94%)  2: 227 (24,70%)  3: 51 (5,55%)  4: 4 (0,44%)  **5: 592 (64,42%)**  6: 18 (1,96%)  Total: 919 |
|  |  | Age 30-39 |  |  |  | x (IQR x-x)  1: 65 (5,62%)  2: 368 (31,81%)  3: 50 (4,32%)  4: 3 (0,26%)  **5: 654 (56,53%)**  6: 17 (1,47%)  Total: 1157 | x (IQR x-x)  1: 64 (5,73%)  2: 366 (32,80%)  3: 45 (4,03%)  4: 6 (0,54%)  **5: 608 (54,48%)**  6: 27 (2,42%)  Total: 1116 | x (IQR x-x)  1: 52 (4,54 %)  2: 357 (31,15%)  3: 43 (3,75%)  4: 5 (0,44%)  **5: 670 (58,46%)**  6: 19 (1,66%)  Total: 1146 | x (IQR x-x)  1: 62 (5,32%)  2: 339 (29,07%)  3: 40 (3,43%)  4: 7 (0,60%)  **5: 697 (59,78%)**  6: 21 (1,80%)  Total: 1166 |
|  |  | Age 40-49 |  |  |  | x (IQR x-x)  1: 63 (6,40%)  2: 316 (32,08%)  3: 19 (1,93%)  4: 7 (0,71%)  **5: 552 (56,04%)**  6: 28 (2,84%)  Total: 985 | x (IQR x-x)  1: 68 (6,97%)  2: 309 (31,66%)  3: 24 (2,46%)  4: 2 (0,20%)  5: **546 (55,94%)**  6: 27 (2,77%)  Total: 976 | x (IQR x-x)  1: 67 (6,50%)  2: 365 (35,44%)  3: 16 (1,55%)  4: 5 (0,49%)  **5: 555 (53,88%)**  6: 22 (2,14%)  Total: 1030 | x (IQR x-x)  1: 74 (7,23%)  2: 348 (34,02%)  3: 17 (1,66%)  4: 2 (0,20%)  **5: 557 (54,45%)**  6: 25 (2,44%)  Total: 1023 |
|  |  | Age 50-59 |  |  |  | x (IQR x-x)  1: 96 (8,67%)  2: 331 (29,90%)  3: 22 (1,99%)  4: 6 (0,54%)  **5: 632 (57,09%)**  6: 20 (1,81%)  Total: 1107 | x (IQR x-x)  1: 76 (7,08%)  2: 332 (30,91%)  3: 27 (2,51%)  4: 2 (0,19%)  **5: 614 (57,17%)**  6: 23 (2,14%)  Total: 1074 | x (IQR x-x)  1: 58 (5,44%)  2: 317 (29,74%)  3: 11 (1,03%)  4: 3 (0,28%)  **5: 655 (61,44%)**  6: 22 (2,06%)  Total: 1066 | x (IQR x-x)  1: 58 (5,35%)  2: 353 (32,53%)  3: 14 (1,29%)  4: 2 (0,18%)  **5: 625 (57,60%)**  6: 33 (3,04%)  Total: 1085 |
|  |  | Age 60+ |  |  |  | x (IQR x-x)  1: 24 (4,99%)  2: 139 (28,90%)  3: 12 (2,49%)  4: 1 (0,21%)  **5: 286 (59,46%)**  6: 19 (3,95%)  Total: 481 | x (IQR x-x)  1: 18 (4,03%)  2: 114 (25,56%)  3: 8 (1,79%)  4: 0 (0%)  **5: 288 (64,57%)**  6: 18 (4,04%)  Total: 446 | x (IQR x-x)  1: 31 (7,13%)  2: 107 (24,60%)  3: 6 (1,38%)  4: 0 (0%)  **5: 277 (63,68%)**  6: 14 (3,22%)  Total: 435 | x (IQR x-x)  1: 25 (6,07%)  2: 102 (24,76%)  3: 7 (1,70%)  4: 1 (0,24%)  **5: 268 (65,05%)**  6: 9 (2,18%)  Total: 412 |
|  |  | Nursing & Care |  |  |  | x (IQR x-x)  1: 33 (4,02%)  2: 253 (30,82%)  3: 33 (4,02%)  4: 1 (0,12%)  **5: 493 (60,04%)**  6: 8 (0,97%)  Total: 821 | x (IQR x-x)  1: 28 (3,86%)  2: 197 (27,13%)  3: 26 (3,58%)  4: 0 (0%)  **5: 469 (64,60%)**  6: 6 (0,83%)  Total: 726 | x (IQR x-x)  1: 25 (3,11%)  2: 214 (26,65%)  3: 29 (3,61%)  4: 3 (0,37%)  **5: 528 (65,75%)**  6: 4 (0,50%)  Total: 803 | x (IQR x-x)  1: 28 (3,48%)  2: 211 (26,24%)  3: 29 (3,61%)  4: 1 (0,12%)  **5: 525 (65,30%)**  6: 10 (1,24%)  Total: 804 |
|  |  | Clinical support |  |  |  | x (IQR x-x)  1: 26 (6,22%)  2: 142 (33,97%)  3: 17 (4,07%)  4: 0 (0%)  **5: 226 (54,07%)**  6: 7 (1,67%)  Total: 418 | x (IQR x-x)  1: 20 (5,19%)  2: 115 (29,87%)  3: 16 (4,16%)  4: 2 (0,52%)  **5: 219 (56,88%)**  6: 13 (3,38%)  Total: 385 | x (IQR x-x)  1: 24 (5,77%)  2: 130 (31,25%)  3: 16 (3,85%)  4: 2 (0,48%)  **5: 239 (57,45%)**  6: 5 (1,20%)  Total: 416 | x (IQR x-x)  1: 26 (6,31%)  2: 125 (30,34%)  3: 13 (3,16%)  4: 2 (0,49%)  **5: 237 (57,52%)**  6: 9 (2,18%)  Total: 412 |
|  |  | Clinical (co) treating |  |  |  | x (IQR x-x)  1: 19 (6,15%)  2: 107 (34,63%)  3: 13 (4,21%)  4: 0 (0%)  **5: 167 (54,05%)**  6: 3 (0,97%)  Total: 309 | x (IQR x-x)  1: 32 (9,67%)  2: 94 (28,40%)  3: 16 (4,83%)  4: 2 (0,60%)  **5: 179 (54,08%)**  6: 8 (2,42%)  Total: 331 | x (IQR x-x)  1: 15 (4,48%)  2: 108 (32,24%)  3: 12 (3,58%)  4: 1 (0,30%)  **5: 192 (57,31%)**  6: 7 (2,09%)  Total: 335 | x (IQR x-x)  1: 19 (5,46%)  2: 117 (33,62%)  3: 12 (3,45%)  4: 2 (0,57%)  **5: 190 (54,60%)**  6: 8 (2,30%)  Total: 348 |
|  |  | Analytics |  |  |  | x (IQR x-x)  1: 29 (6,84%)  2: 107 (25,24%)  3: 21 (4,95%)  4: 5 (1,18%)  **5: 248 (58,49%)**  6: 14 (3,30%)  Total: 424 | x (IQR x-x)  1: 13 (3,47%)  2: 115 (30,67%)  3: 16 (4,27%)  4: 1 (0,27%)  **5: 218 (58,13%)**  6: 12 (3,20%)  Total: 375 | x (IQR x-x)  1: 11 (2,80%)  2: 101 (25,70%)  3: 9 (2,29%)  4: 4 (1,02%)  **5: 256 (65,14%)**  6: 12 (3,05%)  Total: 393 | x (IQR x-x)  1: 15 (3,59%)  2: 105 (25,12%)  3: 16 (3,83%)  4: 5 (1,20%)  **5: 268 (64,11%)**  6: 9 (2,15%)  Total: 418 |
|  |  | Scientific research & education |  |  |  | x (IQR x-x)  1: 27 (7,24%)  2: 133 (35,66%)  3: 3 (0,80%)  4: 0 (0%)  **5: 204 (54,69%)**  6: 6 (1,61%)  Total: 373 | x (IQR x-x)  1: 31 (8,01%)  2: 150 (38,76%)  3: 2 (0,52%)  4: 0 (0%)  **5: 200 (51,68%)**  6: 4 (1,03%)  Total: 387 | x (IQR x-x)  1: 20 (5,59%)  2: 155 (43,30%)  3: 8 (2,23%)  4: 0 (0%)  **5: 169 (47,21%)**  6: 6 (1,68%)  Total: 358 | x (IQR x-x)  1: 32 (7,96%)  2: 134 (33,33%)  3: 5 (1,24%)  4: 0 (0%)  **5: 224 (55,72%)**  6: 7 (1,74%)  Total: 402 |
|  |  | Management |  |  |  | x (IQR x-x)  1: 10 (4,88%)  2: 66 (32,20%)  3: 3 (1,46%)  4: 0 (0%)  **5: 125 (60,98%)**  6: 1 (0,49%)  Total: 205 | x (IQR x-x)  1: 12 (6,09.%)  2: 67 (34.01%)  3: 3 (1,52%)  4: 0 (0%)  **5: 109 (55,33%)**  6: 6 (3,05%)  Total: 197 | x (IQR x-x)  1: 20 (10,05%)  2: 66 (33,17%)  3: 1 (0,50%)  4: 1 (0,50%)  **5: 107 (53,77%)**  6: 4 (2,01%)  Total: 199 | x (IQR x-x)  1: 14 (8,09%)  2: 59 (34,10%)  3: 3 (1,73%)  4: 0 (0%)  **5: 96 (55,49%)**  6: 1 (0,58%)  Total: 173 |
|  |  | Staff, administration, secretariat |  |  |  | x (IQR x-x)  1: 67 (6,03%)  2: 295 (26,55%)  3: 37 (3,33%)  4: 9 (0,81%)  **5: 677 (60,94%)**  6: 26 (2,34%)  Total: 1111 | x (IQR x-x)  1: 59 (5,29%)  2: 294 (26,34%)  3: 36 (3,23%)  4: 4 (0,36%)  **5: 687 (61,56%)**  6: 36 (3,23%)  Total: 1116 | x (IQR x-x)  1: 68 (6,04%)  2: 306 (27,18%)  3: 27 (2,40%)  4: 6 (0,53%)  **5: 696 (61,81%)**  6: 23 (2,04%)  Total: 1126 | x (IQR x-x)  1: 64 (5,63%)  2: 320 (28,17%)  3: 31 (2,73%)  4: 3 (0,26%)  **5: 679 (59,77%)**  6: 39 (3,43%)  Total: 1136 |
|  |  | Facility |  |  |  | x (IQR x-x)  1: 42 (6,40%)  2: 165 (25,15%)  3: 21 (3,20%)  4: 5 (0,76%)  **5: 394 (60,06%)**  6: 29 (4,42%)  Total: 656 | x (IQR x-x)  1: 47 (7,86%)  2: 141 (23,58%)  3: 18 (3,01%)  4: 5 (0,84%)  **5: 361 (60,37%)**  6: 26 (4,35%)  Total: 598 | x (IQR x-x)  1: 42 (7,06%)  2: 164 (27,56%)  3: 17 (2,86%)  4: 2 (0,34%)  **5: 343 (57,65%)**  6: 27 (4,54%)  Total: 595 | x (IQR x-x)  1: 34 (5,96%)  2: 169 (29,65%)  3: 12 (2,11%)  4: 5 (0,88%)  **5: 334 (58,60%)**  6: 16 (2,81%)  Total: 570 |
|  |  | Resident physicians |  |  |  | x (IQR x-x)  1: 10 (5,56%)  2: 52 (28,89%)  3: 9 (5,00%)  4: 0 (0%)  **5: 107 (59,44%)**  6: 2 (1,11%)  Total: 180 | x (IQR x-x)  1: 4 (2,56%)  2: 55 (35,26%)  3: 4 (2,56%)  4: 0 (0%)  **5: 93 (59,62%)**  6: 0 (0%)  Total: 156 | x (IQR x-x)  1: 1 (0,75%)  2: 50 (37,59%)  3: 1 (0,75%)  4: 0 (0%)  **5: 81 (60,90%)**  6: 0 (0%)  Total: 133 | x (IQR x-x)  1: 7 (4,70%)  2: 39 (26,17%)  3: 4 (2,68%)  4: 0 (0%)  **5: 98 (65,77%)**  6: 1 (0,67%)  Total: 149 |
|  |  | Medical specialists |  |  |  | x (IQR x-x)  1: 30 (9,29%)  2: 131 (40,56%)  3: 6 (1,86%)  4: 2 (0,62%)  **5: 152 (47,06%)**  6: 2 (0,62%)  Total: 323 | x (IQR x-x)  1: 26 (7,65%)  2: 145 (42,65%)  3: 9 (2,65%)  4: 0 (0%)  **5: 155 (45,59%)**  6: 5 (1,47%)  Total: 340 | x (IQR x-x)  1: 31 (9,75%)  2: 137 (43,08%)  3: 4 (1,26%)  4: 0 (0%)  **5: 144 (45,28%)**  6: 2 (0,63%)  Total: 318 | x (IQR x-x)  1: 27 (10,15%)  2: 107 (40,23%)  3: 5 (1,88%)  4: 0 (0%)  **5: 125 (46,99%)**  6: 2 (0,75%)  Total: 266 |

*Tabel H Stratification for age and function on possibilities for learning and development items*

| Question-item | Scale | Stratification level | Hospital A | Hospital A | Hospital A | Hospital B | Hospital B | Hospital B | Hospital B |
| --- | --- | --- | --- | --- | --- | --- | --- | --- | --- |
|  |  |  | Sept 2020 (4157) | Dec 2021 (4102) | July 2022 (3603) | Sept 2020 (5056) | Jan 2022 (4842) | May 2022 (4894) | May 2023 (4895) |
| I am given the opportunity to develop | (1) totally disagree; (2) disagree; (3) neutral; (4) agree; (5) totally agree; (6) not applicable | Age <30 | 4 (IQR 3-4)  1: 5 (0,88%)  2: 44 (7,73%)  3: 104 (18,28%)  **4: 278 (48,86%)**  5: 136 (23,90%)  6: 2 (0,35%)  Total: 569 |  | 4 (IQR 4-4)  1: 4 (0,85%)  2: 37 (7,82%)  3: 73 (15,43%)  **4: 256 (54,12%)**  5: 103 (21,78%)  6: 0 (0%)  Total: 473 |  |  |  |  |
|  |  | Age 30-39 | 4 (IQR 3-4)  1: 17 (1,81%)  2: 72 (7,65%)  3: 176 (18,70%)  **4: 483 (51,32%)**  5: 192 (20,40%)  6: 1 (0,11%)  Total: 941 |  | 4 (IQR 3-4)  1: 21 (2,69%)  2: 86 (11,01%)  3: 176 (22,54%)  **4: 357 (45,71%)**  5: 141 (18,05%)  6: 0 (0%)  Total: 781 |  |  |  |  |
|  |  | Age 40-49 | 4 (IQR 3-4)  1: 21 (2,16%)  2: 92 (9,48%)  3: 197 (20,31%)  **4: 473 (48,76%)**  5: 186 (19,18%)  6: 1 (0,10%)  Total: 970 |  | 4 (IQR 3-4)  1: 18 (1,93%)  2: 114 (12,23%)  3: 194 (20,82%)  **4: 440 (47,21%)**  5: 163 (17,49%)  6: 3 (0,32%)  Total: 932 |  |  |  |  |
|  |  | Age 50-59 | 4 (IQR 3-4)  1: 18 (1,53%)  2: 102 (8,70%)  3: 270 (23,02%)  **4: 583 (49,70%)**  5: 199 (16,97%)  6: 1 (0,09%)  Total: 1173 |  | 4 (IQR 3-4)  1: 32 (3,19%)  2: 105 (10,46%)  3: 240 (23,90%)  **4: 477 (47,51%)**  5: 147 (14,64%)  6: 3 (0,30%)  Total: 1004 |  |  |  |  |
|  |  | Age 60+ | 4 (IQR 3-4)  1: 6 (1,19%)  2: 30 (5,95%)  3: 144 (28,57%)  **4: 245 (48,61%)**  5: 76 (15,08%)  6: 3 (0,60%)  Total: 504 |  | 4 (IQR 3-4)  1: 7 (1,69%)  2: 35 (8,47%)  3: 111 (26,88%)  **4: 188 (45,52%)**  5: 67 (16,22%)  6: 4 (0,97%)  Total: 413 |  |  |  |  |
|  |  | Nursing & Care | x (IQR x-x)  1: 14 (1,57%)  2: 69 (7,74%)  3: 184 (20,63%)  **4: 450 (50,45%)**  5: 174 (19,51%)  6: 1 (0,11%)  Total: 892 |  | 4 (IQR 3-4)  1: 9 (1,18%)  2: 67 (8,77%)  3: 164 (21,47%)  **4: 413 (54,06%)**  5: 109 (14,27%)  6: 2 (0,26%)  Total: 764 |  |  |  |  |
|  |  | Clinical support | 4 (IQR 3-4)  1: 10 (2,74%)  2: 43 (11,78%)  3: 111 (30,41%)  **4: 163(44,66%)**  5: 37 (10,14%)  6: 1 (0,27%)  Total: 365 |  | 4 (IQR 3-4)  1: 20 (6,51%)  2: 47 (15,31%)  3: 83 (27,04%)  **4: 131 (42,67%)**  5: 21 (6,84%)  6: 5 (1,63%)  Total: 307 |  |  |  |  |
|  |  | Clinical (co) treating | 4 (IQR 3-4)  1: 2 (0,72%)  2: 20 (7,19%)  3: 60 (21,58%)  **4: 151 (54,32%)**  5: 45 (16,19%)  6: 0 (0%)  Total: 278 |  | 4 (IQR 3-4)  1: 7 (3,26%)  2: 39 (18,14%)  3: 61 (28,37%)  **4: 86 (40,00%)**  5: 22 (10,23%)  6: 0 (0%)  Total: 215 |  |  |  |  |
|  |  | Analytics | 4 (IQR 3-4)  1: 8 (3,59%)  2: 36 (16,14%)  3: 67 (30,04%)  **4: 95 (42,60%)**  5: 16 (7,17%)  6: 1 (0,45%)  Total: 223 |  | 3 (IQR 3-4)  1: 10 (4,37%)  2: 40 (17,47%)  3: 74 (32,31%)  **4: 87 (37,99%)**  5:- 18 (7,86%)  6: 0 (0%)  Total: 229 |  |  |  |  |
|  |  | Scientific research & education | 4 (IQR 3-4)  1: 4 (1,38%)  2: 15 (5,19%)  3: 41 (14,19%)  **4: 133 (46,02%)**  5: 94 (32,52%)  6: 2 (0,69%)  Total: 289 |  | 4 (IQR 4-5)  1: 5 (2,06%)  2: 19 (7,82%)  3: 33 (13,58%)  **4: 112 (46,09%)**  5: 73 (30,04%)  6: 1 (0,41%)  Total: 243 |  |  |  |  |
|  |  | Management | 4 (IQR 4-5)  1: 0 (0%)  2: 5 (2,22%)  3: 30 (13,33%)  **4: 120 (53,33%)**  5: 70 (31,11%)  6: 0 (0%)  Total: 225 |  | 4 (IQR 4-5)  1: 1 (0,46%)  2: 12 (5,48%)  3: 22 (10,05%)  **4: 113 (51,60%)**  5: 71 (32,42%)  6: 0 (0%)  Total: 219 |  |  |  |  |
|  |  | Staff, administration, secretariat | 4 (IQR 3-4)  1: 16 (1,42%)  2: 93 (8,23%)  3: 259 (22,92%)  **4: 567 (50,18%)**  5: 195 (17,26%)  6: 0 (0%)  Total: 1130 |  | 4 (IQR 3-4)  1: 14 (1,41%)  2: 94 (9,49%)  3: 235 (23,71%)  **4: 478 (48,23%)**  5: 168 (16,95%)  6: 2 (0,20%)  Total: 991 |  |  |  |  |
|  |  | Facility | 4 (IQR 3-4)  1: 10 (2,66%)  2: 35 (9,31%)  3: 79 (21,01%)  **4: 180 (47,87%)**  5: 72 (19,15%)  6: 0 (0%)  Total: 376 |  | 4 (IQR 3-4)  1: 8 (2,67%)  2: 31 (10,33%)  3: 54 (18,00%)  **4: 147 (49,00%)**  5: 59 (19,67%)  6: 1 (0,33%)  Total: 300 |  |  |  |  |
|  |  | Resident physicians | 4 (IQR 4-4)  1: 0 (0%)  2: 0 (0%)  3: 8 (10,95%)  **4: 49 (67,12%)**  5: 15 (20,55%)  6: 1 (1,37%)  Total: 73 |  | 4 (IQR 3-4)  1: 0 (0%)  2: 5 (8,06%)  3: 13 (20,97%)  **4: 32 (51,61%)**  5: 12 (19,35%)  6: 0 (0%)  Total: 62 |  |  |  |  |
|  |  | Medical specialists | 4 (IQR 3-4)  1: 3 (1,15%)  2: 23 (8,81%)  3: 43 (16,48%)  **4: 132 (50,57%)**  5: 60 (22,99%)  6: 0 (0%)  Total: 261 |  | 4 (IQR 3-4)  1: 6 (2,48%)  2: 20 (8,26%)  3: 48 (19,83%)  **4: 105 (43,39%)**  5: 63 (26,03%)  6: 0 (0%)  Total: 242 |  |  |  |  |
| I get opportunity to learn and develop knowledge and skills | (1) totally disagree; (2) disagree; (3) neutral; (4) agree; (5) totally agree; (6) I don’t know | Age <30 |  | 4 (IQR 3-4)  1: 6 (1,08%)  2: 68 (12,23%)  3: 149 (26,80%)  **4: 262 (47,12%)**  5: 69 (12,41%)  6: 2 (0,36%)  Total: 556 |  |  |  |  |  |
|  |  | Age 30-39 |  | 4 (IQR 3-4)  1: 11 (1,20%)  2: 144 (15,67%)  3: 244 (26,55%)  **4: 421 (45,81%)**  5: 96 (10,45%)  6: 3 (0,33%)  Total: 919 |  |  |  |  |  |
|  |  | Age 40-49 |  | 4 (IQR 3-4)  1: 18 (1,69%)  2: 140 (13,12%)  3: 299 (28,02%)  **4: 451 (42,27%)**  5: 151 (14,15%)  6: 8 (0,75%)  Total: 1067 |  |  |  |  |  |
|  |  | Age 50-59 |  | 4 (IQR 3-4)  1: 20 (1,82%)  2: 164 (14,91%)  3: 300 (27,27%)  **4: 466 (42,36%)**  5: 146 (13,27%)  6: 4 (0,36%)  Total: 1100 |  |  |  |  |  |
|  |  | Age 60+ |  | 4 (IQR 3-4)  1: 6 (1,30%)  2: 47 (10,22%)  3: 139 (30,22%)  **4: 177 (38,48%)**  5: 86 (18,70%)  6: 5 (1,09%)  Total: 460 |  |  |  |  |  |
|  |  | Nursing & Care |  | 4 (IQR 3-4)  1: 11 (1,26%)  2: 111 (12,74%)  3: 269 (30,88%)  **4: 396 (45,46%)**  5: 81 (9,30%)  6: 3 (0,34%)  Total: 871 |  |  |  |  |  |
|  |  | Clinical support |  | 4 (IQR 3-4)  1: 6 (1,79%)  2: 77 (22,92%)  3: 104 (30,95%)  **4: 126 (37,5%)**  5: 19 (5,65%)  6: 4 (1,19%)  Total: 336 |  |  |  |  |  |
|  |  | Clinical (co) treating |  | 4 (IQR 3-4)  1: 0 (0%)  2: 23 (9,20%)  3: 81 (32,40%)  **4: 110 (44,00%)**  5: 36 (14,40%)  6: 0 (0%)  Total: 250 |  |  |  |  |  |
|  |  | Analytics |  | 4 (IQR 3-4)  1: 3 (1,08%)  2: 54 (19,49%)  3: 77 (27,80%)  **4: 122 (44,04%)**  5: 18 (6,50%)  6: 3 (1,08%)  Total: 277 |  |  |  |  |  |
|  |  | Scientific research & education |  | 4 (IQR 4-5)  1: 2 (0,65%)  2: 21 (6,84%)  3: 49 (15,96%)  **4: 151 (49,19%)**  5: 83 (27,04%)  6: 1 (0,33%)  Total: 307 |  |  |  |  |  |
|  |  | Management |  | 4 (IQR 4-4)  1: 2 (0,92%)  2: 14 (6,42%)  3: 36 (16,51%)  **4: 115 (52,75%)**  5: 51 (23,39%)  6: 0 (0%)  Total: 218 |  |  |  |  |  |
|  |  | Staff, administration, secretariat |  | 4 (IQR 3-4)  1: 19 (1,72%)  2: 175 (15,85%)  3: 325 (29,44%)  **4: 434 (39,31%)**  5: 144 (13,04%)  6: 7 (0,63%)  Total: 1104 |  |  |  |  |  |
|  |  | Facility |  | 4 (IQR 3-4)  1: 16 (4,32%)  2: 71 (19,19%)  3: 114 (30,81%)  **4: 127 (34,32%)**  5: 38 (10,27%)  6: 4 (1,08%)  Total: 370 |  |  |  |  |  |
|  |  | Resident physicians |  | 4 (IQR 3-4)  1: 0 (0%)  2: 4 (5,33%)  3: 19 (25,33%)  4**: 45 (60,00%)**  5: 7 (9,33%)  6: 0 (0%)  Total: 75 |  |  |  |  |  |
|  |  | Medical specialists |  | 4 (IQR 4-5)  1: 1 (0,39%)  2: 6 (2,36%)  3: 49 (19,29%)  **4: 133 (52,36%)**  5: 65 (25,59%)  6: 0 (0%)  Total: 254 |  |  |  |  |  |
| I can develop in my work | (1) totally disagree; (2) disagree; (3) neutral; (4) agree; (5) totally agree; (6) no opinion | Age <30 |  |  |  | 4 (IQR 4-4)  1: 18 (1,73%)  2: 92 (8,85%)  3: 148 (14,24%)  **4: 553 (53,22%)**  5: 223 (21,46%)  6: 5 (0,48%)  Total: 1039 | 4 (IQR 3-5)  1: 13 (1,40%)  2: 108 (11,64%)  3: 139 (14,98%)  **4: 433 (46,66%)**  5: 231 (24,89%)  6: 4 (0,43%)  Total: 928 | 4 (IQR 4-4)  1: 16 (1,76%)  2: 87 (9,59%)  3: 121 (13,34%)  **4: 465 (51,27%)**  5: 213 (23,48%)  6: 5 (0,55%)  Total: 907 | 4 (IQR 4-4)  1: 12 (1,31%)  2: 71 (7,73%)  3: 111 (12,08%)  **4: 504 (54,84%)**  5: 216 (23,50%)  6: 5 (0,54%)  Total: 919 |
|  |  | Age 30-39 |  |  |  | 4 (IQR 3-4)  1: 24 (2,07%)  2: 121 (10,46%)  3: 167 (14,43%)  **4: 596 (51,51%)**  5: 245 (21,18%)  6: 4 (0,35%)  Total: 1157 | 4 (IQR 3-4)  1: 21 (1,88%)  2: 111 (9,95%)  3: 172 (15,41%)  **4: 591 (52,96%)**  5: 216 (19,35%)  6: 5 (0,45%)  Total: 1116 | 4 (IQR 4-4)  1: 14 (1,22%)  2: 104 (9,08%)  3: 155 (13,53%)  **4: 643 (56,11%)**  5: 227 (19,81%)  6: 3 (0,26%)  Total: 1146 | 4 (IQR 3-4)  1: 26 (2,33%)  2: 116 (10,39%)  3: 172 (15,41%)  **4: 630 (56,45%)**  5: 214 (19,18%)  6: 9 (0,81%)  Total: 1116 |
|  |  | Age 40-49 |  |  |  | 4 (IQR 3-4)  1: 23 (2,34%)  2: 95 (9,64%)  3: 163 (16,55%)  **4: 525 (54,40%)**  5: 170 (17,26%)  6: 9 (0,91%)  Total: 985 | 4(IQR 3-4)  1: 20 (2,05%)  2: 98 (10,04%)  3: 185 (18,95%)  **4: 519 (53,18%)**  5: 146 (14,96%)  6: 8 (0,82%)  Total: 976 | 4 (IQR 3-)4  1: 17 (1,65%)  2: 115 (11,17%)  3: 175 (16,99%)  **4: 550 (53,40%)**  5: 169 (16,41%)  6: 4 (0,39%)  Total: 1030 | 4 (IQR 3-4)  1: 35 (3,42%)  2: 87 (8,50%)  3: 178 (17,40%)  **4: 553 (54,06%)**  5: 164 (16,03%)  6: 6 (0,59%)  Total: 1023 |
|  |  | Age 50-59 |  |  |  | 4 (IQR 3-4)  1: 26 (2,35%)  2: 100 (9,03%)  3: 260 (23,49%)  **4: 602 (54,38%)**  5: 109 (9,85%)  6: 10 (0,90%)  Total: 1107 | 4 (IQR 3-4)  1: 21 (1,96%)  2: 108 (10,06%)  3: 235 (21,88%)  **4: 582 (54,19%)**  5: 119 (11,08%)  6: 9 (0,84%)  Total: 1074 | 4 (IQR 3-4)  1: 17 (1,59%)  2: 103 (9,66%)  3: 259 (24,30%)  **4: 566 (53,10%)**  5: 112 (10,51%)  6: 9 (0,84%)  Total: 1066 | 4 (IQR 3-4)  1: 25 (2,30%)  2: 96 (8,85%)  3: 249 (22,95%)  **4: 582 (53,64%)**  5: 120 (11,06%)  6: 13 (1,20%)  Total: 1085 |
|  |  | Age 60+ |  |  |  | 4 (IQR 3-4)  1: 15 (3,12%)  2: 36 (7,48%)  3: 115 (23,91%)  **4: 259 (53,85%)**  5: 43 (8,94%)  6: 13 (2,70%)  Total: 481 | 4 (IQR 3-4)  1: 12 (2,69%)  2: 33 (7,40%)  3: 99 (22,20%)  **4: 248 (55,61%)**  5: 41 (9,19%)  6: 13 (2,91%)  Total: 446 | 4 (IQR 3-4)  1: 10 (2,30%)  2: 24 (5,52%)  3: 108 (24,83%)  **4: 245 (56,32%)**  5: 39 (8,97%)  6: 9 (2,07%)  Total: 435 | 4 (IQR 3-4)  1: 11 (2,67%)  2: 29 (7,04%)  3: 102 (24,76%)  **4: 223 (54,13%)**  5: 36 (8,74%)  6: 11 (2,67%)  Total: 412 |
|  |  | Nursing & Care |  |  |  | 4 (IQR 3-4)  1: 11 (1,34%)  2: 84 (10,23%)  3: 137 (16,69%)  **4: 468 (57,00%)**  5: 117 (14,25%)  6: 4 (0,49%)  Total: 821 | 4 (IQR 3-4)  1: 12 (1,65%)  2: 84 (11,57%)  3: 141 (19,42%)  **4: 405 (55,79%)**  5: 81 (11,16%)  6: 3 (0,41%)  Total: 726 | 4 (IQR 3-4)  1: 7 (0,87%)  2: 66 (8,22%)  3: 139 (17,31%)  **4: 480 (50,78%)**  5: 108 (13,45%)  6: 3 (0,37%)  Total: 803 | 4 (IQR 3-4)  1: 11 (1,37%)  2: 80 (9,95%)  3: 145 (18,03%)  **4: 441 (54,85%)**  5: 124 (15,42%)  6: 3 (0,37%)  Total: 804 |
|  |  | Clinical support |  |  |  | 4 (IQR 3-4)  1: 15 (3,59%)  2: 61 (14,59%)  3: 80 (19,14%)  **4: 222 (53,11%)**  5: 33 (7,89%)  6: 7 (1,67%)  Total: 418 | 4 (IQR 3-4)  1: 16 (4,16%)  2: 58 (15,06%)  3: 94 (24,42%)  **4: 187 (48,57%)**  5: 28 (7,27%)  6: 2 (0,52%)  Total: 385 | 4 (IQR 3-4)  1: 6 (1,44%)  2: 70 (16,83%)  3: 94 (22,60%)  **4: 211 (50,72%)**  5: 32 (7,69%)  6: 3 (0,72%)  Total: 416 | 4 (IQR 3-4)  1: 18 (4,37%)  2: 40 (9,71%)  3: 95 (23,06%)  **4: 218 (52,91%)**  5: 35 (8,50%)  6: 6 (1,46%)  Total: 412 |
|  |  | Clinical (co) treating |  |  |  | 4 (IQR 4-4)  1: 4 (1,29%)  2: 23 (7,44%)  3: 44 (14,24%)  **4: 199 (64,40%)**  5: 38 (12,30%)  6: 1 (0,32%)  Total: 309 | 4 (IQR 3-4)  1: 6 (1,81%)  2: 39 (11,78%)  3: 62 (18,73%)  **4: 182 (54,98%)**  5: 40 (12,08%)  6: 2 (0,60%)  Total: 331 | 4 (IQR 3-4)  1: 5 (1,49%)  2: 30 (8,96%)  3: 51 (15,22%)  **4: 202 (60,30%)**  5: 46 (13,73%)  6: 1 (0,30%)  Total: 335 | 4 (IQR 3-4)  1: 7 (2,01%)  2: 32 (9,20%)  3: 63 (18,10%)  **4: 196 (56,32%)**  5: 47 (13,51%)  6: 3 (0,86%)  Total: 348 |
|  |  | Analytics |  |  |  | 4 (IQR 3-4)  1: 29 (6,84%)  2: 58 (13,68%)  3: 99 (23,35%)  **4: 195 (45,99%)**  5: 39 (9,20%)  6: 4 (0,94%)  Total: 424 | 4 (IQR 3-4)  1: 7 (1,87%)  2: 60 (16,00%)  3: 90 (24,00%)  **4: 189 (50,40%)**  5: 29 (7,33%)  6: 0 (0%)  Total: 375 | 4 (IQR 3-4)  1: 15 (3,82%)  2: 54 (13,74%)  3: 86 (21,88%)  **4: 202 (51,40%)**  5: 33 (8,40%)  6: 3 (0,76%)  Total: 393 | 4 (IQR 3-4)  1: 14 (3,35%)  2: 62 (14,83%)  3: 113 (27,03%)  **4: 201 (48,09%)**  5: 23 (5,50%)  6: 5 (1,20%)  Total: 418 |
|  |  | Scientific research & education |  |  |  | 4 (IQR 4-5)  1: 1 (0,27%)  2: 21 (5,63%)  3: 50 (13,40%)  **4: 196 (52,55%)**  5: 102 (27,35%)  6: 3 (0,80%)  Total: 373 | 4 (IQR 4-5)  1: 2 (0,52%)  2: 15 (3,88%)  3: 45 (11,63%)  **4: 205 (52,97%)**  5: 117 (30,23%)  6: 3 (0,78%)  Total: 387 | 4 (IQR 4-5)  1: 1 (0,28%)  2: 18 (5,03%)  3: 50 (13,97%)  **4: 189 (52,79%)**  5: 99 (27,65%)  6: 1 (0,28%)  Total: 358 | 4 (IQR 4-5)  1: 3 (0,75%)  2: 12 (2,99%)  3: 46 (11,44%)  **4: 232 (57,71%)**  5: 106 (26,37%)  6: 3 (0,75%)  Total: 402 |
|  |  | Management |  |  |  | 4 (IQR 4-4)  1: 1 (0,49%)  2: 11 (5,37%)  3: 26 (12,68%)  **4: 119 (58,05%)**  5: 47 (22,93%)  6: 1 (0,49%)  Total: 205 | 4 (IQR 4-4)  1: 6 (3,05%)  2: 14 (7,11%)  3: 25 (12,69%)  **4: 111 (56,35%)**  5: 41 (20,81%)  6: 0 (0%)  Total: 197 | 4 (IQR 4-4)  1: 3 (1,51%)  2: 8 (4,02%)  3: 25 (12,57%)  **4: 117 (58,79%)**  5: 45 (22,61%)  6: 1 (0,50%)  Total: 199 | 4 (IQR 4-4)  1: 3 (1,73%)  2: 9 (5,20%)  3: 15 (8,67%)  **4: 106 (61,27%)**  5: 39 (22,54%)  6: 1 (0,58%)  Total: 173 |
|  |  | Staff, administration, secretariat |  |  |  | 4 (IQR 3-4)  1: 21 (1,89%)  2: 102 (9,18%)  3: 245 (22,05%)  **4: 601 (54,10%)**  5: 132 (11,88%)  6: 10 (0,90%)  Total: 1111 | 4 (IQR 3-4)  1: 19 (1,70%)  2: 110 (9,86%)  3: 221 (19,80%)  **4: 595 (53,32%)**  5: 157 (14,07%)  6: 14 (1,25%)  Total: 1116 | 4 (IQR 3-4)  1: 22 (1,95%)  2: 120 (10,66%)  3: 240 (21,31%)  **4: 585 (51,95%)**  5: 146 (12,97%)  6: 13 (1,15%)  Total: 1126 | 4 (IQR 3-4)  1: 27 (2,38%)  2: 106 (9,33%)  3: 217 (19,10%)  **4: 631 (55,55%)**  5: 142 (12,50%)  6: 13 (1,14%)  Total: 1136 |
|  |  | Facility |  |  |  | 4 (IQR 3-4)  1: 28 (4,27%)  2: 69 (10,52%)  3: 151 (23,02%)  **4: 314 (47,87%)**  5: 80 (12,20%)  6: 14 (2,13%)  Total: 656 | 4 (IQR 3-4)  1: 20 (3,34%)  2: 67 (11,20%)  3: 132 (22,07%)  **4: 301 (50,33%)**  5: 67 (11,20%)  6: 11 (1,84%)  Total: 598 | 4 (IQR 3-4)  1: 17 (2,86%)  2: 74 (12,44%)  3: 126 (21,18%)  **4: 294 (49,41%)**  5: 77 (12,94%)  6: 7 (1,18%)  Total: 595 | 4 (IQR 3-4)  1: 24 (4,21%)  2: 63 (11,05%)  3: 117 (20,53%)  **4: 289 (50,70%)**  5: 65 (11,40%)  6: 12 (2,11%)  Total: 570 |
|  |  | Resident physicians |  |  |  | 4 (IQR 4-5)  1: 0 (0%)  2: 5 (2,78%)  3: 15 (8,33%)  **4: 96 (53,33%)**  5: 64 (35,56%)  6: 0 (0%)  Total: 180 | 4 (IQR 4-5)  1: 0 (0%)  2: 6 (3,85%)  3: 5 (3,21%)  **4: 78 (50%)**  5: 67 (42,95%)  6: 0 (0%)  Total: 156 | 4 (IQR 4-5)  1: 0 (0%)  2: 4 (3,01%)  3: 9 (6,77%)  **4: 62 (46,62%)**  5: 58 (43,61%)  6: 0 (0%)  Total: 133 | 4 (IQR 4-5)  1: 2 (1,34%)  2: 3 (2,01%)  3: 16 (10,74%)  **4: 89 (59,73%)**  5: 39 (26,17%)  6: 0 (0%)  Total: 149 |
|  |  | Medical specialists |  |  |  | 4 (IQR 4-4)  1: 1 (0,31%)  2: 28 (8,67%)  3: 42 (13,00%)  **4: 172 (53,25%)**  5: 79 (24,46%)  6: 1 (0,31%)  Total: 323 | 4 (IQR 4-4)  1: 3 (0,88%)  2: 31 (9,12%)  3: 50 (14,71%)  **4: 172 (50,59%)**  5: 80 (23,53%)  6: 4 (1,18%)  Total: 340 | 4 (IQR 3-4)  1: 1 (0,31%)  2: 24 (7,55%)  3: 47 (17,92%)  **4: 169 (53,14%)**  5: 77 (24,21%)  6: 0 (0%)  Total: 318 | 4 (IQR 4-4)  1: 5 (1,88%)  2: 17 (6,39%)  3: 38 (14,29%)  **4: 143 (53,76%)**  5: 63 (23,68%)  6: 0 (0%)  Total: 266 |
| I can continuously improve in my work | (1) totally disagree; (2) disagree; (3) neutral; (4) agree; (5) totally agree; (6) no opinion | Age <30 |  |  |  | 4 (IQR 3-4)  1: 7 (0,67%)  2: 62 (5,97%)  3: 192 (18,48%)  **4: 588 (56,59%)**  5: 183 (17,61%)  6: 7 (0,67%)  Total: 1039 | 4 (IQR 3-4)  1: 5 (0,54%)  2: 82 (8,84%)  3: 157 (16,92%)  **4: 500 (53,88%)**  5: 179 (19,29%)  6: 5 (0,54%)  Total: 928 | 4 (IQR 4-4)  1: 6 (0,66%)  2: 60 (6,62%)  3: 157 (17,31%)  **4: 514 (56,67%)**  5: 168 (18,52%)  6: 2 (0,22%)  Total: 907 | 4 (IQR 4-4)  1: 5 (0,54%)  2: 48 (5,22%)  3: 153 (16,65%)  **4: 532 (57,89%)**  5: 178 (19,37%)  6: 3 (0,33%)  Total: 919 |
|  |  | Age 30-39 |  |  |  | 4 (IQR 3-4)  1: 15 (1,30%)  2: 84 (7,26%)  3: 208 (17,98%)  **4: 654 (56,53%)**  5: 191 (16,51%)  6: 5 (0,43%)  Total: 1157 | 4 (IQR 3-4)  1: 7 (0,63%)  2: 72 (6,45%)  3: 221 (19,80%)  **4: 633 (56,72%)**  5: 176 (15,77%)  6: 7 (0,63%)  Total: 1116 | 4 (IQR 4-4)  1: 6 (0,52%)  2: 67 (5,85%)  3: 209 (18,24%)  **4: 672 (58,64%)**  5: 185 (16,14%)  6: 7 (0,61%)  Total: 1146 | 4 (IQR 3-4)  1: 10 (0,86%)  2: 88 (7,55%)  3: 220 (18,87%)  **4: 662 (56,78%)**  5: 178 (15,27%)  6: 8 (0,69%)  Total: 1166 |
|  |  | Age 40-49 |  |  |  | 4 (IQR 3-4)  1: 15 (1,52%)  2: 69 (7,01%)  3: 181 (18,38%)  **4: 566 (57,46%)**  5: 149 (15,13%)  6: 5 (0,51%)  Total: 985 | 4 (IQR 3-4)  1: 11 (1,13%)  2: 77 (7,89%)  3: 192 (19,67%)  **4: 565 (57,89%)**  5: 120 (12,30%)  6: 11 (1,13%)  Total: 976 | 4 (IQR 3-4)  1: 8 (0,78%)  2: 82 (7,96%)  3: 182 (17,67%)  **4: 603 (58,54%)**  5: 152 (14,76%)  6: 3 (0,29%)  Total: 1030 | 4 (IQR 3-4)  1: 19 (1,86%)  2: 52 (5,08%)  3: 209 (20,43%)  **4: 599 (58,55%)**  5: 138 (13,49%)  6: 6 (0,59%)  Total: 1023 |
|  |  | Age 50-59 |  |  |  | 4 (IQR 3-4)  1: 9 (0,81%)  2: 84 (7,59%)  3: 270 (24,39%)  **4: 618 (55,83%)**  5: 118 (10,66%)  6: 8 (0,72%)  Total: 1107 | 4 (IQR 3-4)  1: 10 (0,93%)  2: 68 (6,33%)  3: 254 (23,65%)  **4: 614 (57,17%)**  5: 118 (10,99%)  6: 10 (0,93%)  Total:1074 | 4 (IQR 3-4)  1: 6 (0,56%)  2: 72 (6,75%)  3: 249 (23,36%)  **4: 627 (58,82%)**  5: 104 (9,75%)  6: 8 (0,75%)  Total: 1066 | 4 (IQR 3-4)  1: 16 (1,47%)  2: 63 (5,81%)  3: 264 (24,33%)  **4: 609 (56,13%)**  5: 122 (11,24%)  6: 11 (1,01%)  Total: 1085 |
|  |  | Age 60+ |  |  |  | 4 (IQR 3-4)  1: 8 (1,66%)  2: 14 (2,91%)  3: 130 (27,03%)  **4: 273 (56,76%)**  5: 44 (9,15%)  6: 12 (2,49%)  Total: 481 | 4 (IQR 3-4)  1: 6 (1,35%)  2: 18 (4,04%)  3: 105 (23,54%)  **4: 259 (58,07%)**  5: 44 (9,87%)  6: 14 (3,14%)  Total: 446 | 4 (IQR 3-4)  1: 8 (1,84%)  2: 17 (3,91%)  3: 106 (24,37%)  **4: 253 (58,16%)**  5: 43 (9,89%)  6: 8 (1,84%)  Total: 435 | 4 (IQR 3-4)  1: 8 (1,94%)  2: 19 (4,61%)  3: 109 (26,46%)  **4: 230 (55,83%)**  5: 40 (9,71%)  6: 6 (1,46%)  Total: 412 |
|  |  | Nursing & Care |  |  |  | 4 (IQR 3-4)  1: 7 (0,85%)  2: 58 (7,06%)  3: 163 (19,85%)  **4: 492 (59,92%)**  5: 97 (11,81%)  6: 4 (0,49%)  Total: 821 | 4 (IQR 3-4)  1: 4 (0,55%)  2: 65 (8,95%)  3: 157 (21,63%)  **4: 426 (58,68%)**  5: 71 (9,78%)  6: 3 (0,41%)  Total: 726 | 4 (IQR 3-4)  1: 3 (0,37%)  2: 47 (5,85%)  3: 153 (19,05%)  **4: 517 (64,38%)**  5: 79 (9,84%)  6: 4 (0,50%)  Total: 803 | 4 (IQR 3-4)  1: 5 (0,62%)  2: 59 (7,34%)  3: 177 (22,01%)  **4: 458 (56,97%)**  5: 104 (12,94%)  6: 1 (0,12%)  Total: 804 |
|  |  | Clinical support |  |  |  | 4 (IQR 3-4)  1: 9 (2,15%)  2: 41 (9,81%)  3: 106 (25,36%)  **4: 224 (53,59%)**  5: 33 (7,89%)  6: 5 (1,20%)  Total: 418 | 4 (IQR 3-4)  1: 5 (1,30%)  2: 44 (11,43%)  3: 96 (24,94%)  **4: 211 (54,81%)**  5: 24 (6,23%)  6: 5 (1,30%)  Total: 385 | 4 (IQR 3-4)  1: 2 (0,48%)  2: 55 (13,22%)  3: 98 (23,56%)  **4: 232 (55,77%)**  5: 28 (6,73%)  6: 1 (0,24%)  Total: 416 | 4 (IQR 3-4)  1: 11 (2,67%)  2: 31 (7,52%)  3: 109 (26,46%)  **4: 228 (55,34%)**  5: 31 (7,52%)  6: 2 (0,49%)  Total: 412 |
|  |  | Clinical (co) treating |  |  |  | 4 (IQR 3-4)  1: 2 (0,65%)  2: 15 (4,85%)  3: 62 (20,06%)  **4: 188 (60,84%)**  5: 40 (12,94%)  6: 2 (0,65%)  Total: 309 | 4 (IQR 3-4)  1: 1 (0,30%)  2: 27 (8,16%)  3: 77 (23,26%)  **4: 198 (59,82%)**  5: 24 (7,25%)  6: 4 (1,21%)  Total: 331 | 4 (IQR 4-4)  1: 1 (0,30%)  2: 23 (6,87%)  3: 59 (17,61%)  **4: 205 (61,19%)**  5: 44 (13,13%)  6: 3 (0,90%)  Total: 335 | 4 (IQR 3-4)  1: 1 (0,29%)  2: 23 (6,61%)  3: 89 (25,57%)  **4: 194 (55,75%)**  5: 36 (10,34%)  6: 5 (1,44%)  Total: 348 |
|  |  | Analytics |  |  |  | 4 (IQR 3-4)  1: 14 (3,30%)  2: 39 (9,20%)  3: 108 (25,74%)  **4: 214 (50,47%)**  5: 45 (10,61%)  6: 4 (0,94%)  Total: 424 | 4 (IQR 3-4)  1: 3 (0,80%)  2: 37 (9,87%)  3: 101 (26,93%)  **4: 216 (57,60%)**  5: 17 (4,53%)  6: 1 (0,27%)  Total: 375 | 4 (IQR 3-4)  1: 5 (1,27%)  2: 38 (9,67%)  3: 95 (24,17%)  **4: 215 (54,71%)**  5: 35 (8,91%)  6: 5 (1,27%)  Total: 393 | 4 (IQR 3-4)  1: 8 (1,91%)  2: 38 (9,09%)  3: 120 (28,71%)  **4: 222 (53,11%)**  5: 27 (6,46%)  6: 3 (0,72%)  Total: 418 |
|  |  | Scientific research & education |  |  |  | 4 (IQR 4-4)  1: 0 (0%)  2: 18 (4,83%)  3: 57 (15,28%)  **4: 213 (57,10%)**  5: 80 (21,44%)  6: 5 (1,34%)  Total: 373 | 4 (IQR 4-4)  1: 0 (0%)  2: 10 (2,58%)  3: 56 (14,47%)  **4: 224 (57,88%)**  5: 93 (24,03%)  6: 4 (1,03%)  Total: 387 | 4 (IQR 4-4)  1: 0 (0%)  2: 11 (3,07%)  3: 49 (13,69%)  **4: 210 (58,66%)**  5: 87 (24,30%)  6: 1 (0,28%)  Total: 358 | 4 (IQR 4-4)  1: 2 (0,50%)  2: 11 (2,74%)  3: 70 (17,41%)  **4: 226 (56,22%)**  5: 88 (21,89%)  6: 5 (1,24%)  Total: 402 |
|  |  | Management |  |  |  | 4 (IQR 4-4)  1: 1 (0,49%)  2: 7 (3,41%)  3: 33 (16,10%)  **4: 126 (61,46%)**  5: 37 (18,05%)  6: 1 (0,49%)  Total: 205 | 4 (IQR 4-4)  1: 3 (1,52%)  2: 8 (4,06%)  3: 29 (14,72%)  **4: 111 (56,35%)**  5: 44 (22,34%)  6: 2 (1,02%)  Total: 197 | 4 (IQR 4-4)  1: 1 (0,50%)  2: 6 (3,01%)  3: 28 (14,07%)  **4: 122 (61,31%)**  5: 41 (20,60%)  6: 1 (0,50%)  Total: 199 | 4 (IQR 4-4)  1: 2 (1,16%)  2: 7 (4,05%)  3: 15 (8,67%)  **4: 115 (66,47%)**  5: 34 (19,65%)  6: 0 (0%)  Total: 173 |
|  |  | Staff, administration, secretariat |  |  |  | 4 (IQR 3-4)  1: 10 (0,90%)  2: 72 (6,48%)  3: 258 (23,22%)  **4: 644 (57,97%)**  5: 118 (10,62%)  6: 9 (0,81%)  Total: 1111 | 4 (IQR 3-4)  1: 12 (1,08%)  2: 69 (6,18%)  3: 235 (21,06%)  **4: 640 (57,35%)**  5: 150 (13,44%)  6: 10 (0,90%)  Total: 1116 | 4 (IQR 3-4)  1: 13 (1,15%)  2: 72 (6,39%)  3: 248 (22,02%)  **4: 657 (58,35%)**  5: 131 (11,63%)  6: 5 (0,44%)  Total: 1126 | 4 (IQR 3-4)  1: 16 (1,41%)  2: 61 (5,37%)  3: 227 (19,98%)  **4: 697 (61,36%)**  5: 124 (10,92%)  6: 11 (0,97%)  Total: 1136 |
|  |  | Facility |  |  |  | 4 (IQR 3-4)  1: 12 (1,83%)  2: 48 (7,32%)  3: 159 (24,24%)  **4: 357 (54,42%)**  5: 72 (10,98%)  6: 8 (1,22%)  Total: 656 | 4 (IQR 3-4)  1: 10 (1,67%)  2: 42 (7,02%)  3: 132 (22,07%)  **4: 334 (55,85%)**  5: 58 (9,70%)  6: 15 (2,51%)  Total: 598 | 4 (IQR 3-4)  1: 11 (1,85%)  2: 47 (7,90%)  3: 136 (22,86%)  **4: 326 (54,79%)**  5: 66 (11,09%)  6: 9 (1,51%)  Total: 595 | 4 (IQR 3-4)  1: 10 (1,75%)  2: 45 (7,89%)  3: 131 (22,98%)  **4: 313 (54,91%)**  5: 62 (10,88%)  6: 9 (1,58%)  Total: 570 |
|  |  | Resident physicians |  |  |  | 4 (IQR 4-5)  1: 0 (0%)  2: 5 (2,78%)  3: 21 (11,67%)  **4: 103 (57,22%)**  5: 50 (27,78%)  6: 1 (0,56%)  Total: 180 | 4 (IQR 4-5)  1: 0 (0%)  2: 5 (3,21%)  3: 10 (6,41%)  **4: 94 (60,26%)**  5: 47 (30,13%)  6: 0 (0%)  Total: 156 | 4 (IQR 4-5)  1: 0 (0%)  2: 2 (1,50%)  3: 13 (9,77%)  **4: 72 (54,14%)**  5: 46 (34,59%)  6: 0 (0%)  Total: 133 | 4 (IQR 4-5)  1: 1 (0,67%)  2: 4 (2,68%)  3: 16 (10,74%)  **4: 94 (63,09%)**  5: 34 (22,82%)  6: 0 (0%)  Total: 149 |
|  |  | Medical specialists |  |  |  | 4 (IQR 4-4)  1: 2 (0,62%)  2: 20 (6,19%)  3: 50 (15,48%)  **4: 183 (56,66%)**  5: 66 (20,43%)  6: 2 (0,62%)  Total: 323 | 4 (IQR 3-4)  1: 3 (0,88%)  2: 26 (7,65%)  3: 68 (20,00%)  **4: 168 (49,41%)**  5: 70 (20,59%)  6: 5 (1,47%)  Total: 340 | 4 (IQR 3-4)  1: 1 (0,31%)  2: 15 (4,72%)  3: 71 (22,33%)  **4: 167 (52,52%)**  5: 64 (20,13%)  6: 0 (0%)  Total: 318 | 4 (IQR 3-4)  1: 2 (0,75%)  2: 10 (3,76%)  3: 55 (20,68%)  **4: 138 (51,88%)**  5: 61 (22,93%)  6: 0 (0%)  Total: 266 |
| Within our team we learn from mistakes | (1) totally disagree; (2) disagree; (3) neutral; (4) agree; (5) totally agree; (6) no opinion | Age<30 |  |  |  | 4 (IQR 3-4)  1: 12 (1,15%)  2: 74 (7,12%)  3: 197 (18,96%)  **4: 626 (60,25%)**  5: 117 (11,26%)  6: 13 (1,25%)  Total: 1039 | 4 (IQR 3-4)  1: 11 (1,19%)  2: 71 (7,65%)  3: 180 (19,40%)  **4: 529 (57,00%)**  5: 124 (13,36%)  6: 13 (1,40%)  Total: 928 | 4 (IQR 3-4)  1: 9 (0,99%)  2: 91 (10,03%)  3: 151 (16,65%)  **4: 532 (58,65%)**  5: 111 (12,24%)  6: 13 (1,43%)  Total: 907 | 4 (IQR 3-4)  1: 11 (1,20%)  2: 78 (8,49%)  3: 153 (16,65%)  **4: 557 (60,61%)**  5: 111 (12,08%)  6: 9 (0,98%)  Total: 919 |
|  |  | Age 30-39 |  |  |  | 4 (IQR 3-4)  1: 15 (1,30%)  2: 99 (8,56%)  3: 198 (17,11%)  **4: 712 (61,54%)**  5: 117 (10,11%)  6: 16 (1,38%)  Total: 1157 | 4 (IQR 3-4)  1: 9 (0,81%)  2: 97 (8,69%)  3: 194 (17,38%)  **4: 663 (59,41%)**  5: 139 (12,46%)  6: 14 (1,25%)  Total: 1116 | 4 (IQR 4-4)  1: 6 (0,52%)  2: 94 (8,20%)  3: 181 (15,79%)  **4: 718 (62,65%)**  5: 137 (11,95%)  6: 10 (0,87%)  Total: 1146 | 4 (IQR 3-4)  1: 19 (1,63%)  2: 79 (6,78%)  3: 219 (18,78%)  **4: 730 (62,61%)**  5: 104 (8,92%)  6: 15 (1,29%)  Total: 1166 |
|  |  | Age 40-49 |  |  |  | 4 (IQR 4-4)  1: 20 (2,03%)  2: 68 (6,90%)  3: 142 (14,42%)  **4: 640 (64,97%)**  5: 107 (10,86%)  6: 8 (0,81%)  Total: 985 | 4 (IQR 4-4)  1: 19 (1,95%)  2: 68 (6,97%)  3: 155 (15,88%)  **4: 630 (64,55%)**  5: 95 (9,73%)  6: 9 (0,92%)  Total: 976 | 4 (IQR 3-4)  1: 15 (1,46%)  2: 78 (7,57%)  3: 166 (16,12%)  **4: 650 (63,11%)**  5: 109 (10,58%)  6: 12 (1,17%)  Total: 1030 | 4 (IQR 4-4)  1: 16 (1,56%)  2: 64 (6,26%)  3: 157 (15,35%)  **4: 684 (66,86%)**  5: 98 (9,58%)  6: 4 (0,39%)  Total: 1023 |
|  |  | Age 50-59 |  |  |  | 4 (IQR 3-4)  1: 12 (1,08%)  2: 84 (7,59%)  3: 209 (18,88%)  **4: 700 (63,23%)**  5: 86 (7,77%)  6: 16 (1,45%)  Total: 1107 | 4 (IQR 3-4)  1: 12 (1,12%)  2: 64 (5,96%)  3: 194 (18,06%)  **4: 688 (64,06%)**  5: 96 (8,94%)  6: 20 (1,86%)  Total: 1074 | 4 (IQR 3-4)  1: 9 (0,84%)  2: 75 (7,04%)  3: 202 (18,95%)  **4: 677 (63,51%)**  5: 95 (8,91%)  6: 8 (0,75%)  Total: 1066 | 4 (IQR 3-4)  1: 12 (1,11%)  2: 80 (7,37%)  3: 201 (18,53%)  **4: 698 (64,33%)**  5: 84 (7,74%)  6: 10 (0,92%)  Total: 1085 |
|  |  | Age 60+ |  |  |  | 4 (IQR 3-4)  1: 3 (0,62%)  2: 30 (6,24%)  3: 105 (21,83%)  **4: 290 (60,29%)**  5: 40 (8,32%)  6: 13 (2,70%)  Total: 481 | 4 (IQR 3-4)  1: 6 (1,35%)  2: 29 (6,50%)  3: 91 (20,40%)  **4: 272 (60,99%)**  5: 37 (8,30%)  6: 11 (2,47%)  Total: 446 | 4 (IQR 4-4)  1: 1 (0,23%)  2: 18 (4,14%)  3: 74 (17,01%)  **4: 294 (67,59%)**  5: 38 (8,74%)  6: 10 (2,30%)  Total: 435 | 4 (IQR 3-4)  1: 4 (0,97%)  2: 26 (6,31%)  3: 81 (19,66%)  **4: 260 (63,11%)**  5: 36 (8,74%)  6: 5 (1,21%)  Total: 412 |
|  |  | Nursing & Care |  |  |  | 4 (IQR 3-4)  1: 7 (0,85%)  2: 70 (8,53%)  3: 155 (18,88%)  **4: 523 (63,70%)**  5: 62 (7,55%)  6: 4 (0,49%)  Total: 821 | 4 (IQR 3-4)  1: 10 (1,38%)  2: 62 (8,54%)  3: 139 (19,15%)  **4: 466 (64,19%)**  5: 45 (6,20%)  6: 4 (0,55%)  Total: 726 | 4 (IQR 3-4)  1: 2 (0,25%)  2: 69 (8,59%)  3: 147 (18,31%)  **4: 520 (64,76%)**  5: 60 (7,47%)  6: 5 (0,62%)  Total: 803 | 4 (IQR 3-4)  1: 8 (1,00%)  2: 76 (9,45%)  3: 150 (18,66%)  **4: 514 (63,93%)**  5: 53 (6,59%)  6: 3 (0,37%)  Total: 804 |
|  |  | Clinical support |  |  |  | 4 (IQR 3-4)  1: 8 (1,91%)  2: 47 (11,24%)  3: 90 (21,53%)  **4: 246 (58,85%)**  5: 22 (5,26%)  6: 5 (1,20%)  Total: 418 | 4 (IQR 3-4)  1: 10 (2,60%)  2: 48 (12,47%)  3: 81 (21,04%)  **4: 218 (56,62%)**  5: 24 (6,23%)  6: 4 (1,04%)  Total: 385 | 4 (IQR 3-4)  1: 7 (1,68%)  2: 56 (13,46%)  3: 72 (17,31%)  **4: 243 (58,41%)**  5: 36 (8,65%)  6: 2 (0,48%)  Total: 416 | 4 (IQR 3-4)  1: 5 (1,21%)  2: 46 (11,17%)  3: 91 (22,09%)  **4: 240 (58,25%)**  5: 25 (6,07%)  6: 5 (1,21%)  Total: 412 |
|  |  | Clinical (co) treating |  |  |  | 4 (IQR 4-4)  1: 2 (0,65%)  2: 22 (7,12%)  3: 52 (16,83%)  **4: 204 (66,02%)**  5: 27 (8,74%)  6: 2 (0,65%)  Total: 309 | 4 (IQR 4-4)  1: 2 (0,60%)  2: 17 (5,14%)  3: 60 (18,13%)  **4: 217 (65,56%)**  5: 30 (9,06%)  6: 5 (1,51%)  Total: 331 | 4 (IQR 4-4)  1: 1 (0,30%)  2: 28 (8,36%)  3: 50 (14,93%)  **4: 227 (67,76%)**  5: 26 (7,76%)  6: 3 (0,90%)  Total: 335 | 4 (IQR 3-4)  1: 3 (0,86%)  2: 20 (5,75%)  3: 80 (22,99%)  **4: 216 (62,07%)**  5: 27 (7,76%)  6: 2 (0,57%)  Total: 348 |
|  |  | Analytics |  |  |  | 4 (IQR 3-4)  1: 7 (1,65%)  2: 49 (11,56%)  3: 82 (19,34%)  **4: 247 (58,25%)**  5: 34 (8,02%)  6: 5 (1,18%)  Total: 424 | 4 (IQR 3-4)  1: 4 (1,07%)  2: 31 (8,27%)  3: 80 (21,33%)  **4: 228 (60,80%)**  5: 26 (6,93%)  6: 6 (1,60%)  Total: 375 | 4 (IQR 3-4)  1: 7 (1,78%)  2: 30 (7,63%)  3: 70 (17,81%)  **4: 245 (62,34%)**  5: 39 (9,92%)  6: 2 (0,51%)  Total: 393 | 4 (IQR 3-4)  1: 9 (2,15%)  2: 25 (5,98%)  3: 86 (20,57%)  **4: 270 (64,59%)**  5: 28 (6,70%)  6: 0 (0%)  Total: 418 |
|  |  | Scientific research & education |  |  |  | 4 (IQR 4-4)  1: 5 (1,34%)  2: 26 (6,97%)  3: 48 (12,87%)  **4: 217 (58,18%)**  5: 63 (16,89%)  6: 14 (3,75%)  Total: 373 | 4 (IQR 4-4)  1: 2 (0,52%)  2: 12 (3,10%)  3: 67 (17,31%)  **4: 241 (62,27%)**  5: 60 (15,50%)  6: 5 (1,29%)  Total: 387 | 4 (IQR 4-4)  1: 1 (0,28%)  2: 17 (4,75%)  3: 41 (11,45%)  **4: 235 (65,64%)**  5: 57 (15,92%)  6: 7 (1,96%)  Total: 358 | 4 (IQR 4-4)  1: 8 (1,99%)  2: 22 (5,47%)  3: 52 (12,94%)  **4: 255 (63,43%)**  5: 57 (14,18%)  6: 8 (1,99%)  Total: 402 |
|  |  | Management |  |  |  | 4 (IQR 4-4)  1: 1 (0,49%)  2: 12 (5,85%)  3: 27 (13,17%)  **4: 142 (69,27%)**  5: 23 (11,22%)  6: 0 (0%)  Total: 205 | 4 (IQR 4-4)  1: 3 (1,52%)  2: 5 (2,54%)  3: 21 (10,66%)  **4: 152 (77,16%)**  5: 16 (8,12%)  6: 0 (0%)  Total: 197 | 4 (IQR 4-4)  1: 0 (0%)  2: 5 (2,51%)  3: 33 (16,58%)  **4: 133 (66,83%)**  5: 27 (13,57%)  6: 1 (0,50%)  Total: 199 | 4 (IQR 4-4)  1: 2 (1,16%)  2: 7 (4,05%)  3: 12 (6,94%)  **4: 136 (78,61%)**  5: 16 (9,25%)  6: 0 (0%)  Total: 173 |
|  |  | Staff, administration, secretariat |  |  |  | 4 (IQR 4-4)  1: 12 (1,08%)  2: 57 (5,13%)  3: 192 (17,28%)  **4: 737 (66,34%)**  5: 95 (8,55%)  6: 18 (1,62%)  Total: 1111 | 4 (IQR 4-4)  1: 7 (0,63%)  2: 59 (5,29%)  3: 198 (17,74%)  **4: 705 (63,17%)**  5: 121 (10,84%)  6: 26 (2,33%)  Total: 1116 | 4 (IQR 4-4)  1: 11 (0,98%)  2: 83 (7,37%)  3: 183 (16,25%)  **4: 697 (61,90%)**  5: 132 (11,72%)  6: 20 (1,78%)  Total: 1126 | 4 (IQR 4-4)  1: 14 (1,23%)  2: 72 (6,34%)  3: 189 (16,64%)  **4: 744 (65,49%)**  5: 107 (9,42%)  6: 10 (0,88%)  Total: 1136 |
|  |  | Facility |  |  |  | 4 (IQR 3-4)  1: 20 (3,05%)  2: 56 (8,54%)  3: 151 (23,02%)  **4: 355 (54,12%)**  5: 57 (8,69%)  6: 17 (2,59%)  Total: 656 | 4 (IQR 3-4)  1: 17 (2,84%)  2: 57 (9,53%)  3: 125 (20,90%)  **4: 323 (54,01%)**  5: 63 (10,54%)  6: 13 (2,17%)  Total: 598 | 4 (IQR 3-4)  1: 15 (2,52%)  2: 60 (10,08%)  3: 138 (23,19%)  **4: 317 (53,28%)**  5: 55 (9,24%)  6: 10 (1,68%)  Total: 595 | 4 (IQR 3-4)  1: 12 (2,11%)  2: 53 (9,30%)  3: 120 (21,05%)  **4: 309 (54,21%)**  5: 63 (11,05%)  6: 13 (2,28%)  Total: 570 |
|  |  | Resident physicians |  |  |  | 4 (IQR 4-4)  1: 0 (0%)  2: 7 (3,89%)  3: 31 (17,22%)  **4: 119 (66,11%)**  5: 22 (12,22%)  6: 1 (0,56%)  Total: 180 | 4 (IQR 4-4)  1: 0 (0%)  2: 5 (3,21%)  3: 19 (12,18%)  **4: 103 (66,03%)**  5: 28 (17,95%)  6: 1 (0,64%)  Total: 156 | 4 (IQR 4-4)  1: 0 (0%)  2: 3 (2,26%)  3: 21 (15,79%)  **4: 86 (64,66%)**  5: 21 (15,79%)  6: 2 (1,50%)  Total: 133 | 4 (IQR 4-4)  1: 0 (0%)  2: 8 (5,37%)  3: 19 (12,75%)  **4: 102 (68,46%)**  5: 18 (12,08%)  6: 2 (1,34%)  Total: 149 |
|  |  | Medical specialists |  |  |  | 4 (IQR 4-4)  1: 4 (1,24%)  2: 15 (4,64%)  3: 37 (11,46%)  **4: 215 (66,56%)**  5: 51 (15,79%)  6: 1 (0,31%)  Total: 323 | 4 (IQR 4-4)  1: 3 (0,88%)  2: 24 (7,06%)  3: 41 (12,06%)  **4: 202 (59,41%)**  5: 68 (20,00%)  6: 2 (0,59%)  Total: 340 | 4 (IQR 4-4)  1: 1 (0,31%)  2: 20 (6,29%)  3: 50 (15,72%)  **4: 198 (62,26%)**  5: 49 (15,41%)  6: 0 (0%)  Total: 318 | 4 (IQR 4-4)  1: 4 (1,50%)  2: 12 (4,51%)  3: 29 (10,90%)  **4: 186 (69,92%)**  5: 34 (12,78%)  6: 0 (0%)  Total: 266 |
