# Supplementary material for: Uncovering gaps in workforce well-being: a national look at survey practice in Dutch university medical centres – an exploratory quantitative study
Source: BMJ Open. 2025 Jul 18;15(7):e094939. doi: 10.1136/bmjopen-2024-094939 (PMC12273149; doi:10.1136/bmjopen-2024-094939)
Supplement: Supplementary file 6 [file bmjopen-2024-094939.reviewer_comments.pdf]

PEER REVIEW HISTORY

BMJ Open publishes all reviews undertaken for accepted manuscripts. Reviewers are asked to complete a checklist review form and are provided with free text boxes to elaborate on their assessment. These free text comments are reproduced below.

ARTICLE DETAILS

Title (Provisional)

Uncovering gaps in workforce well-being: a national look at survey practice in Dutch university medical centers: an exploratory quantitative study

Authors

Boskma, Amber; Oerbekke, Michiel S; Hooft, Lotty; Franx, Arie; Schaufeli, Wilmar; van der Laan, Maarten J.

VERSION 1 - REVIEW

**Reviewer**	**1**
**Name**	**Kirkland, Chelsey**
**Affiliation**	**University of Minnesota**
**Date**	**25-Nov-2024**
**COI**	**None**

Thank you for the opportunity to review "Uncovering gaps in workforce well-being, a national look at survey practice in Dutch university medical centers: a mixed-methods study." This study aims to understand healthcare professionals' work experiences across several years and identify patterns of well-being. While the topic is critical, there are several major issues with the manuscript as written. First, the title says that it is a mixed-methods study, but there is only quantitative data. Secondly, the discussion says that the validity and reliability of the surveys are poor and may have led to "consistency problems" and that "conclusions should be interpreted with caution." If data collection instruments cannot be trusted, neither can the data produced. Lastly, the main intent of the manuscript was to provide insights into work experiences of healthcare professionals, but these "insights" were never discussed, even in the "practical implications" section.

**Reviewer**	**2**
**Name**	**Fu, Wenning**
**Affiliation**	**Huazhong University of Science and Technology Tongji Medical College**

**Date**	**06-Jan-2025**
**COI**	**None**

1.Tables: All tables should be three-wire tables. Please refer to other articles to modify the format of all tables in the article.

2.Abstract: Include the number of participants and hospitals more prominently in the abstract to give a better sense of the scale of the study.

3.Introduction: Strengthen the connection between the background information on healthcare workforce issues and the specific aims of this study. Clearly explain how the study fills the research gap in the context of previous studies. Provide more recent and specific references to support the statements about the importance of workforce well-being and the problems faced by healthcare professionals.

4.Results: Provide more in-depth analysis of the differences and similarities between the two hospitals. Explain why certain groups showed different responses in different hospitals and what implications these differences have.

5.Conclusion: Summarize the key points of the study more succinctly and emphasize the practical significance of the findings. Clearly state the main takeaways for the healthcare industry. Suggest future research directions more explicitly. For example, mention specific research questions that could be explored further based on the limitations of this study.

**Reviewer**	**3**
**Name**	**Woodward, Kyla F.**
**Affiliation**	**University of Washington, Child, Family, and Population Health Nursing**
**Date**	**08-Jan-2025**
**COI**	**None**

This manuscript touches on an extremely important concept, that of healthcare workforce wellbeing. I think the authors' take on studying the concept is unique in that it looks across several centers to see what is being measured and if that is adequate to address wellbeing. However, this purpose is somewhat convoluted by the inclusion of the actual data analysis from these surveys rather than a pure focus on if measurements are adequate, and I suspect that this lack of clarity may also have impacted other organizations' willingness to participate in the study. I think that the paper should actually be broken into 2 manuscripts—one focused on the survey logistics and assessing if each hospital or individual survey has adequately measured constructs according to the JD-R, and one focused on the actual survey results. Please find specific notes below.

Introduction

In general, this section needs to include more definitions of concepts such as absenteeism and turnover. It also needs to be clearer in terms of which elements are part of the context or work environment and which are the results to make a stronger case. The flow of the first paragraph is confusing and could be strengthened by more clearly outlining these factors as cause/effect. The final sentence beginning with 'therefore' mentions well-being and engagement, neither of which have been explicitly defined in the preceding text.

Paragraph 2 – what type of organizational change and what does 'long-term' mean? Months/years? The term is quite vague but in the rest of the paragraph there seems to be specific things in mind as that should be confidential/accessible, which suggests that they are actually targeted to individuals and not the system.

The study purpose is not completely clear. It feels like a mix between examining if the surveys are congruent and the actual contents of the survey.

Methods

The study design is not explicitly stated, and I think this is partly due to the duality of the project as both a review of constructs and a secondary analysis of data.

Data collection: Why do multiple time points from each hospital need to be included? If hospital A is using different surveys, why is that? And won't the results from the 3 surveys be overlapping since some workers are filling out the different surveys? For hospital B, if they are the same survey and there are no comparisons between hospitals, why include so many timepoints? Isn't there a risk of having workers with repeated measurements? After referencing Figure 1, there is a statement 'the surveys were constructed' and I'm not sure what survey is being referenced. Is it the surveys the hospitals are using? Or a data collection tool?

I appreciate the inclusion of so many different kinds of workers and not just clinicians!

Data handling: I have serious concerns with the excluded categories….those are vital components of the context and work environment. Why can't these be included in the Table 3 results?

Data analysis: I question the validity of tracking outcomes across different surveys (hospital A) or different participants (hospital B).

Ethics approval. Although the protections seem adequate, I don't believe this counts as 'anonymous data' since it is linked with some demographics. The 'data manager officer was consulted' does not include any statement of their recommendations or permissions for using the data.

Results

While the data is not subject to selection bias in the secondary analysis, it is absolutely subject to selection bias in terms of who completed the surveys as a whole and who

completed specific items in the surveys, and this bias is not adequately addressed. Again, it is troubling to talk about all the surveys from each hospital without acknowledging the possibility of significant overlap within each hospital and the potential for skewed results.

The text list of descriptive results categories is unhelpful—this would be better represented in a Figure mapping these elements onto the JD-R. While it is helpful to see the breakdown of elements by JD-R category, the short paragraphs for each item are better represented in Table 3 and efforts should be made to be more concise here, e.g. 'participants from hospital A tended to agree that co-worker support (and other constructs within this category) were present, with no notable changes over time'. I will refrain from commenting on the rest of the individual sections at this point.

Table 3 is extremely helpful and should provide much of the actual results, although with each category itemized it is difficult to justify excluding the other categories as previously addressed.

Due to methodological weaknesses, I refrained from detailed comments on the discussion. I would happily review subsequent versions of the paper if the authors decide to revise, which I recommend. I think it's important to understand who is measuring what when we talk about health workforce wellbeing.

**Reviewer**	**4**
**Name**	**You, Zhiying**
**Affiliation**	**University of Colorado Denver School of Medicine,**
**Medicine**	
**Date**	**16-Jan-2025**
**COI**	**None**

---

1. A title should be included in the figure. Pease add a title.

2. In the Data analysis section, please state clearly how the repeated data has been used in the calculation of the descriptive statistics, such as per hospital and per measurement moment.

3. About page 7, line 19-20: "A total of 31.862 completed surveys were included, encompassing 11.862 for hospital A and 19.687 for hospital B": please clarify the two numbers 11.862 and 19.687, what they really meant?

VERSION 1 - AUTHOR RESPONSE

Reviewer: 1

Dr. Chelsey Kirkland, University of Minnesota

Thank you for the opportunity to review "Uncovering gaps in workforce well-being, a national look at survey practice in Dutch university medical centers: a mixed-methods study." This study aims to understand healthcare professionals' work experiences across several years and identify patterns of well-being. While the topic is critical, there are several major issues with the manuscript as written. First, the title says that it is a mixed-methods study, but there is only quantitative data.

Thank you for your comment. We have changed the title into: 'Uncovering gaps in workforce well-being: a national look at survey practice in Dutch university medical centers: an exploratory quantitative study'

Secondly, the discussion says that the validity and reliability of the surveys are poor and may have led to "consistency problems" and that "conclusions should be interpreted with caution." If data collection instruments cannot be trusted, neither can the data produced.

Thank you for your comment. We agree with your observation and acknowledge that relying on non-validated surveys is a limitation of our study. However, we intentionally chose to evaluate and use existing surveys to maximize data reuse and avoid adding to the workload of healthcare professionals, who are already facing high workloads. This pragmatic approach inevitably comes with drawbacks, including potential concerns regarding validity and reliability.

That said, we believe this decision reflects the reality of daily practice, highlighting the need for improvement in survey content and measurement methods. It underscores that the content of surveys and the methods of measurement should be reconsidered. Additionally, we have done everything possible within our constraints. We avoided potentially misleading mean analyses, which are commonly used despite the categorical nature of Likert-scale data. Instead, we presented medians and distributions to apply the most appropriate statistical approach, even in the presence of heterogeneity.

Throughout the manuscript, we have consistently underscored the limitations of our approach and stressed that the findings should be interpreted with caution. We hope this transparent acknowledgement, along with our effort to highlight the complexity of current survey practices, measurement methods, and the pressing need for improvement, aligns with the approaches in daily practices and the goals of our study.

Lastly, the main intent of the manuscript was to provide insights into work experiences of healthcare professionals, but these "insights" were never discussed, even in the "practical implications" section.

Thank you for your feedback. We have changed the aim into: ''The aim of this study is to evaluate the content of employee surveys currently used in university medical centers in the Netherlands from a well-being perspective and to analyze the survey results at a national level.''

Moreover, we have addressed the discussion in an effort to improve the quality and readability. We have rewritten the main findings and adjusted the structure, aligning it better with the aim of the study, which is now dual in nature and articulated in two phases. We hope that you can appreciate the changes made.

Reviewer: 2
Dr. Wenning Fu, Huazhong University of Science and Technology Tongji Medical College
Comments to the Author:
1.Tables: All tables should be three-wire tables. Please refer to other articles to modify the format of all tables in the article.

Thank you for your comment. We have reformatted all the tables to three-wire.

2.Abstract: Include the number of participants and hospitals more prominently in the abstract to give a better sense of the scale of the study.

Thank you for your suggestion. We moved the sentence about the fact that two hospitals participated to the results section of the abstract. Moreover, we have added the amount of included surveys as follows: 'Two UMCs participated and 31.862 completed surveys were included.' We hope this is more clear now.

3.Introduction: Strengthen the connection between the background information on healthcare workforce issues and the specific aims of this study. Clearly explain how the study fills the research gap in the context of previous studies. Provide more recent and specific references to support the statements about the importance of workforce well-being and the problems faced by healthcare professionals.

Thank you for your feedback. We have added information at the beginning of the paragraph, including more recent references to better place concepts and frameworks within a theoretical context. The following text has been added:

"The Job Demands-Resources model (JD-R model) offers a dynamic framework that highlights well-being at work processes. The JD-R model components are 'demands' (e.g., stress, workload, conflicts), 'resources' (e.g., support, development opportunities, team atmosphere), 'leadership' (e.g., inspiring, connecting), and 'personal resources' (e.g., motivation, resilience). The balance in the different model components contributes to more positive well-being (e.g., job satisfaction) or more negative well-being (e.g., burnout), reflected in outcomes such as commitment, intention to stay, and work performance."

To make the relevance of this study clearer and explain how it addresses the research gap, we have added the following information:

"Nowadays, employee surveys are intended to create insight and should be used to improve the working environment, well-being, and engagement of employees. Many employee surveys exist, and previous research has focused on the content, use, and implementation of surveys and survey results. However, contexts and perceptions of healthcare professionals (HCP) change over time. It is not advisable to develop new tools, but rather to analyze the current ones and optimize them. Furthermore, approaches are often focused on work environment experiences, which means that the well-being of HCPs is less emphasized."

We hope that you agree with these revisions and that they align with your suggestions for improvement, enhancing both the quality and readability of the manuscript.

4.Results: Provide more in-depth analysis of the differences and similarities between the two hospitals. Explain why certain groups showed different responses in different hospitals and what implications these differences have.

Thank you for your valuable suggestion. We understand your interest in exploring the differences between the two hospitals. However, the survey constructs, questions, and response options differed considerably, making in-depth comparative analysis infeasible.

Despite these limitations, we have made every effort to conduct a meaningful analysis within our constraints. Typically, analyses within hospitals are based on mean scores. However, given the

categorical nature of the response options, using means can be misleading. Instead, we presented medians and score distributions to provide a more appropriate and nuanced representation of the results.

We hope this clarifies our methodological choices and that the revised presentation of the results is now more acceptable. In particular, we restructured the results section to distinguish more clearly between phases 1 and 2, better aligning with the study's objectives. The presentation of results has also been made more concise, and we provided a clearer reference to Table 3.

We considered including a paragraph on differences between hospitals in the discussion. However, aside from variations in how the surveys were structured and distributed, we did not find any meaningful differences. Nevertheless, we adjusted the following text in the discussion section to acknowledge potential influences:

Survey design and methodological variation
Substantial variation was found across UMCs in terms of survey content, item phrasing, response options, timing, and frequency. While surveys covered a wide array of topics, the focus of the questions often reflected strategic priorities of hospital boards rather than the focus of implemented interventions. These findings align with existing literature describing the trade-offs between comprehensive validated instruments and shorter, pragmatic tools that are easier to administer but less robust psychometrically [24,28]. The diversity of survey designs may be influenced by local leadership styles, cultural norms, and shifting organizational priorities. While this flexibility can help capture emerging topics—such as inclusivity and psychological safety [22]—it limits comparability across organizations and over time, undermining the potential for data-informed decision-making.

Challenges in interpreting results
The exploratory analysis of survey results from Hospital A highlighted several domains—particularly job control, learning and development, team effectiveness, and inspiring leadership—as potential areas for improvement. Items in these domains scored a median of 2 or 3, compared to 4 on most other topics. While this provides a starting point for discussion, the interpretation must be cautious due to limitations in survey design, measurement reliability, and external validity. Moreover, these data only represent a subset of UMC employees and likely suffer from response bias and overlap in respondents.

Notably, we found minimal differences in outcomes over time, including during and after the COVID-19 period. This is inconsistent with literature showing significant declines in well-being among healthcare professionals during the pandemic [32,33]. Several explanations are possible: survey items may lack sensitivity to change, the surveys may not capture key stressors, or a relatively short timeframe and socially desirable responding may have masked real variation. These issues point to the need for clinimetric research into the validity, reliability, and responsiveness of existing tools [34].

Misalignment between measurement and action
A key finding is the disconnect between what is measured and what is targeted by interventions. Most survey items focus on job resources—such as supervisory support and team climate—while organizational interventions tend to target personal resources like resilience or stress management. This misalignment may partly explain why organizations struggle to measure intervention impact or drive meaningful change through survey data.

Strengths and limitations
This study offers early insights into both the content of well-being surveys and the patterns they reveal. A strength of our approach is the descriptive use of distributions and medians instead of means, which are often inappropriately applied to ordinal data. However, significant limitations

remain. The use of non-validated and heterogeneous survey instruments decreases internal validity and makes cross-institutional comparison difficult. The inductive categorization process, although conducted independently by the authors, introduces the risk of inconsistency and subjectivity. Furthermore, only two of the seven Dutch UMCs participated, greatly reducing generalizability. Overlapping respondents and selection bias may have further skewed results. Combined, these limitations constrain our ability to draw robust conclusions and diminish the utility of findings for policy or practice.

We hope these adjustments have improved both the clarity and the quality of the manuscript.

5.Conclusion: Summarize the key points of the study more succinctly and emphasize the practical significance of the findings. Clearly state the main takeaways for the healthcare industry. Suggest future research directions more explicitly. For example, mention specific research questions that could be explored further based on the limitations of this study.

Thank you for your point. We rewrote the conclusion as follows:

Currently, employee well-being surveys in Dutch UMCs show significant methodological variation, limiting their usefulness for monitoring work experience and informing policy. While they may support internal reflection, their capacity to guide targeted intervention is undermined by weak measurement validity, lack of alignment with organizational actions, and limited comparability across centers.

Job control appears to be an area warranting further attention, though interpretation is constrained by methodological issues. The lack of meaningful variation in survey outcomes over time raises important questions about the appropriateness of current instruments and the assumptions guiding their use.

Future research should focus on developing a consensus-based core outcome set (e.g., via a Delphi study), and explore why validated tools are underutilized and collaboration is minimal. Rather than developing new instruments, efforts should center on improving use and integration of existing tools. To support evidence-based people management, the rigor of patient care research must be mirrored in the way we evaluate and support the healthcare workforce.

Reviewer: 3
Dr. Kyla F. Woodward, University of Washington
Comments to the Author:
This manuscript touches on an extremely important concept, that of healthcare workforce wellbeing. I think the authors' take on studying the concept is unique in that it looks across several centers to see what is being measured and if that is adequate to address wellbeing. However, this purpose is somewhat convoluted by the inclusion of the actual data analysis from these surveys rather than a pure focus on if measurements are adequate, and I suspect that this lack of clarity may also have impacted other organizations' willingness to participate in the study. I think that the paper should actually be broken into 2 manuscripts—one focused on the survey logistics and assessing if each hospital or individual survey has adequately measured constructs according to the JD-R, and one focused on the actual survey results. Please find specific notes below.

Introduction
In general, this section needs to include more definitions of concepts such as absenteeism and turnover. It also needs to be clearer in terms of which elements are part of the context or work environment and which are the results to make a stronger case. The flow of the first paragraph is confusing and could be strengthened by more clearly outlining these factors as cause/effect. The final sentence beginning with 'therefore' mentions well-being and engagement, neither of which

have been explicitly defined in the preceding text.
Paragraph 2 – what type of organizational change and what does 'long-term' mean? Months/years? The term is quite vague but in the rest of the paragraph there seems to be specific things in mind as that should be confidential/accessible, which suggests that they are actually targeted to individuals and not the system.
The study purpose is not completely clear. It feels like a mix between examining if the surveys are congruent and the actual contents of the survey.

Thank you for your thoughtful and constructive feedback. We greatly appreciate your recognition of the importance of this topic and your suggestions for improving the clarity and focus of the manuscript. We have revised the Introduction to provide clearer definitions of key concepts such as absenteeism, turnover, well-being, and engagement. We have also strengthened the cause-and-effect reasoning by explicitly outlining how specific contextual and environmental factors influence outcomes related to workforce well-being.

Additionally, we added a new paragraph that positions these concepts within a theoretical framework (specifically referencing the JDR model), which we hope will improve clarity and grounding. The paragraph that previously used the term "long-term" has been rewritten to include specific examples, removing ambiguous language and adding appropriate references to support our statements.

In response to your comment on the study purpose, we agree that the previous version may have lacked focus. We have now clarified the aim as follows:

" The aim of this study is to evaluate the content of employee surveys currently used in university medical centers in the Netherlands from a well-being perspective and to analyze the survey results at a national level.''

We understand your suggestion to consider splitting the manuscript into two separate papers: one focusing on survey content in relation to the JD-R model, and one presenting the national-level results. We agree this is a valuable consideration and are currently evaluating the feasibility of this for future work. For this manuscript, we have worked to sharpen the focus while maintaining coherence between both elements, and we hope the revised version addresses the concerns regarding clarity and scope in one manuscript.

We hope that the revisions enhance the clarity, rigor, and relevance of our study and that they align with your suggestions for improvement, enhancing both the quality and readability of the manuscript.

Methods
The study design is not explicitly stated, and I think this is partly due to the duality of the project as both a review of constructs and a secondary analysis of data.

Thank you for your comments. As a first step, we have added the following to the 'study design' section: ''The study consisted of two parts: in the preparation phase (1) the content of employee surveys was assessed and in the execution phase (2) the survey results were explored of two university medical centers (UMCs).'' In the 'setting and respondents' section we reversed the sentences and elaborated the first one as follows: '' All seven UMCs were approached to participate in the study and share employee survey items and employee survey results.''

For more clarity, we removed '' Two UMCs were willing and in the opportunity to participate.'' From the ''results'' to the ''data collection''. Furthermore, for more transparency we added: 'This study

aimed to re-use existing data. The advantage of this approach was the ability to evaluate existing surveys without placing an additional burden on employees to obtain a national-level overview. It was assumed in advance that well-being would be assessed, at least in part, in the available surveys. This would allow for a comparable analysis using data that was already collected.' and 'Survey items were qualitatively categorized by AB and MO following the JD-R model.'

Data collection: Why do multiple time points from each hospital need to be included?

We are sorry that this was not clear. We included various timepoints to discover patterns over time, as mentioned in the analysis section. Moreover, we wanted to include more timepoints because of the COVID-19 period and the seasonal influences. We elaborated the following: 'Data from 2020 to 2023 were collected to identify patterns over time and to account for possible fluctuations due to COVID-19 and seasonal variation.'

If hospital A is using different surveys, why is that? And won't the results from the 3 surveys be overlapping since some workers are filling out the different surveys? For hospital B, if they are the same survey and there are no comparisons between hospitals, why include so many timepoints? Isn't there a risk of having workers with repeated measurements?

Our apologies that this was insufficiently clear. In the data analysis section we added the following about overlapping results: 'Within hospitals and across measurement moments, data from the same individuals may be present. Repeated measures were included in the analysis; however, due to the anonymous nature of the data, it was not possible to link individual responses over time. Partly because of that, the data are presented descriptively, aiming to uncover patterns over the years.

After referencing Figure 1, there is a statement 'the surveys were constructed' and I'm not sure what survey is being referenced. Is it the surveys the hospitals are using? Or a data collection tool?

Sorry that this was confusing. We did not develop a survey, we adjusted the sentence into: 'For phase one, survey items were qualitatively categorized by AB and MO following the JD-R model to explore if questions were included addressing: 'Job demands' (e.g., workload, work-life balance), 'Job resources' (e.g., sustainable employability, recognition/appreciation, job content, development opportunities, psychological safety, support, team), 'Leadership' (e.g., autonomy, leadership), 'Employee well-being' (e.g., job satisfaction), and 'Outcomes' (e.g., health, engagement, eNPS (employee Net Promotor Score), intention to leave (the profession), patient safety, improvement).'

I appreciate the inclusion of so many different kinds of workers and not just clinicians!

Thank you for your compliment.

Data handling: I have serious concerns with the excluded categories....those are vital components of the context and work environment. Why can't these be included in the Table 3 results?

We agree with what you mention. However, we excluded some categories because of the idea to explore data over time. We added this within the data handling section.

Data analysis: I question the validity of tracking outcomes across different surveys (hospital A) or different participants (hospital B).

This concern is valid. The comparison across different surveys (Hospital A) and different participants (Hospital B) was therefore approached descriptively, aiming to explore broad patterns over time.

Ethics approval. Although the protections seem adequate, I don't believe this counts as 'anonymous data' since it is linked with some demographics. The 'data manager officer was consulted' does not include any statement of their recommendations or permissions for using the data.

The data can be considered anonymous because the basic characteristics were collected in categories. Each group was sufficiently large, which makes the data non-traceable to individuals.

We have added the following to the ethics section: 'The data manager officer was consulted for advice and confirmed that the use of the data was permitted, provided that basic characteristics were collected in categories. Each group was determined to be sufficiently large, which makes the data non-traceable. Using anonymous data eliminates informed consent.'

To avoid duplicate information, we removed the following from the data collection section: 'To prevent employees from being identifiable, it was important that..'

Moreover, we adjusted 'collected' to 'provided' in the following sentence to clarify that the data were not anonymized by the researchers themselves: 'Anonymized data at the employee-level was provided through the hospitals' Human Resource advisors of the hospitals.'

Results
While the data is not subject to selection bias in the secondary analysis, it is absolutely subject to selection bias in terms of who completed the surveys as a whole and who completed specific items in the surveys, and this bias is not adequately addressed. Again, it is troubling to talk about all the surveys from each hospital without acknowledging the possibility of significant overlap within each hospital and the potential for skewed results.

Thank you for your comment. We agree with your point. We have adjusted the following in the strengths and limitations section:

This study offers early insights into both the content of well-being surveys and the patterns they reveal. A strength of our approach is the descriptive use of distributions and medians instead of means, which are often inappropriately applied to ordinal data. However, significant limitations remain. The use of non-validated and heterogeneous survey instruments decreases internal validity and makes cross-institutional comparison difficult. The inductive categorization process, although conducted independently by the authors, introduces the risk of inconsistency and subjectivity. Furthermore, only two of the seven Dutch UMCs participated, greatly reducing generalizability. Overlapping respondents and selection bias may have further skewed results. Combined, these limitations constrain our ability to draw robust conclusions and diminish the utility of findings for policy or practice.

The text list of descriptive results categories is unhelpful—this would be better represented in a Figure mapping these elements onto the JD-R. While it is helpful to see the breakdown of elements by JD-R category, the short paragraphs for each item are better represented in Table 3 and efforts should be made to be more concise here, e.g. 'participants from hospital A tended to agree that co-worker support (and other constructs within this category) were present, with no notable changes over time'. I will refrain from commenting on the rest of the individual sections at this point.
Table 3 is extremely helpful and should provide much of the actual results, although with each category itemized it is difficult to justify excluding the other categories as previously addressed.

Thank you for your suggestion. We agree with what you proposed. The structure of the results has been adjusted, making phases 1 and 2 more distinct. This also better aligns with the aim now. Additionally, as you indicated, the results have been made more concise and a clearer reference to

Table 3 has been provided. We hope the adjustments have improved the quality and readability of the paper.

Moreover, we understand that you prefer a figure. However, we find it difficult to find a suitable figure because there is little difference to be seen. For example, in a radar chart or bar chart, sometimes you cannot see a line or bar due to missing data, or the difference is not discernible because the difference is so small. Therefore, a table still seems like the best option because it allows us to be transparent, display a lot of heterogeneous data, and show little variation over time. If you have other suggestions or ideas for a figure, we are definitely open to them.

Due to methodological weaknesses, I refrained from detailed comments on the discussion. I would happily review subsequent versions of the paper if the authors decide to revise, which I recommend. I think it's important to understand who is measuring what when we talk about health workforce wellbeing.

We understand your point. Nevertheless, we have addressed the discussion in an effort to improve it. We have rewritten the main findings and adjusted the structure, aligning it more closely with the aim of the study, which is now dual in nature and articulated in two phases. I hope that you can appreciate the changes made.

Reviewer: 4
Dr. Zhiying You, University of Colorado Denver School of Medicine, Colorado School of Public Health
Comments to the Author:
1. A title should be included in the figure. Pease add a title.

Sorry that this was missing. We added the title.

2. In the Data analysis section, please state clearly how the repeated data has been used in the calculation of the descriptive statistics, such as per hospital and per measurement moment.

Thank you for your comment. In the data analysis section we added the following about overlapping results: 'Within hospitals and across measurement moments, data from the same individuals may be present. Repeated measures were included in the analysis; however, due to the anonymous nature of the data, it was not possible to link individual responses over time. Partly because of that, the data are presented descriptively, aiming to uncover patterns over the years.'

3. About page 7, line 19-20: "A total of 31.862 completed surveys were included, encompassing 11.862 for hospital A and 19.687 for hospital B": please clarify the two numbers 11.862 and 19.687, what they really meant?

Sorry that this was insufficiently clear. We added the following: 'This corresponds to 3603–5056 fully completed surveys at each measurement moment.'

VERSION 2 - REVIEW

**Reviewer**	**2**
**Name**	**Fu, Wenning**
**Affiliation**	**Huazhong University of Science and Technology Tongji Medical College**

Date **19-May-2025**

COI

Reviewer: Comments to Author (required) :

Dear editor,

Thank you so much for giving me the opportunity to contribute to the BMJ Open.

1.Introduction: The introduction has provided the challenges facing the health system and the importance of employee benefits, but can further emphasize the unique role and responsibility of University Medical Centers in the national health system to help readers better understand the importance and context of the study.

2.Method: The description of data collection and analysis methods can be more detailed. It is recommended to summarize and supplement the test methods and results of the reliability and validity of the questionnaire to ensure the quality and reliability of the collected data.

3.Discussion: To explore the implications, possible explanations, and comparisons with other studies of these results in greater depth.

4.References: Please ensure that all key points are supported by appropriate literature and that the citation format is in accordance with journal requirements. Note to check for repeated citations of the same document in the same location, such as “page 9, line 51”.

Reviewer **3**

Name **Woodward, Kyla F.**

Affiliation **University of Washington, Child, Family, and Population Health Nursing**

Date **22-Apr-2025**

COI

This revision offered great improvement in content and flow over the previous version. I appreciate the authors addressing all the reviews and offer a few final suggestions:

I feel the introduction got a bit lengthy and difficult to follow. Streamlining and some subheadings to help organize flow would be greatly appreciated.

In the results section, it was difficult to interpret the numerical results (medians/IQRs) as so many of the questions had different scales. If editors agree, I would consider not reporting those results in the text and instead making statements about the general agreement/disagreement, satisfaction, or whatever other quality is being measured in the

scale rather than the median. I would prefer to see ‘respondents/workers at hospital A or B’ rather than ‘hospital A responded’.

In the challenges in interpreting results section, again the information needed to understand the meaning of a median 2 or 3 compared to 4 is missing and needs explanation.

Thank you for the opportunity to review this revision.

VERSION 2 - AUTHOR RESPONSE

Reviewer: 3

1. I feel the introduction got a bit lengthy and difficult to follow. Streamlining and some subheadings to help organize flow would be greatly appreciated.

Thank you for your feedback. We agree with your suggestion and have therefore made the proposed adjustments. We revised the introduction to make it more concise and impactful, reducing its length by approximately 100 words. In addition, we added subheadings to improve the readability and structure of the introduction. With these changes, we hope the introduction has become clearer and more effective.

2. In the results section, it was difficult to interpret the numerical results (medians/IQRs) as so many of the questions had different scales. If editors agree, I would consider not reporting those results in the text and instead making statements about the general agreement/disagreement, satisfaction, or whatever other quality is being measured in the scale rather than the median. I would prefer to see ‘respondents/workers at hospital A or B’ rather than ‘hospital A responded’.

Thank you for your suggestion. We agree with your proposed phrasing and have revised the results section accordingly. We chose not to remove the medians and IQRs, as these ranges provide insight into the distribution and answers. They also help to identify which elements scored lower, with medians of 2 or 3. Furthermore, in the previous revision, the tables were moved to the additional files at the request of the technical checks, which makes it even more important to describe the findings in greater detail within the text.

However, we have placed greater emphasis on describing and interpreting the degree of agreement or disagreement, while rephrasing the results in a more factual tone rather than focusing on how participants responded. We trust these adjustments have contributed to improving the clarity and quality of the results section.

3. In the challenges in interpreting results section, again the information needed to understand the meaning of a median 2 or 3 compared to 4 is missing and needs explanation.

Thank you for your suggestion. We have added the following for more clarity at the start of section: Phase 2: Survey results at a national level

‘Overall, the surveys use 5-point Likert-type scales, where response options typically range from (1) totally disagree to (5) totally agree. A sixth option (6) is included and varies by item as either no opinion or not applicable. A median score of 4 indicates agreement or a positive experience. A

median of 3 reflects neutral or mixed perceptions. A median of 2 indicates disagreement or a negative experience.'

Reviewer: 2

1. Introduction: The introduction has provided the challenges facing the health system and the importance of employee benefits, but can further emphasize the unique role and responsibility of University Medical Centers in the national health system to help readers better understand the importance and context of the study.

Thank you for your suggestion. We added the following in the second to last paragraph of the introduction:

Their role in treating the most complex cases and training future healthcare professionals increases the organizational demands and highlights the importance of maintaining a healthy, sustainable workforce. This makes structured evaluation of well-being through employee surveys especially valuable.

2. Method: The description of data collection and analysis methods can be more detailed. It is recommended to summarize and supplement the test methods and results of the reliability and validity of the questionnaire to ensure the quality and reliability of the collected data.

Thank you for your comment. We understand your concern and have addressed it by making several additions. Specifically, we have added that:

- Data were collected through Human Resource advisors.
- We included as many relevant details as possible regarding the data analysis.
- In the strengths and limitations section (both in the abstract and the discussion), we clarified that non-validated questionnaires were used. Now, we have emphasized this point extra in the data collection paragraph, where we explain our rationale for this approach. Namely, to make optimal use of existing data, evaluate existing surveys, and avoid placing additional burden on healthcare professionals.

We hope you agree with the changes made.

3. Discussion: To explore the implications, possible explanations, and comparisons with other studies of these results in greater depth.

Our apologies that this was not sufficient. We have expanded the discussion by attempting to better explain the results, place them in the context of other research, and include additional references and practical recommendations. We hope these revisions have improved the discussion and that it is now satisfactory.

4. References: Please ensure that all key points are supported by appropriate literature and that the citation format is in accordance with journal requirements. Note to check for repeated citations of the same document in the same location, such as "page 9, line 51".

Thank you for your attentiveness. We reviewed the requirements again and made some adjustments to the reference formatting accordingly. Reference 32, which you pointed out on page 51, was mistakenly included due to tracked changes and the link with Mendeley. We have now removed it.
